# Correction: *mlh3* mutations in baker’s yeast alter meiotic recombination outcomes by increasing noncrossover events genome-wide

**DOI:** 10.1371/journal.pgen.1007067

**Published:** 2017-10-24

**Authors:** The PLOS Genetics Staff

## Notice of republication

This article was republished on October 5, 2017, to correct a global change where instances of “*MLH3*” were incorrectly typeset as “*mlh3*”. The publisher apologizes for the errors. Please download this article again to view the correct version. The originally published, uncorrected article and the republished, corrected articles are provided here for reference.

## Supporting information

S1 File Originally published, uncorrected article. (PDF)

S2 File Republished, corrected article. (PDF)
