# Supplementary material for: mlh3 mutations in baker’s yeast alter meiotic recombination outcomes by increasing noncrossover events genome-wide
Source: PLoS Genet. 2017 Aug 21;13(8):e1006974. doi: 10.1371/journal.pgen.1006974 (PMC5578695; doi:10.1371/journal.pgen.1006974)
Supplement: S1 File — S288c and YJM789 SNPs are shown in red and blue, respectively. (PDF) [file pgen.1006974.s001.pdf]

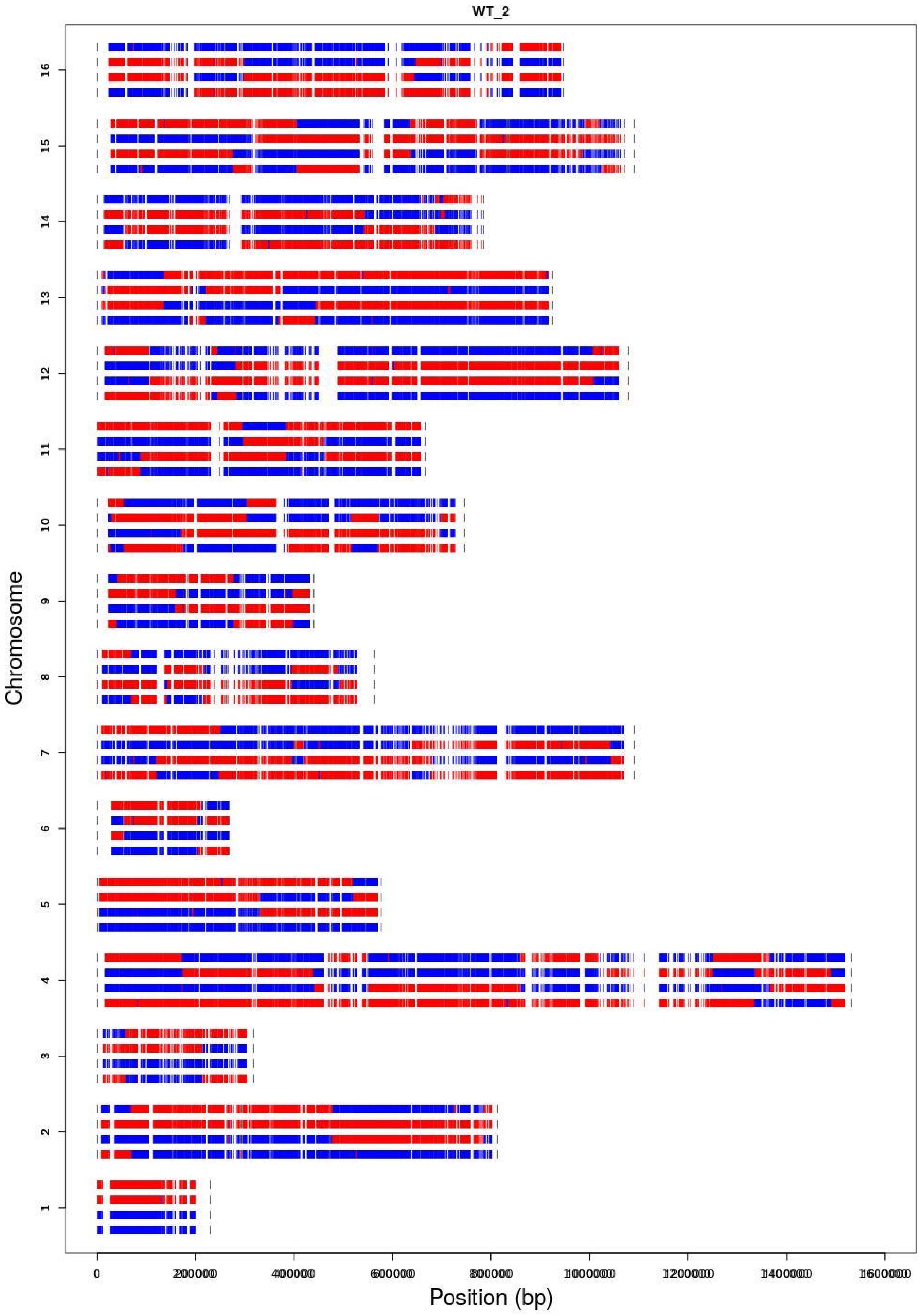
WT_2
Chromosome
16
15
14
13
12
11
10
9
8
7
6
5
4
3
2
1
Position (bp)
0
200000
400000
600000
800000
1000000
1200000
1400000
1600000

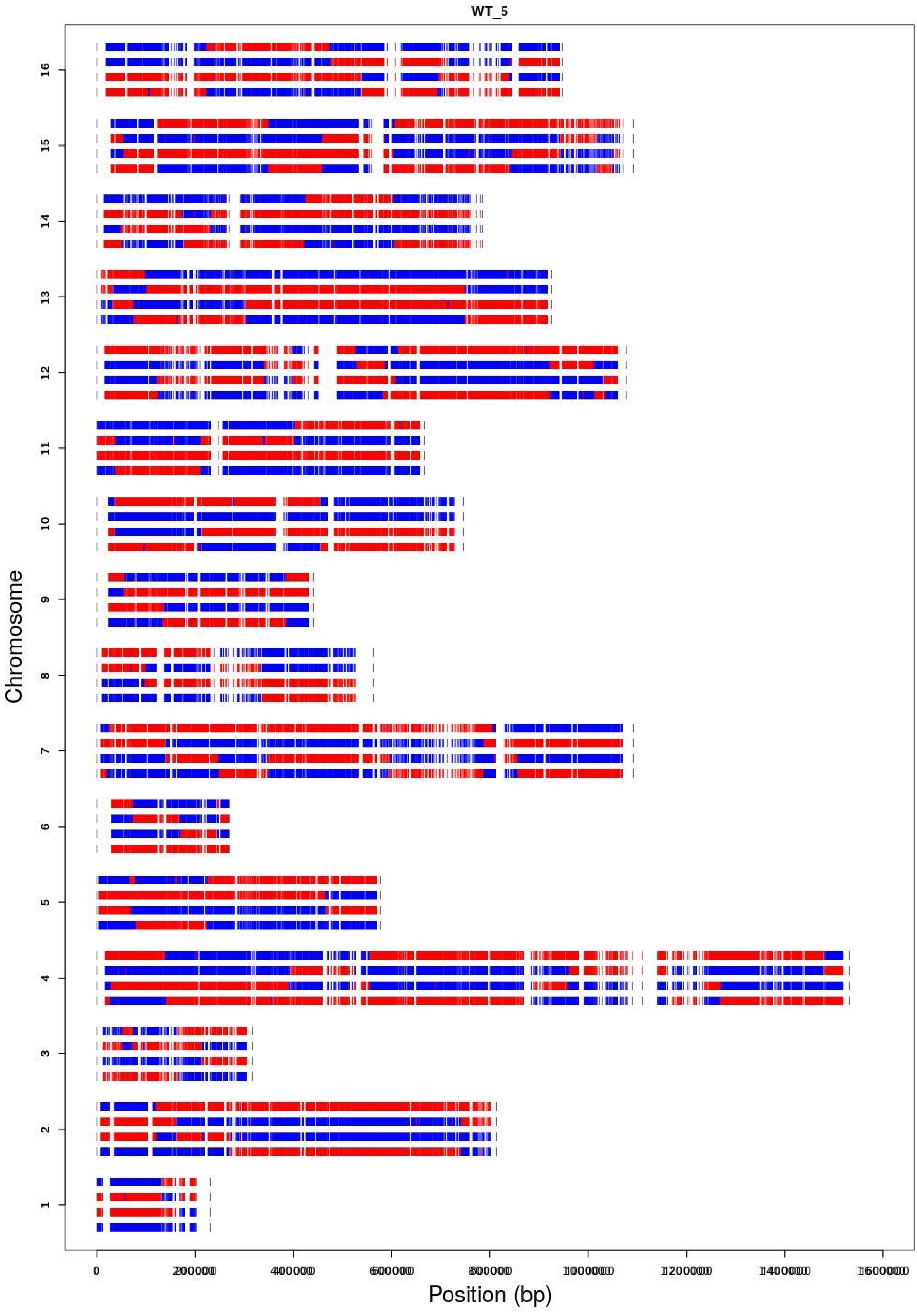
WT_5
Chromosome
16
15
14
13
12
11
10
9
8
7
6
5
4
3
2
1
0
200000
400000
600000
800000
1000000
1200000
1400000
1600000
Position (bp)

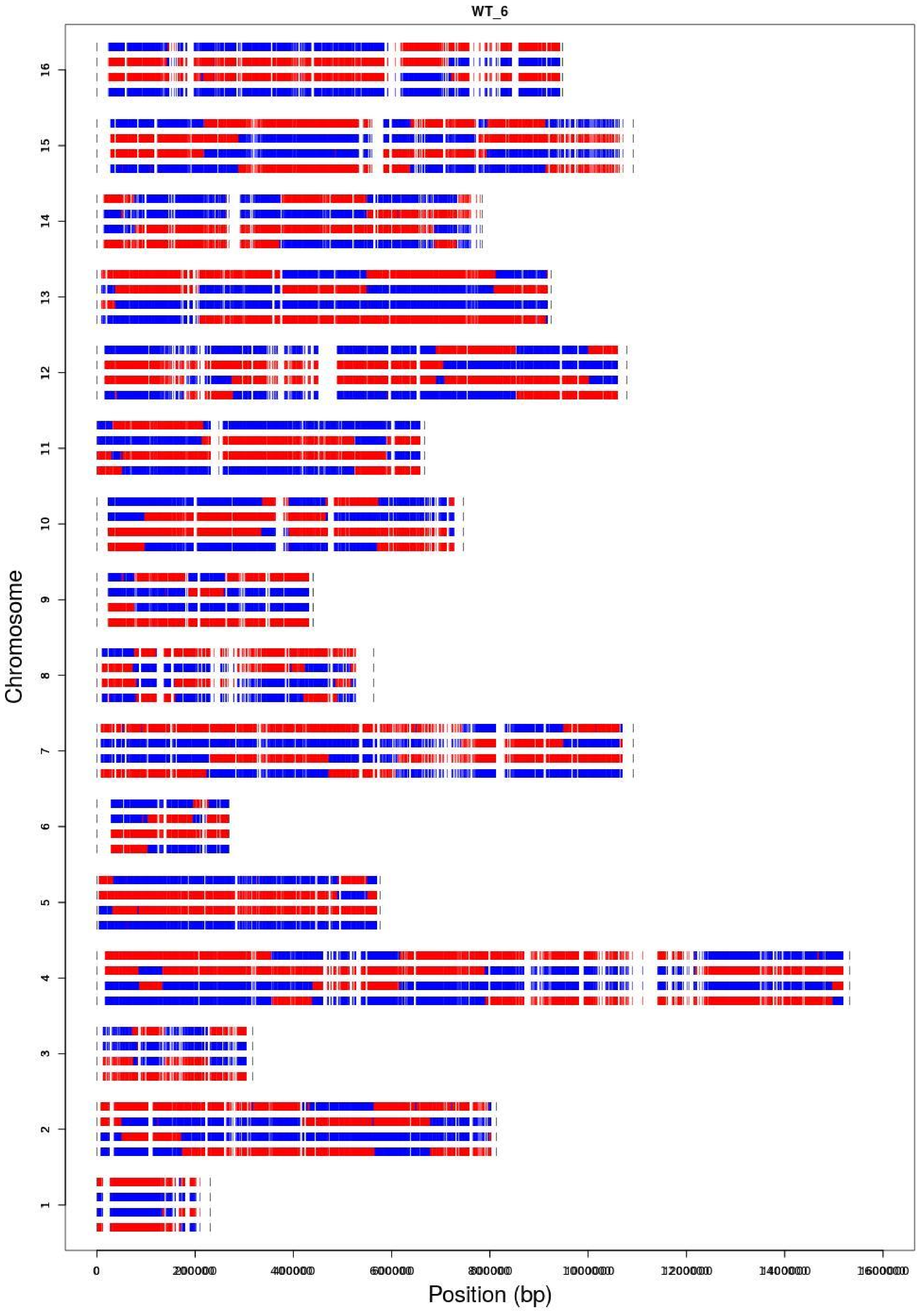

WT_6
Chromosome
16
15
14
13
12
11
10
9
8
7
6
5
4
3
2
1
0
200000
400000
600000
800000
1000000
1200000
1400000
1600000
Position (bp)

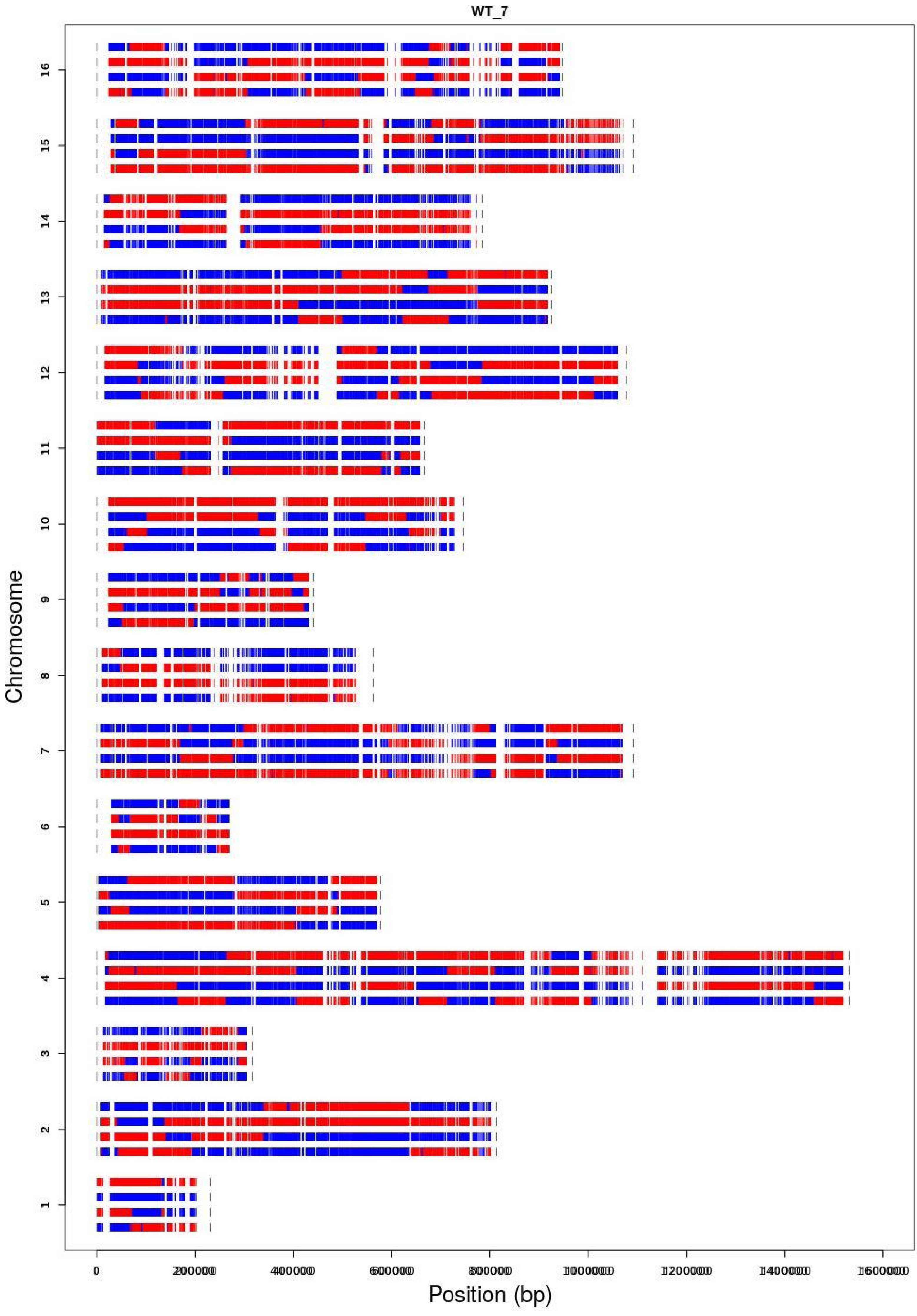

WT_7
Chromosome
16
15
14
13
12
11
10
9
8
7
6
5
4
3
2
1
0
200000
400000
600000
800000
1000000
1200000
1400000
1600000
Position (bp)

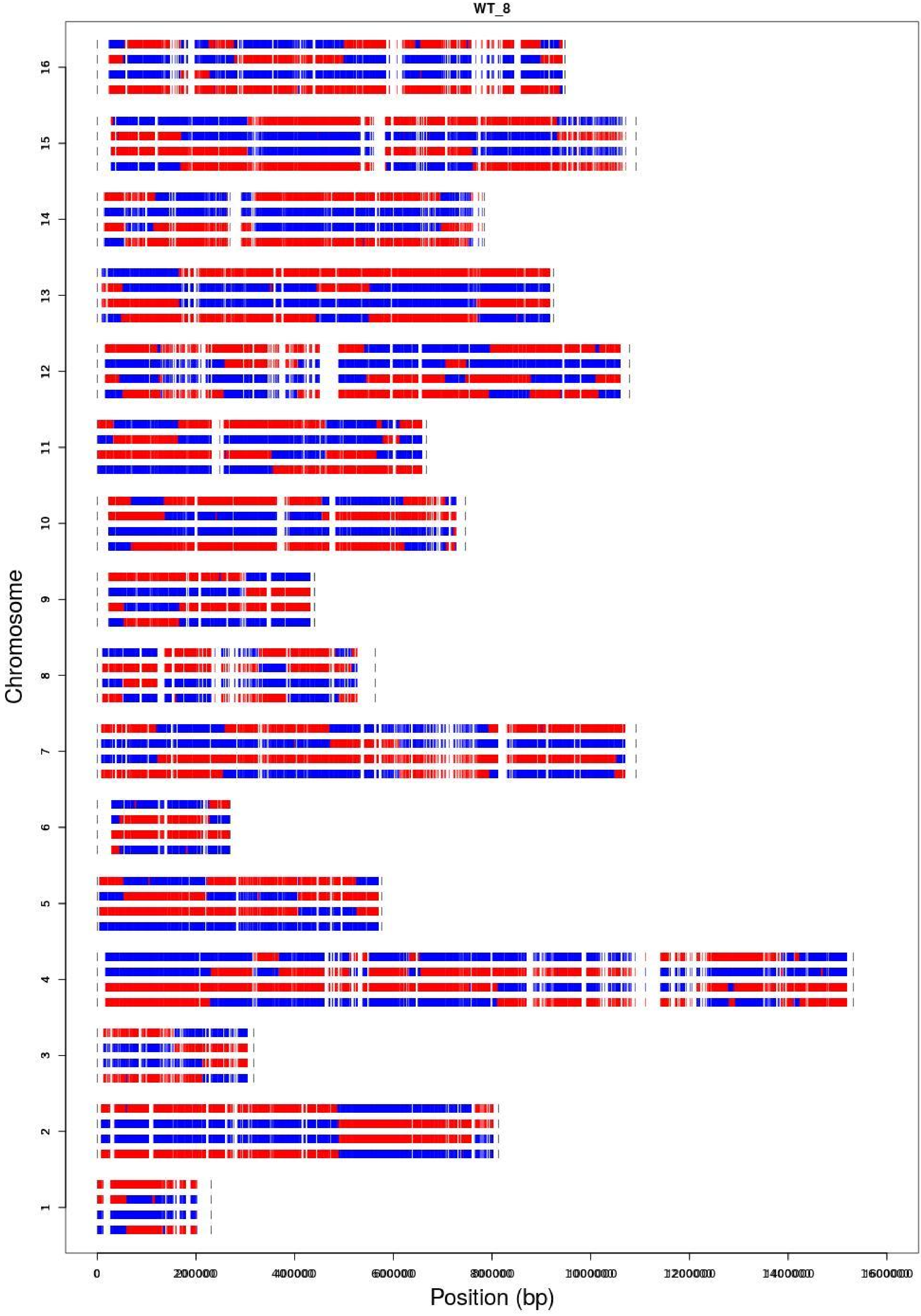
WT_8
Chromosome
16
15
14
13
12
11
10
9
8
7
6
5
4
3
2
1
0
200000
400000
600000
800000
1000000
1200000
1400000
1600000
Position (bp)

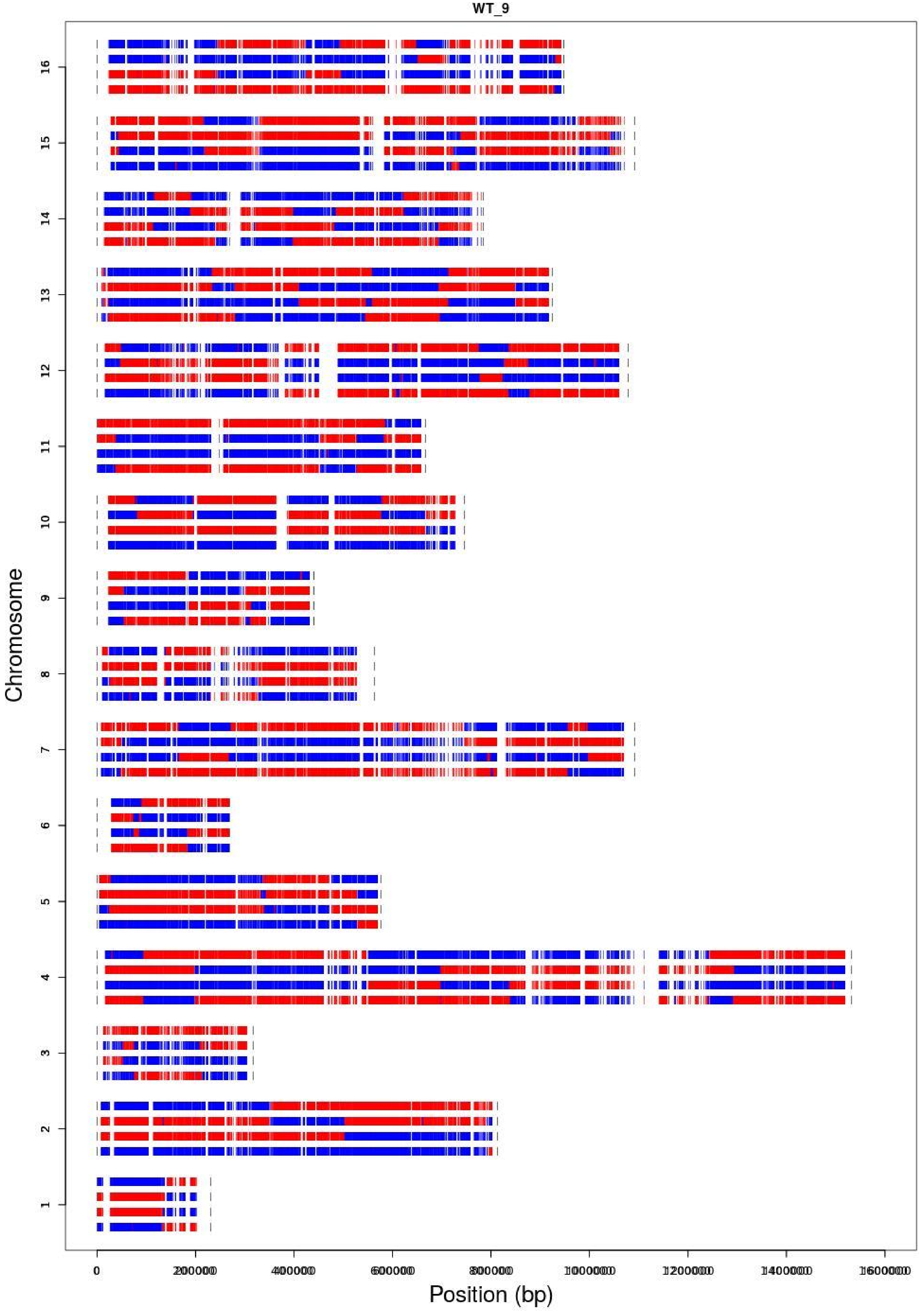

WT_9
Chromosome
16
15
14
13
12
11
10
9
8
7
6
5
4
3
2
1
0
200000
400000
600000
800000
1000000
1200000
1400000
1600000
Position (bp)

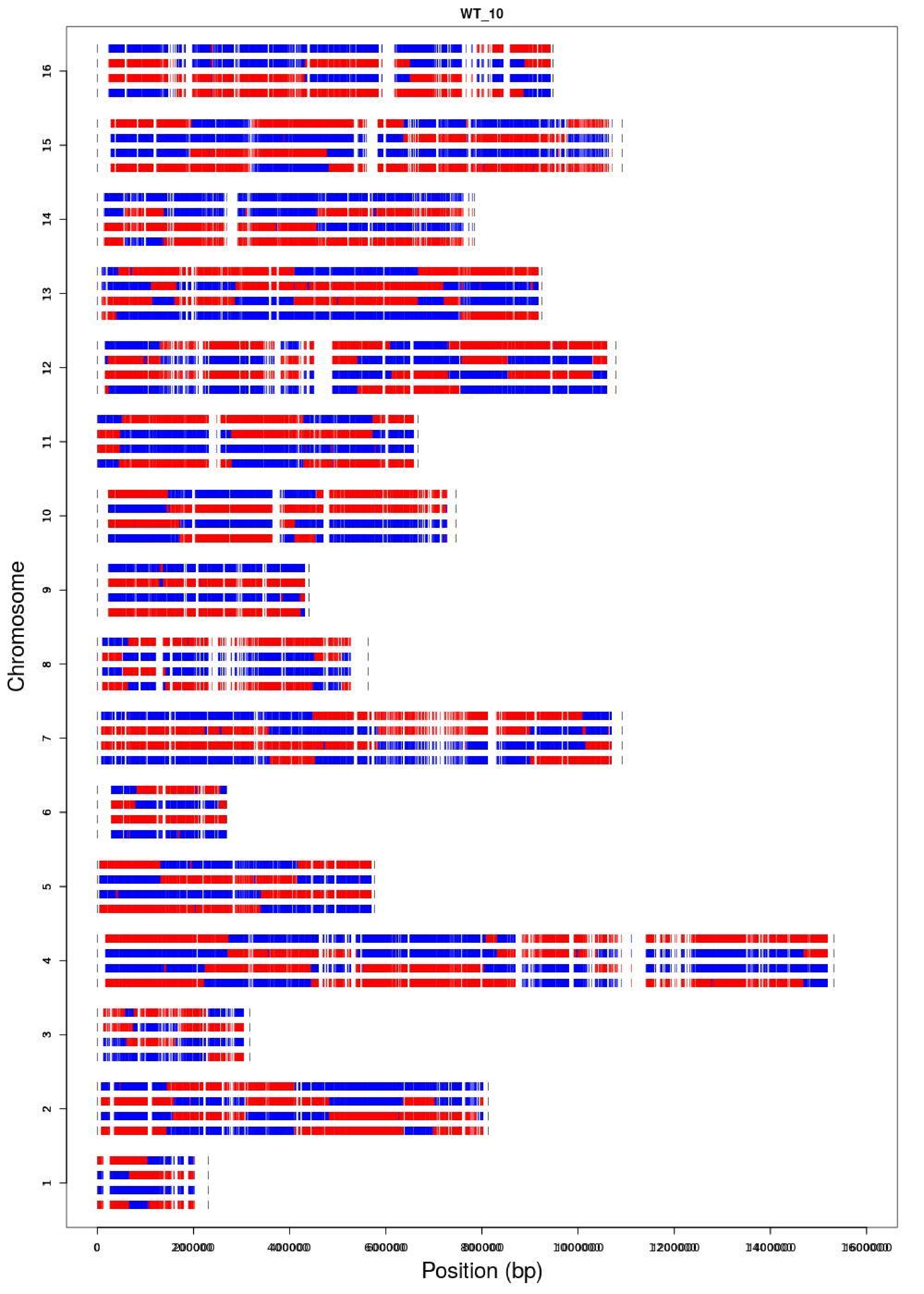

WT_10
Chromosome
16
15
14
13
12
11
10
9
8
7
6
5
4
3
2
1
0
200000
400000
600000
800000
1000000
1200000
1400000
1600000
Position (bp)

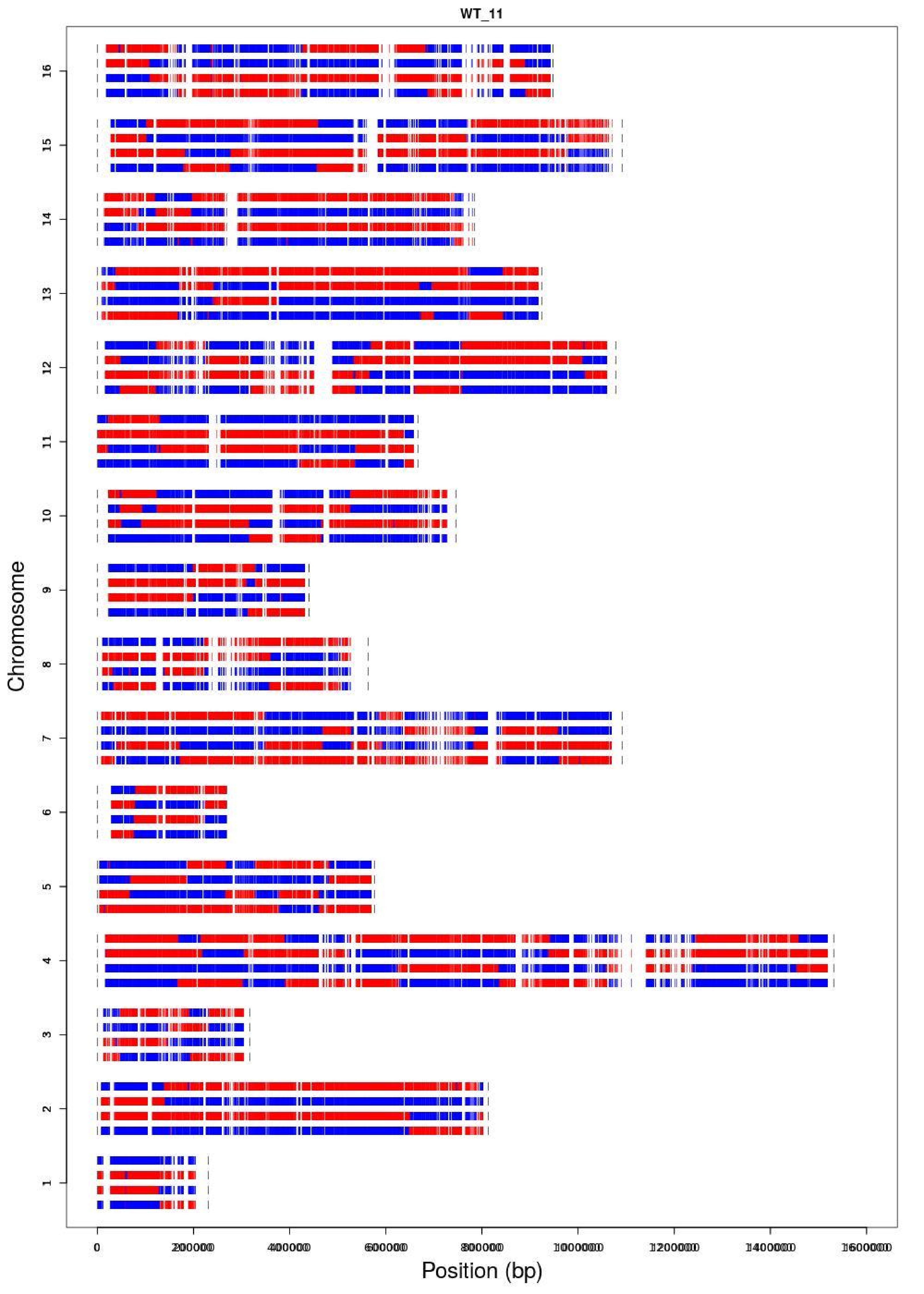
WT_11
Chromosome
16
15
14
13
12
11
10
9
8
7
6
5
4
3
2
1
0
200000
400000
600000
800000
1000000
1200000
1400000
1600000
Position (bp)

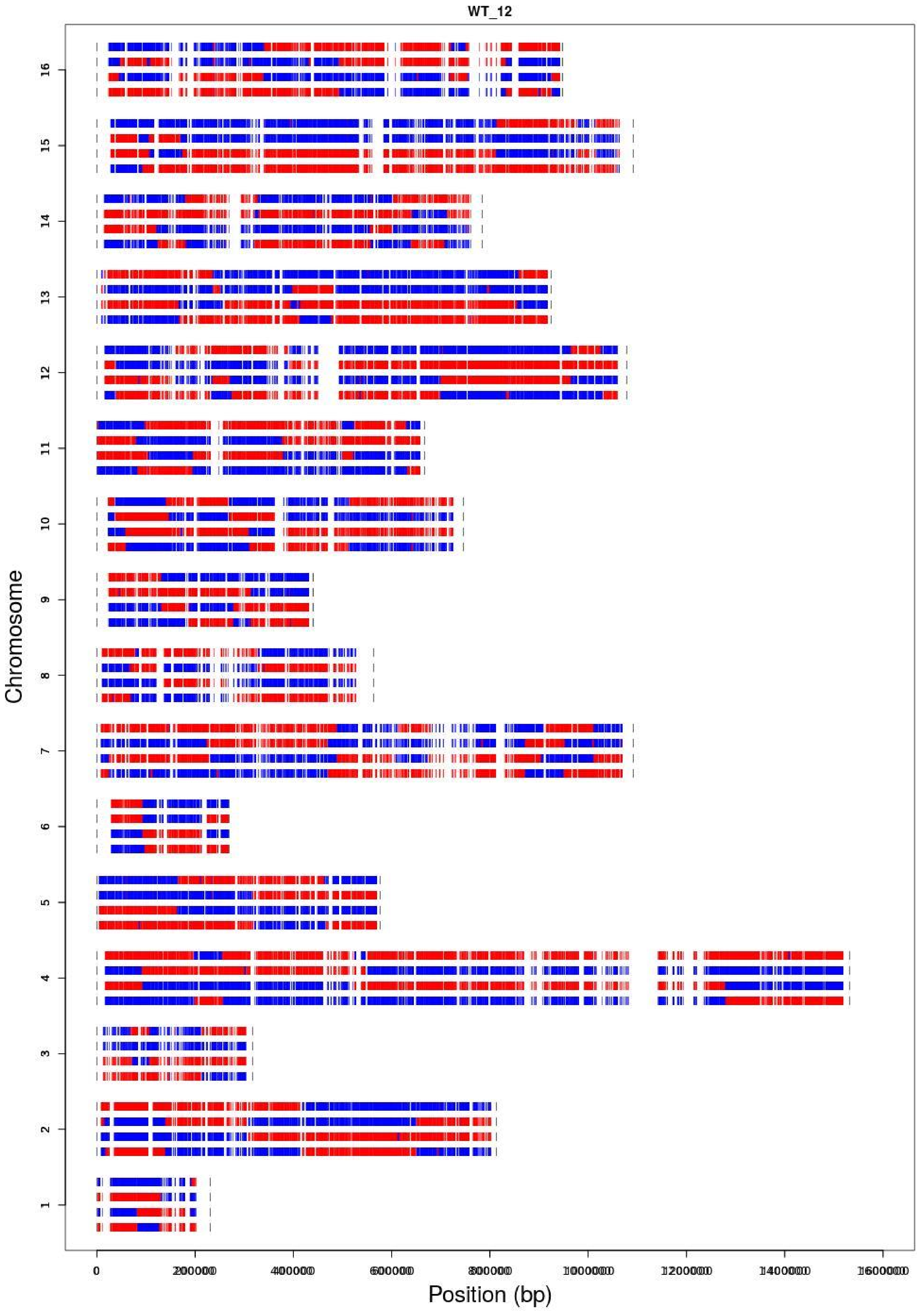

WT_12
Chromosome
Position (bp)
16
15
14
13
12
11
10
9
8
7
6
5
4
3
2
1
0
200000
400000
600000
800000
1000000
1200000
1400000
1600000

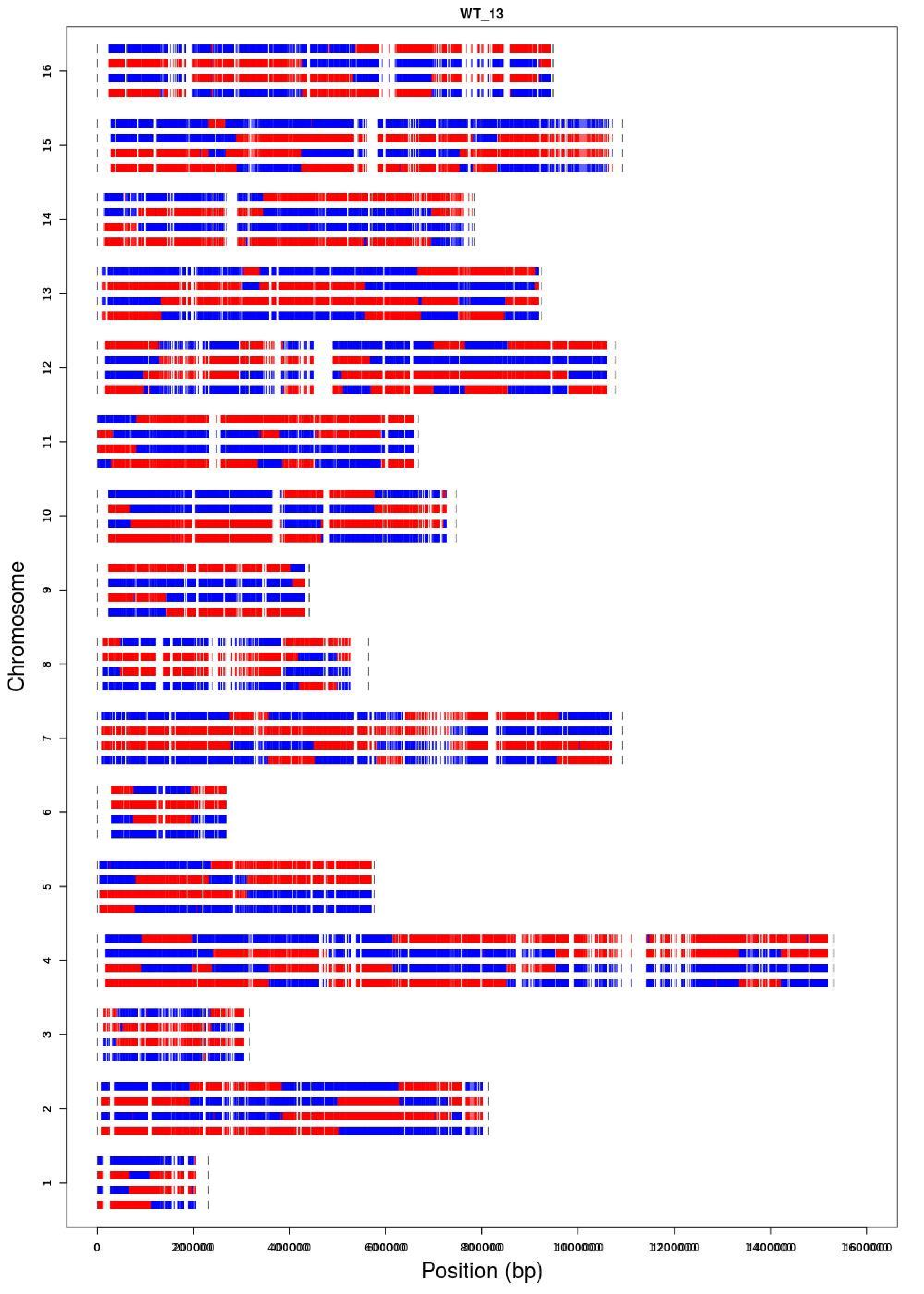

WT_13
Chromosome
16
15
14
13
12
11
10
9
8
7
6
5
4
3
2
1
0
200000
400000
600000
800000
1000000
1200000
1400000
1600000
Position (bp)

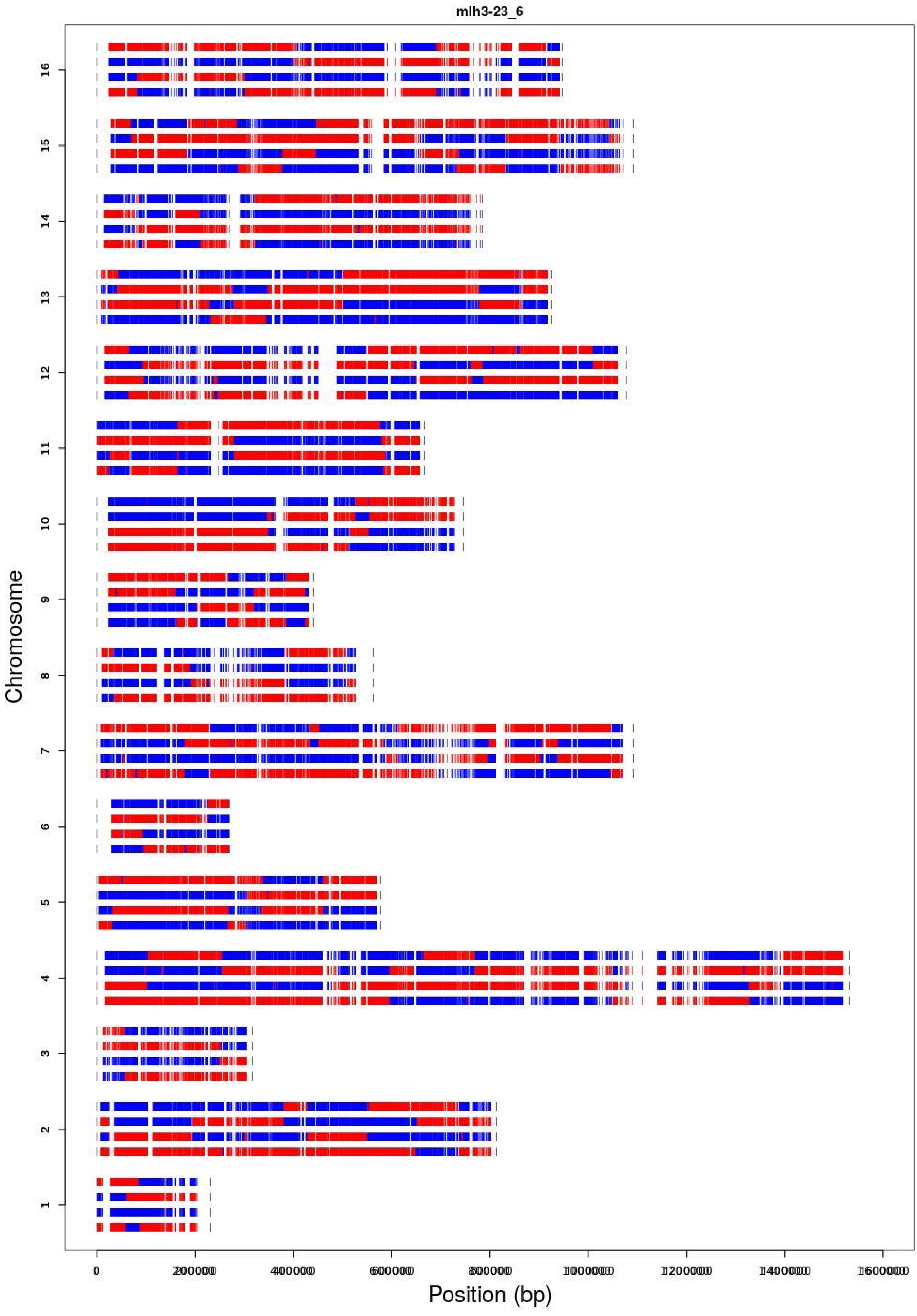

mlh3-23_6
Chromosome
16
15
14
13
12
11
10
9
8
7
6
5
4
3
2
1
0
200000
400000
600000
800000
1000000
1200000
1400000
1600000
Position (bp)

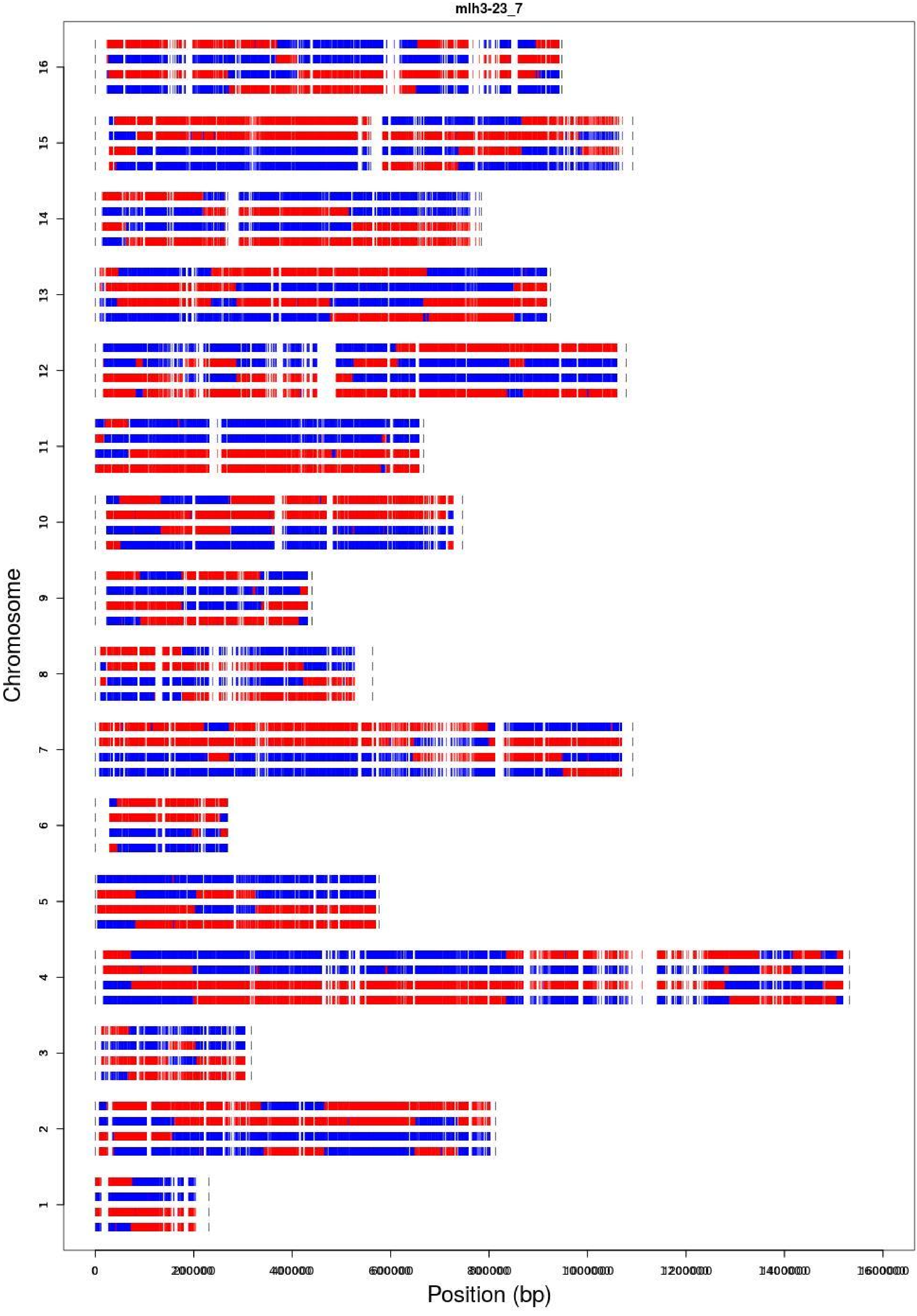

mlh3-23_7
Chromosome
16
15
14
13
12
11
10
9
8
7
6
5
4
3
2
1
0
200000
400000
600000
800000
1000000
1200000
1400000
1600000
Position (bp)

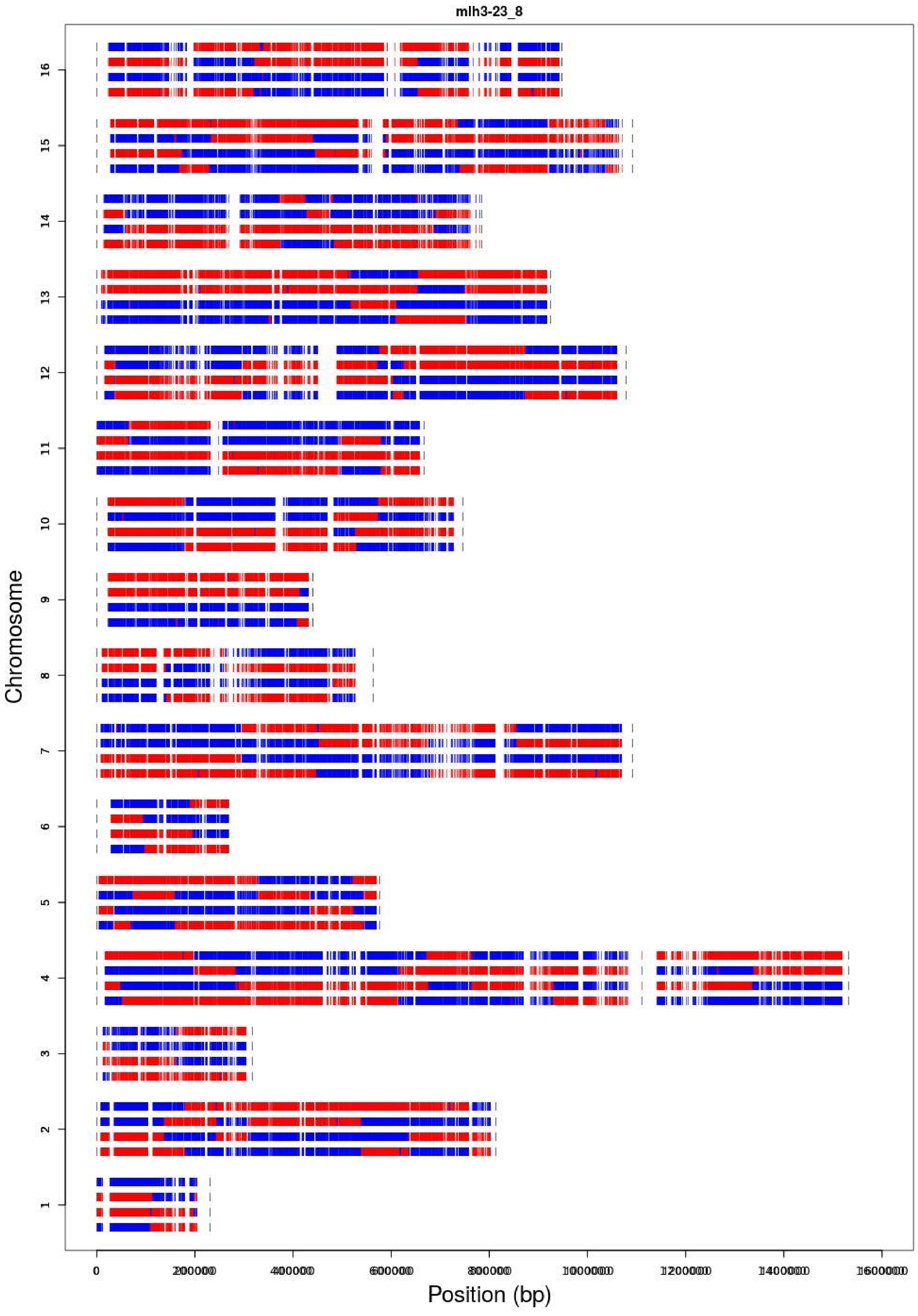
mlh3-23_8
Chromosome
16
15
14
13
12
11
10
9
8
7
6
5
4
3
2
1
0
200000
400000
600000
800000
1000000
1200000
1400000
1600000
Position (bp)

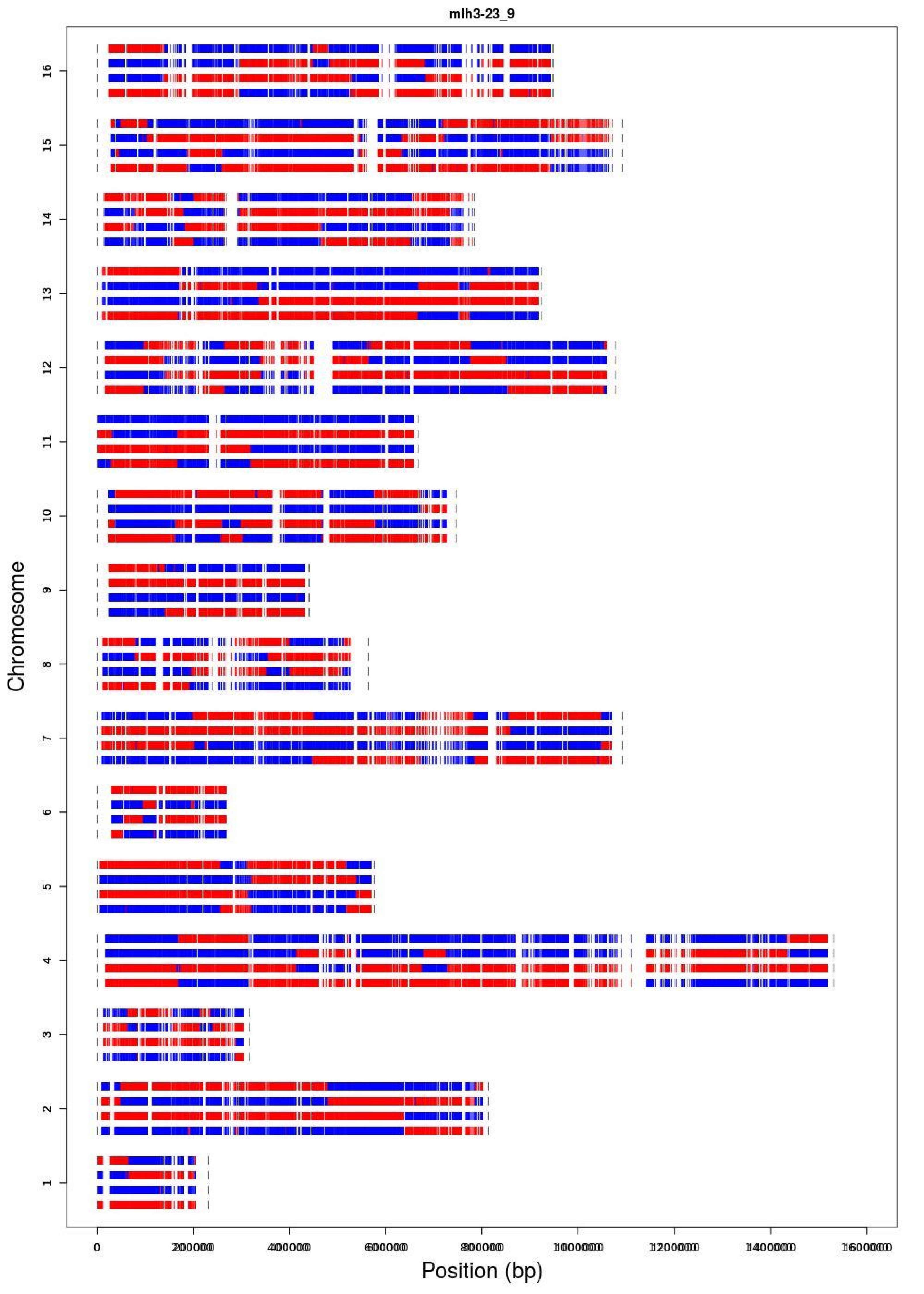
mlh3-23_9
Chromosome
1
2
3
4
5
6
7
8
9
10
11
12
13
14
15
16
Position (bp)
0
200000
400000
600000
800000
1000000
1200000
1400000
1600000

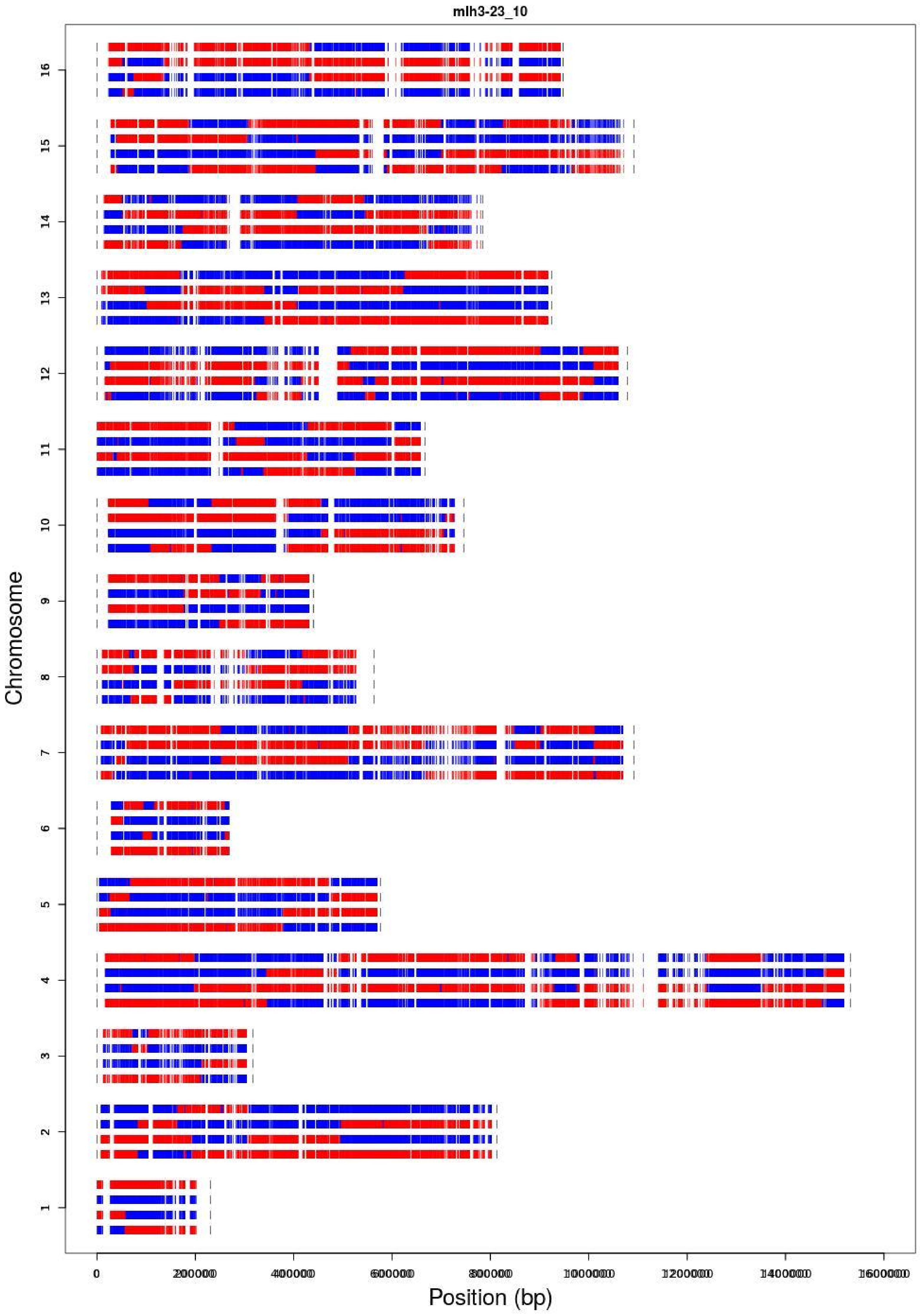

mlh3-23_10
Chromosome
16
15
14
13
12
11
10
9
8
7
6
5
4
3
2
1
0
200000
400000
600000
800000
1000000
1200000
1400000
1600000
Position (bp)

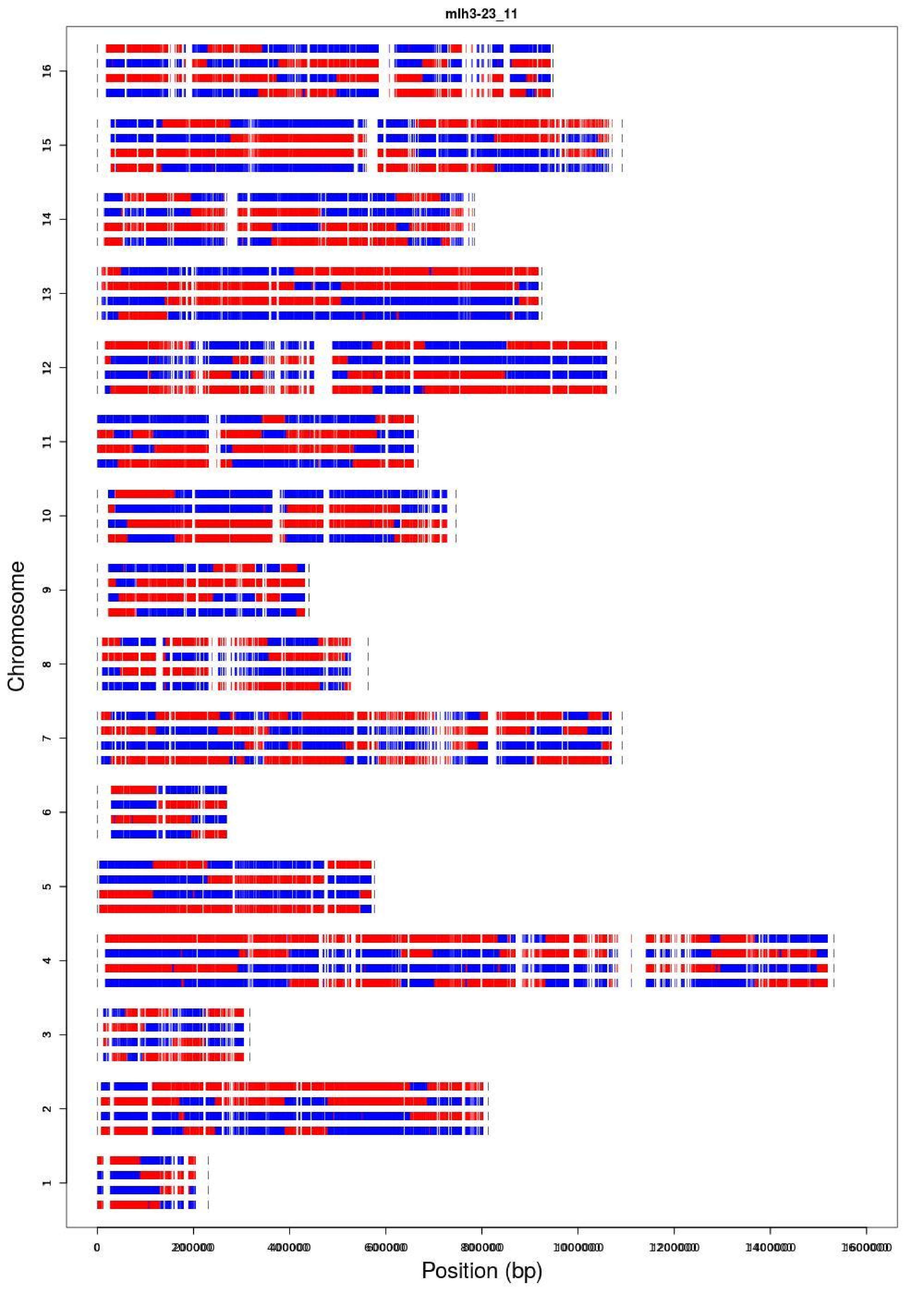
mlh3-23_11
Chromosome
16
15
14
13
12
11
10
9
8
7
6
5
4
3
2
1
0
200000
400000
600000
800000
1000000
1200000
1400000
1600000
Position (bp)

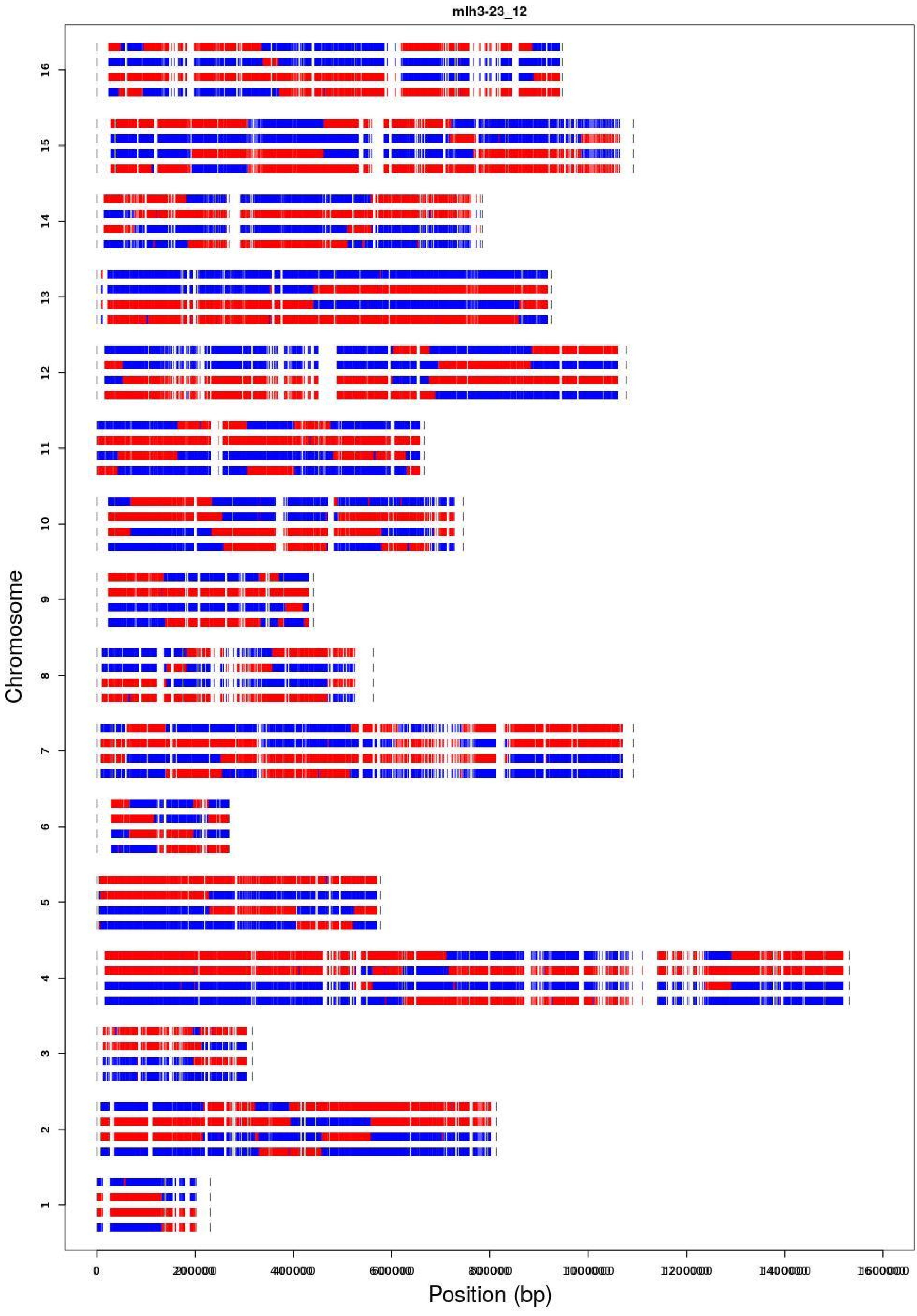

mlh3-23_12
Chromosome
16
15
14
13
12
11
10
9
8
7
6
5
4
3
2
1
0
200000
400000
600000
800000
1000000
1200000
1400000
1600000
Position (bp)

mlh3-32_6

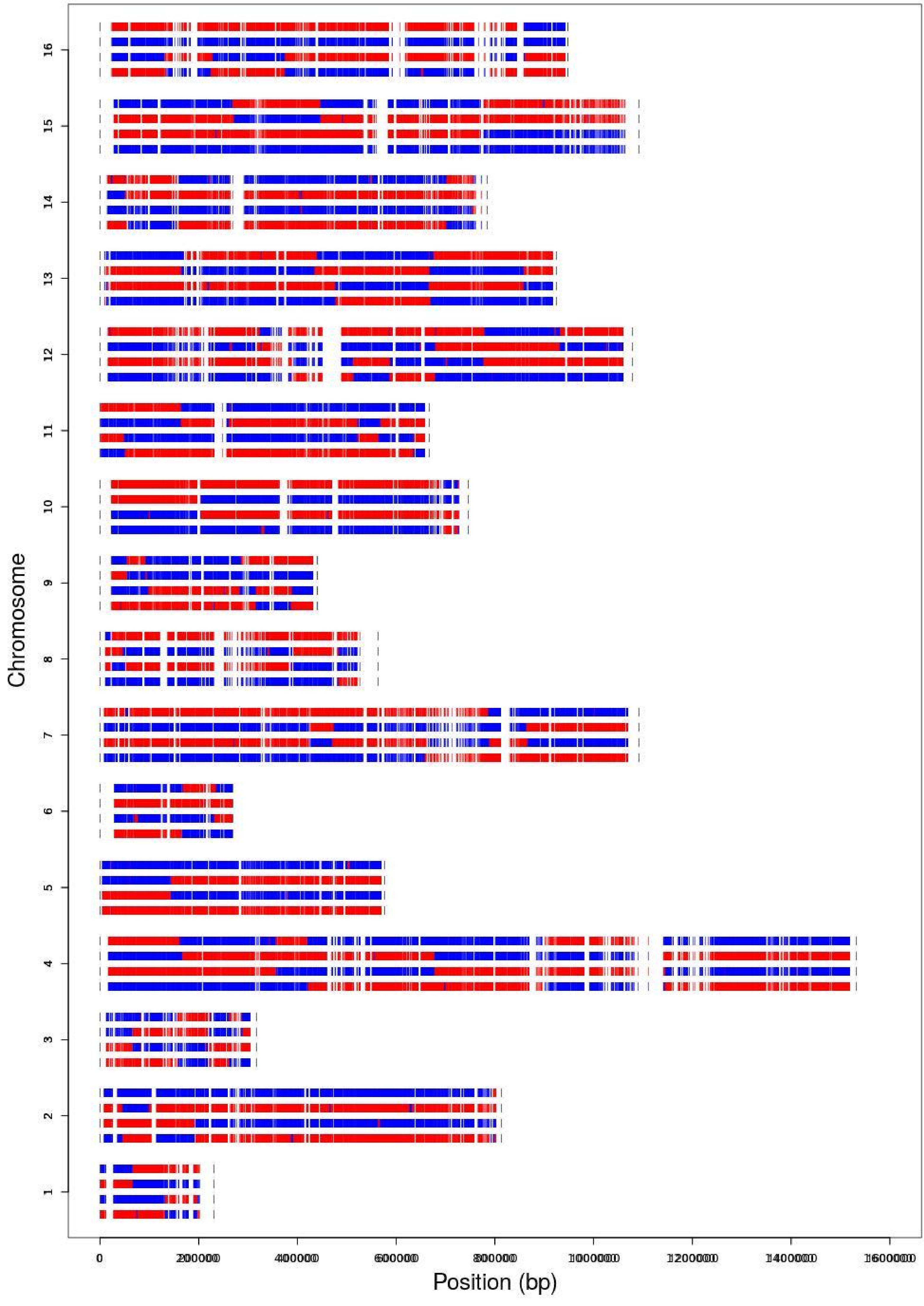
Chromosome
16
15
14
13
12
11
10
9
8
7
6
5
4
3
2
1
0
200000
400000
600000
800000
1000000
1200000
1400000
1600000
Position (bp)

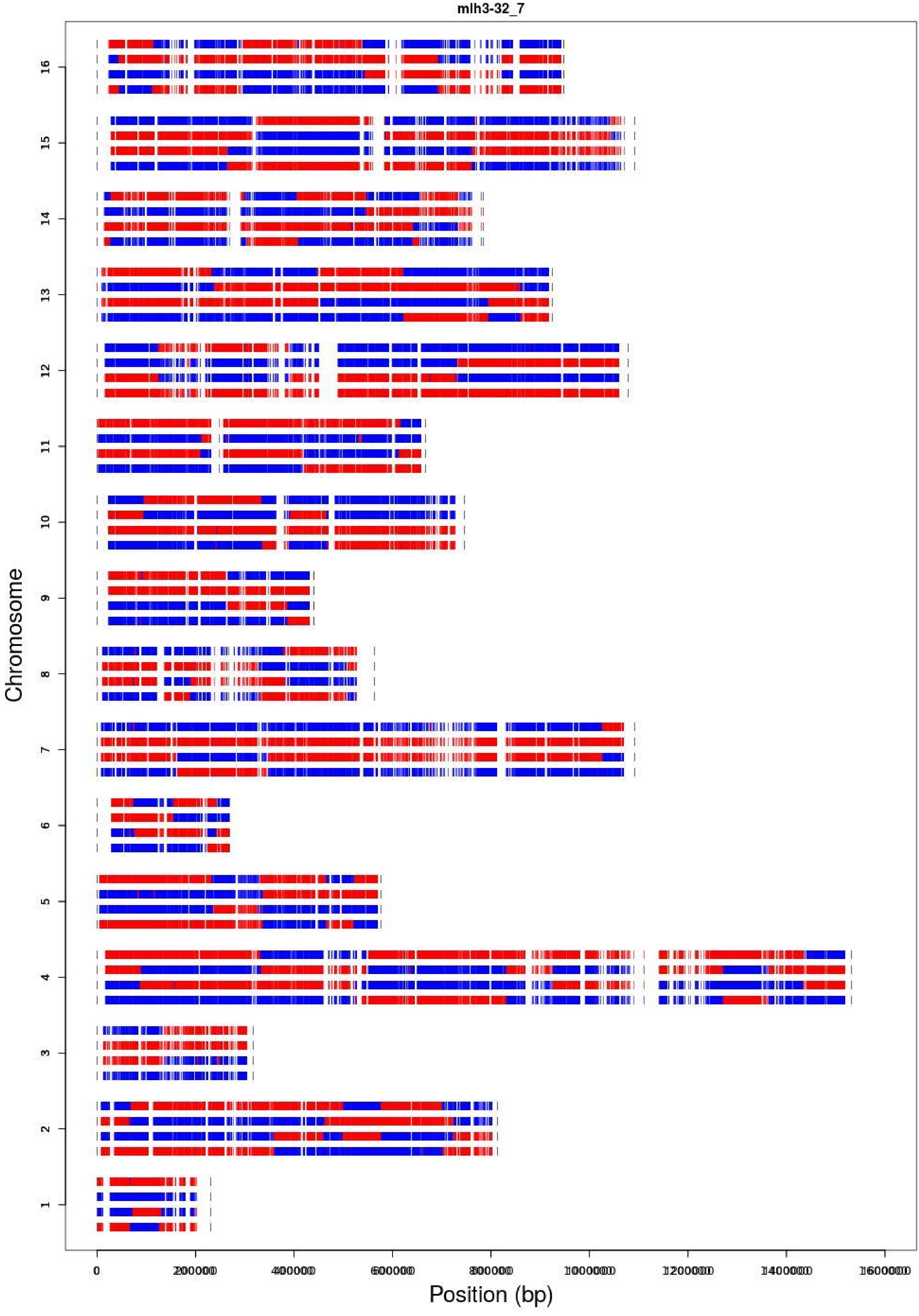
mlh3-32_7
Chromosome
16
15
14
13
12
11
10
9
8
7
6
5
4
3
2
1
0
200000
400000
600000
800000
1000000
1200000
1400000
1600000
Position (bp)

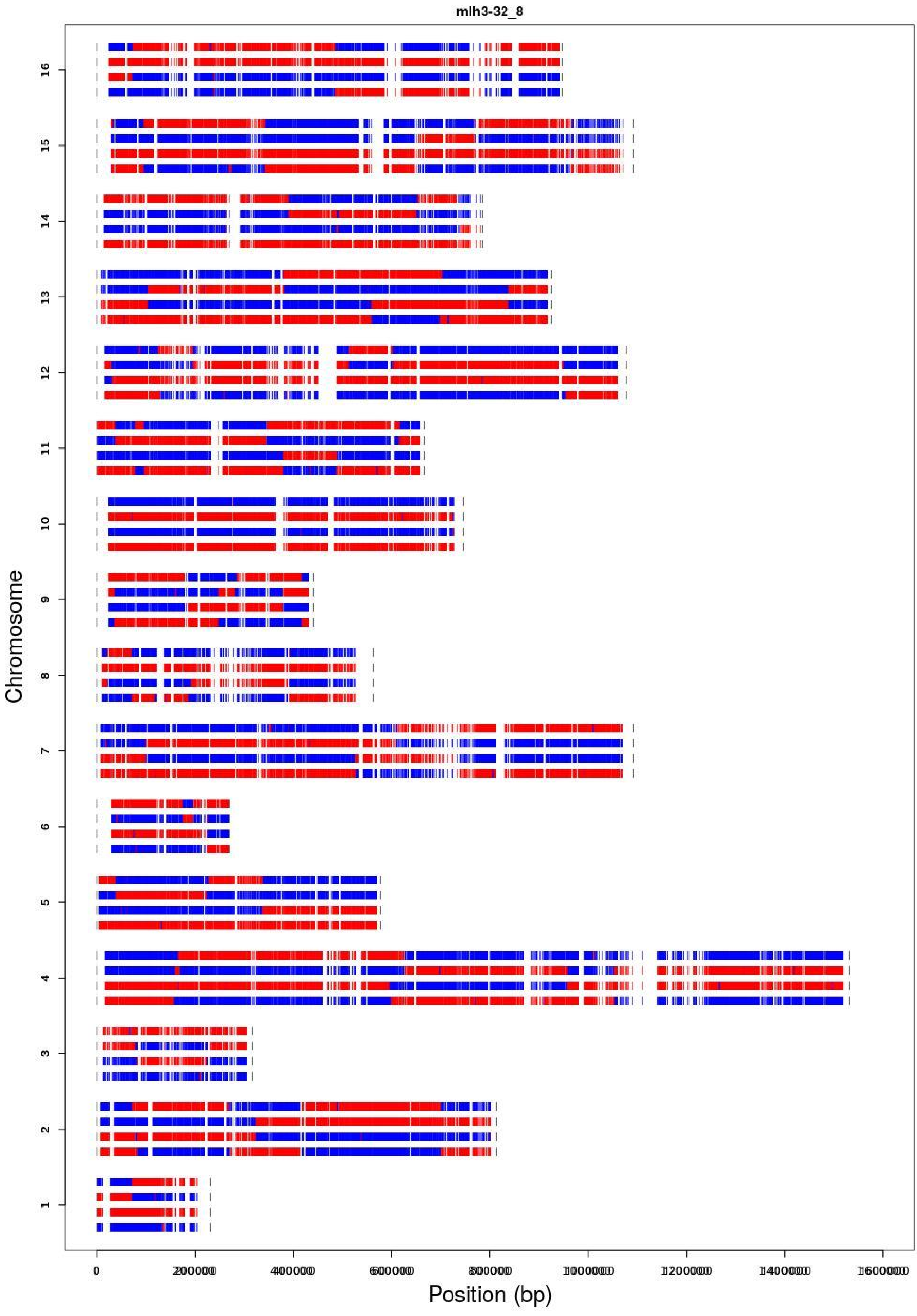

mlh3-32_8
Chromosome
16
15
14
13
12
11
10
9
8
7
6
5
4
3
2
1
0
200000
400000
600000
800000
1000000
1200000
1400000
1600000
Position (bp)

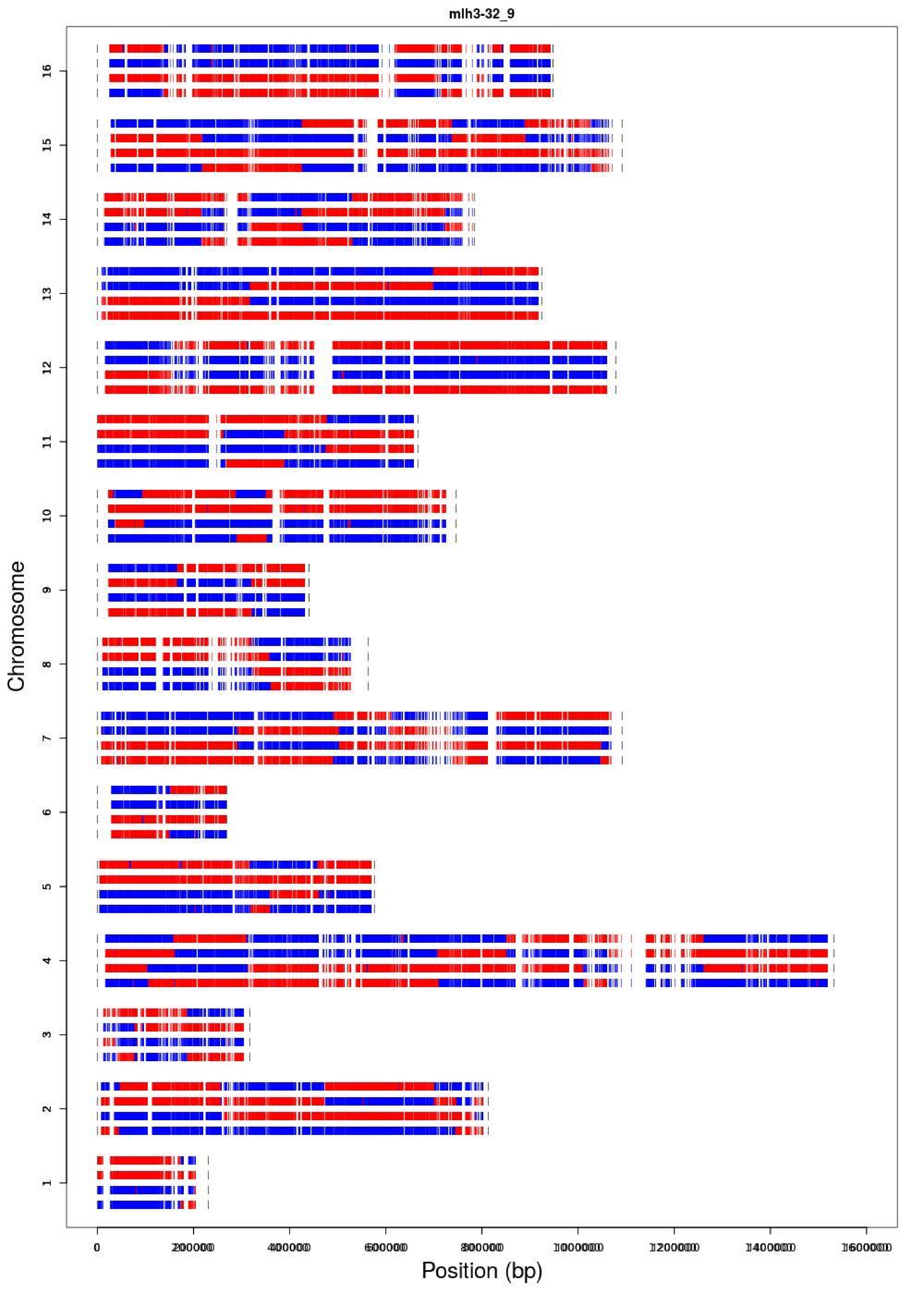

mlh3-32_9
Chromosome
16
15
14
13
12
11
10
9
8
7
6
5
4
3
2
1
0
200000
400000
600000
800000
1000000
1200000
1400000
1600000
Position (bp)

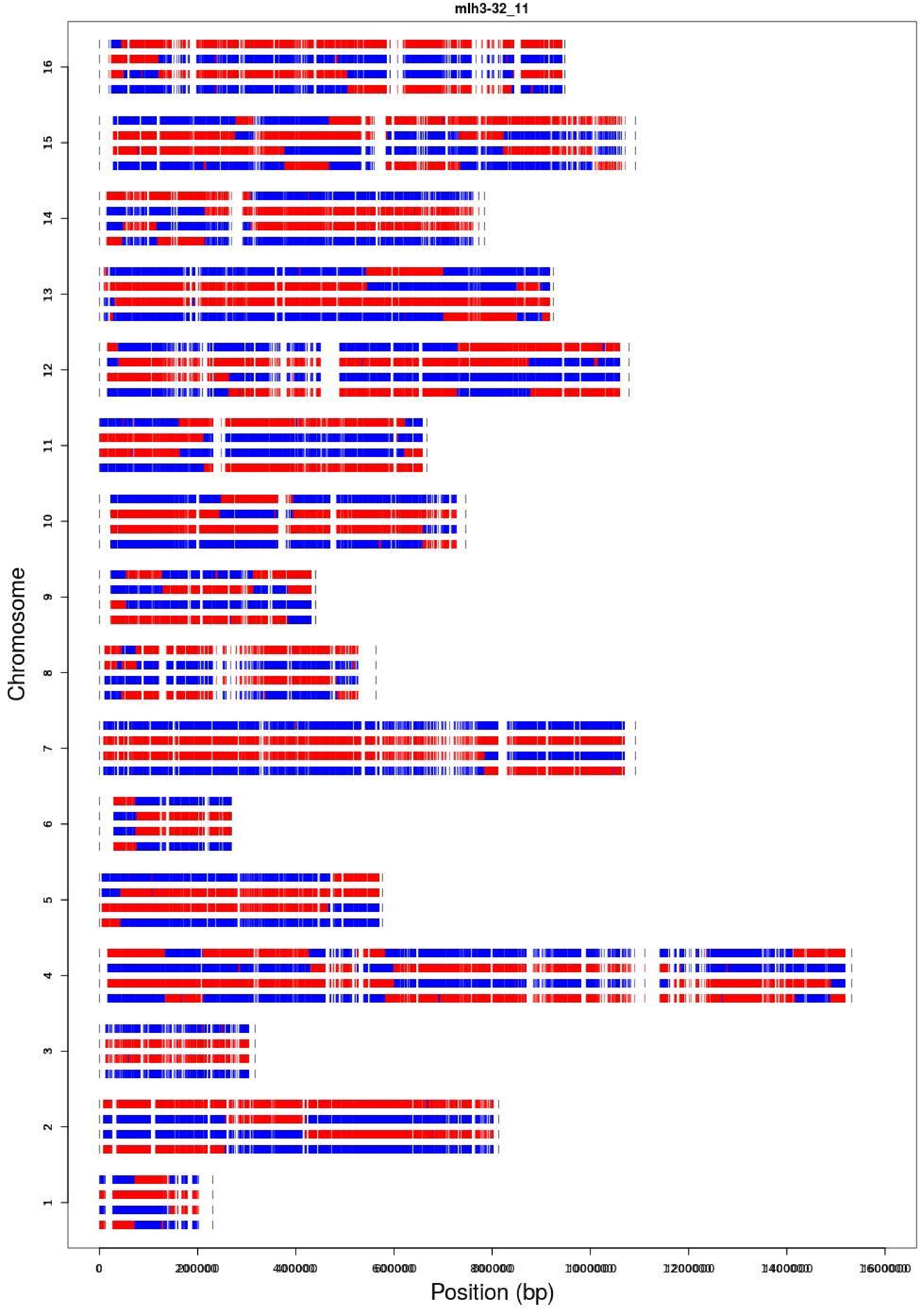

mlh3-32_11
Chromosome
Position (bp)
16
15
14
13
12
11
10
9
8
7
6
5
4
3
2
1
0
200000
400000
600000
800000
1000000
1200000
1400000
1600000

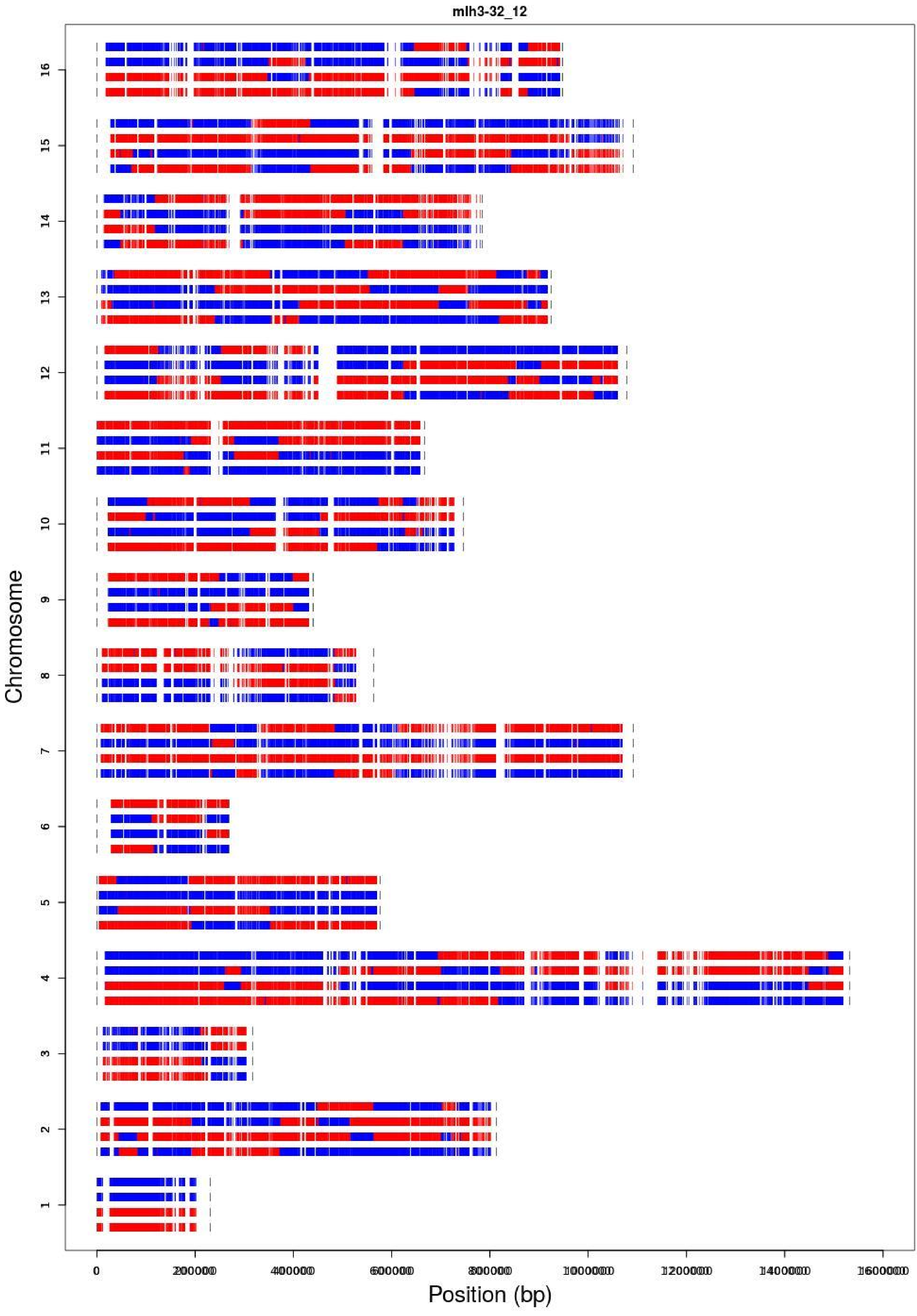
mlh3-32_12
Chromosome
16
15
14
13
12
11
10
9
8
7
6
5
4
3
2
1
0
200000
400000
600000
800000
1000000
1200000
1400000
1600000
Position (bp)

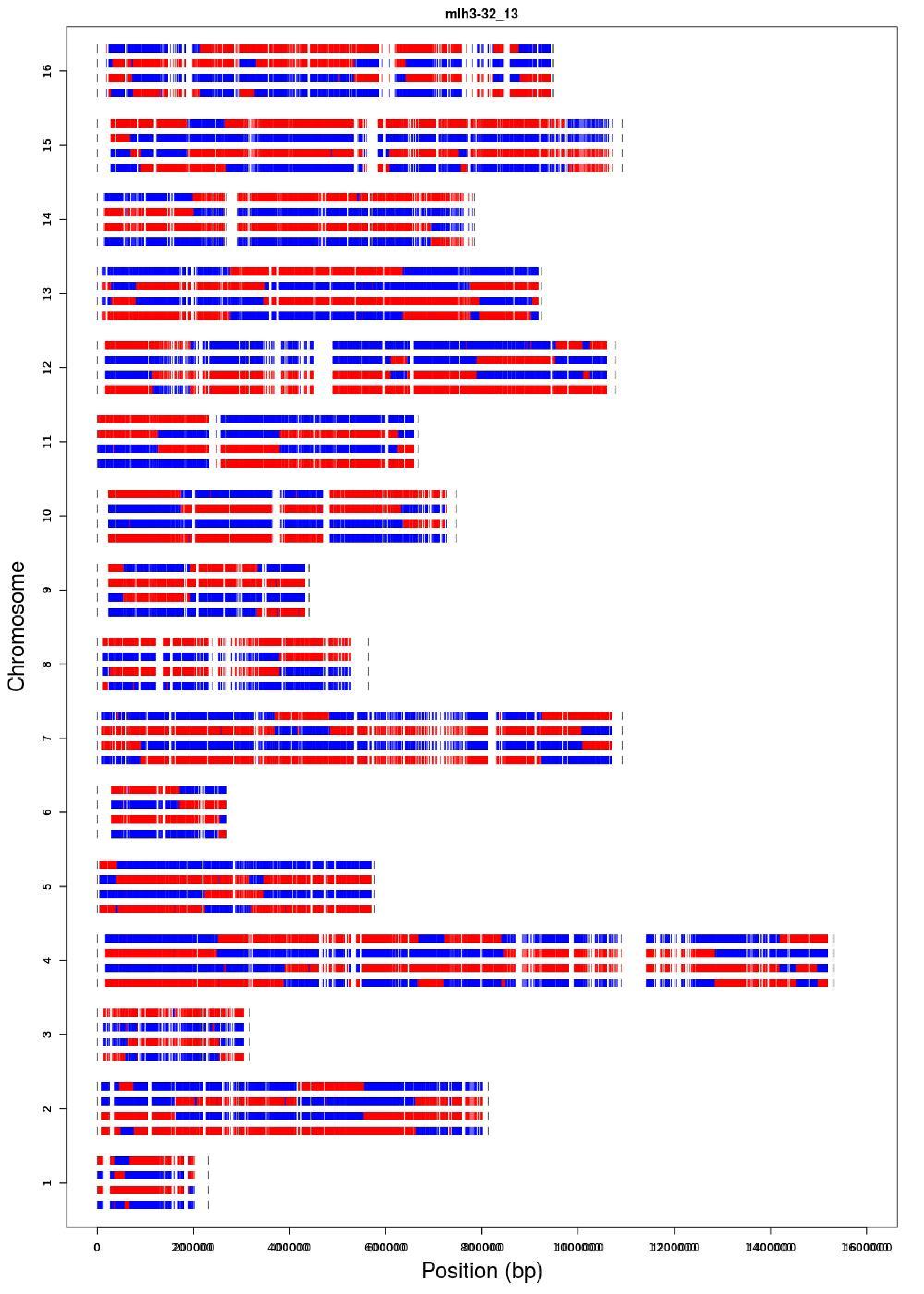
mlh3-32_13
Chromosome
16
15
14
13
12
11
10
9
8
7
6
5
4
3
2
1
0
200000
400000
600000
800000
1000000
1200000
1400000
1600000
Position (bp)

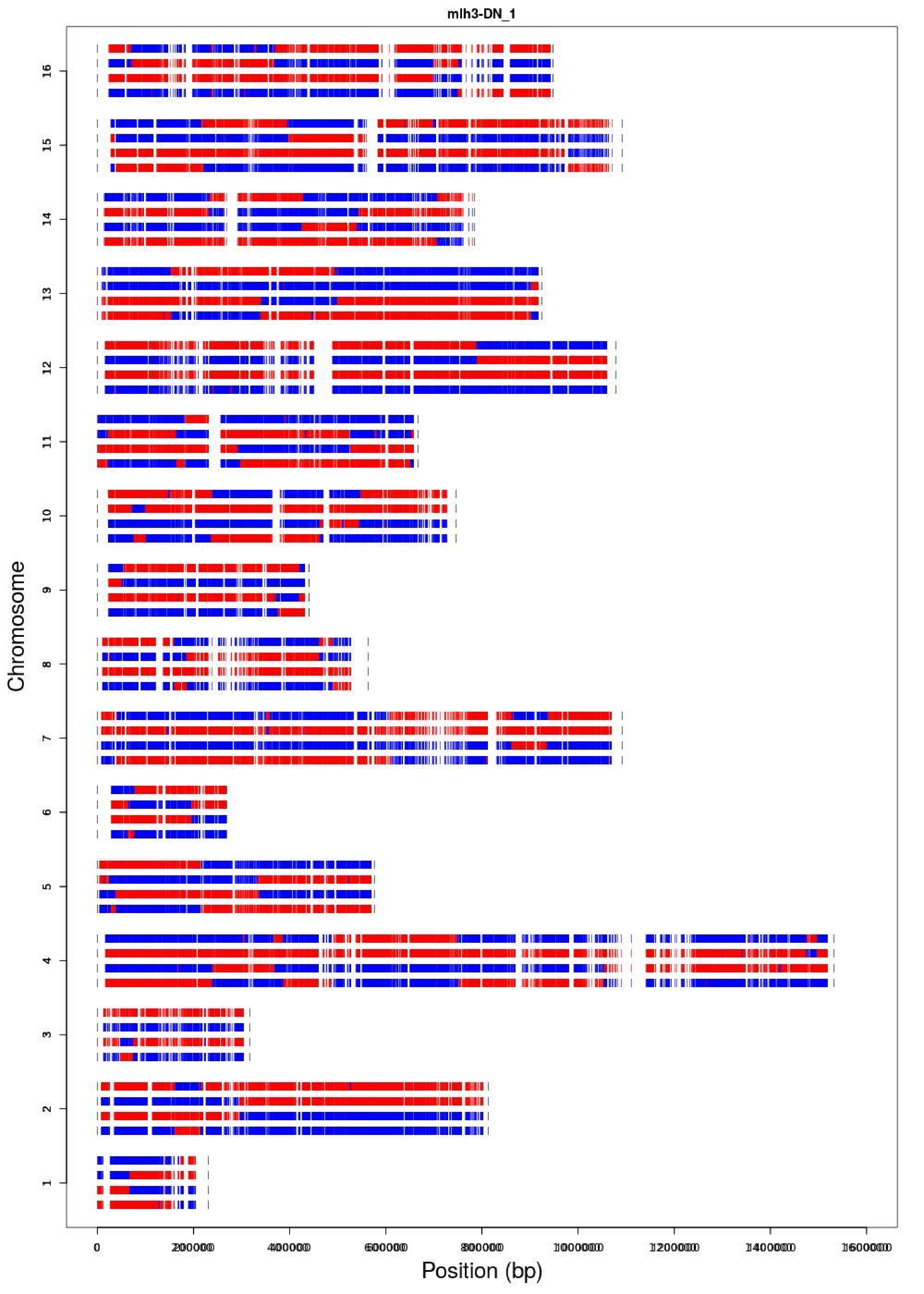
mlh3-DN_1
Chromosome
16
15
14
13
12
11
10
9
8
7
6
5
4
3
2
1
Position (bp)
0
200000
400000
600000
800000
1000000
1200000
1400000
1600000

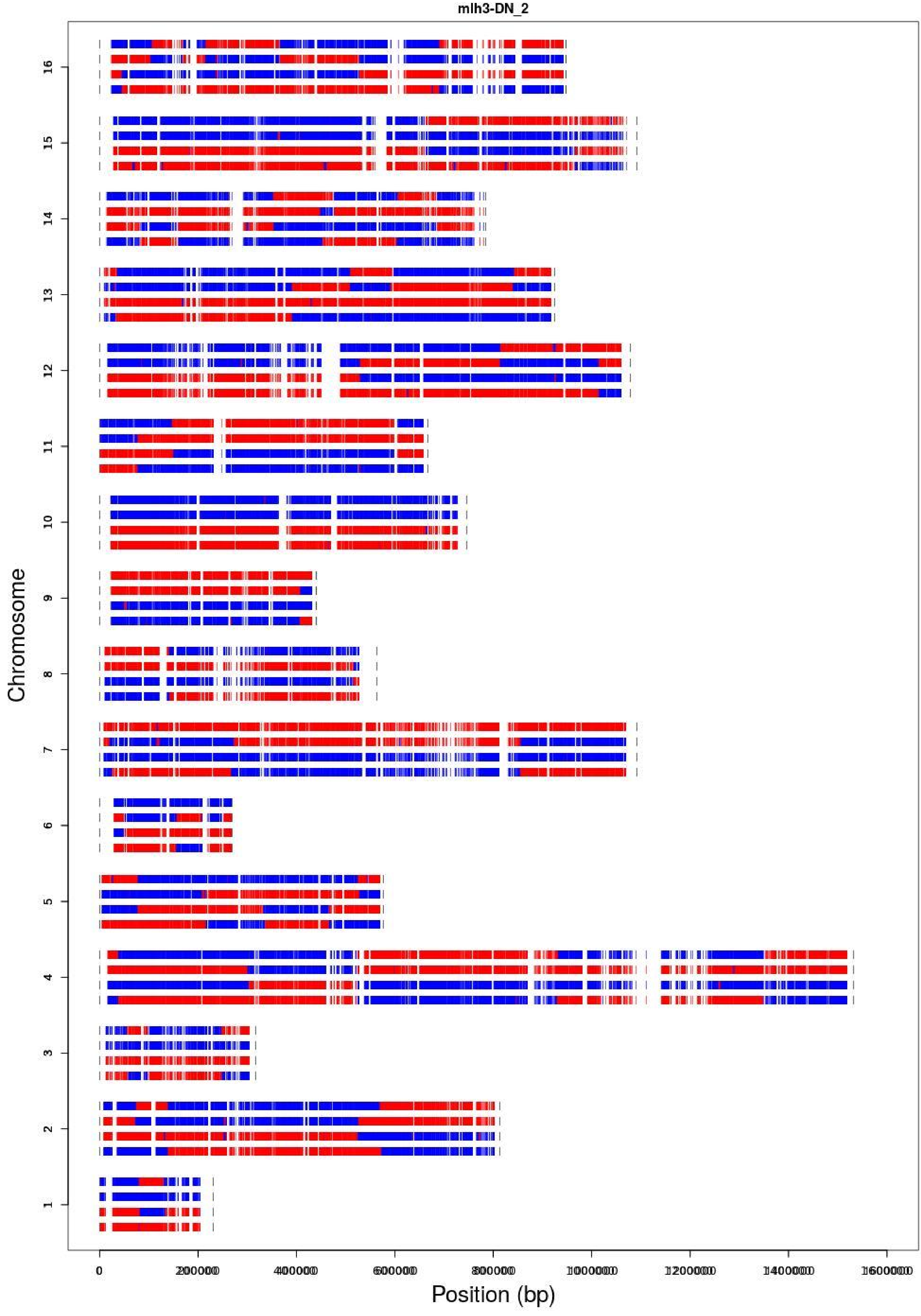

mlh3-DN_2
Chromosome
16
15
14
13
12
11
10
9
8
7
6
5
4
3
2
1
0
200000
400000
600000
800000
1000000
1200000
1400000
1600000
Position (bp)

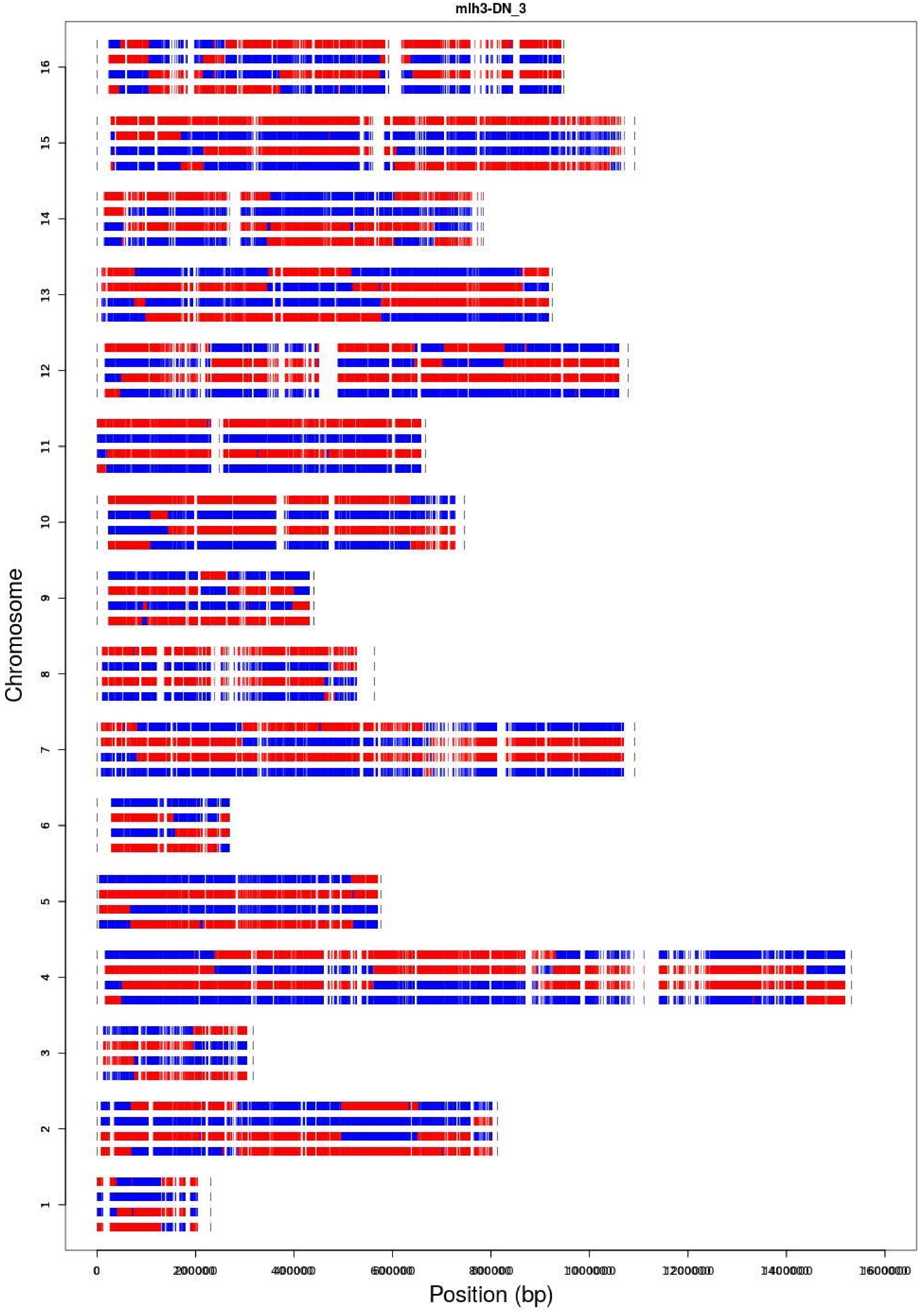
mlh3-DN_3
Chromosome
16
15
14
13
12
11
10
9
8
7
6
5
4
3
2
1
0
200000
400000
600000
800000
1000000
1200000
1400000
1600000
Position (bp)

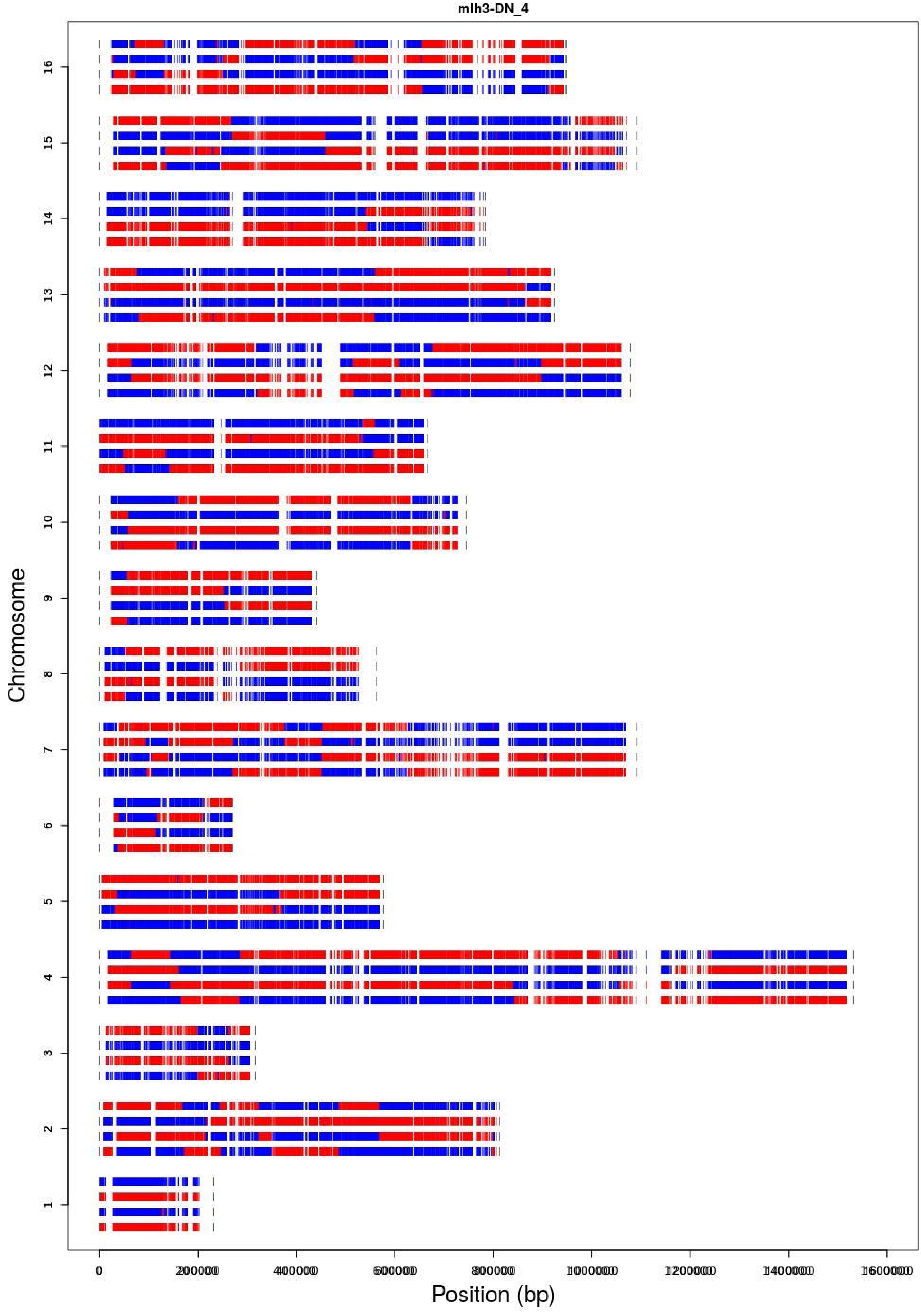

mlh3-DN_4
Chromosome
16
15
14
13
12
11
10
9
8
7
6
5
4
3
2
1
0
200000
400000
600000
800000
1000000
1200000
1400000
1600000
Position (bp)

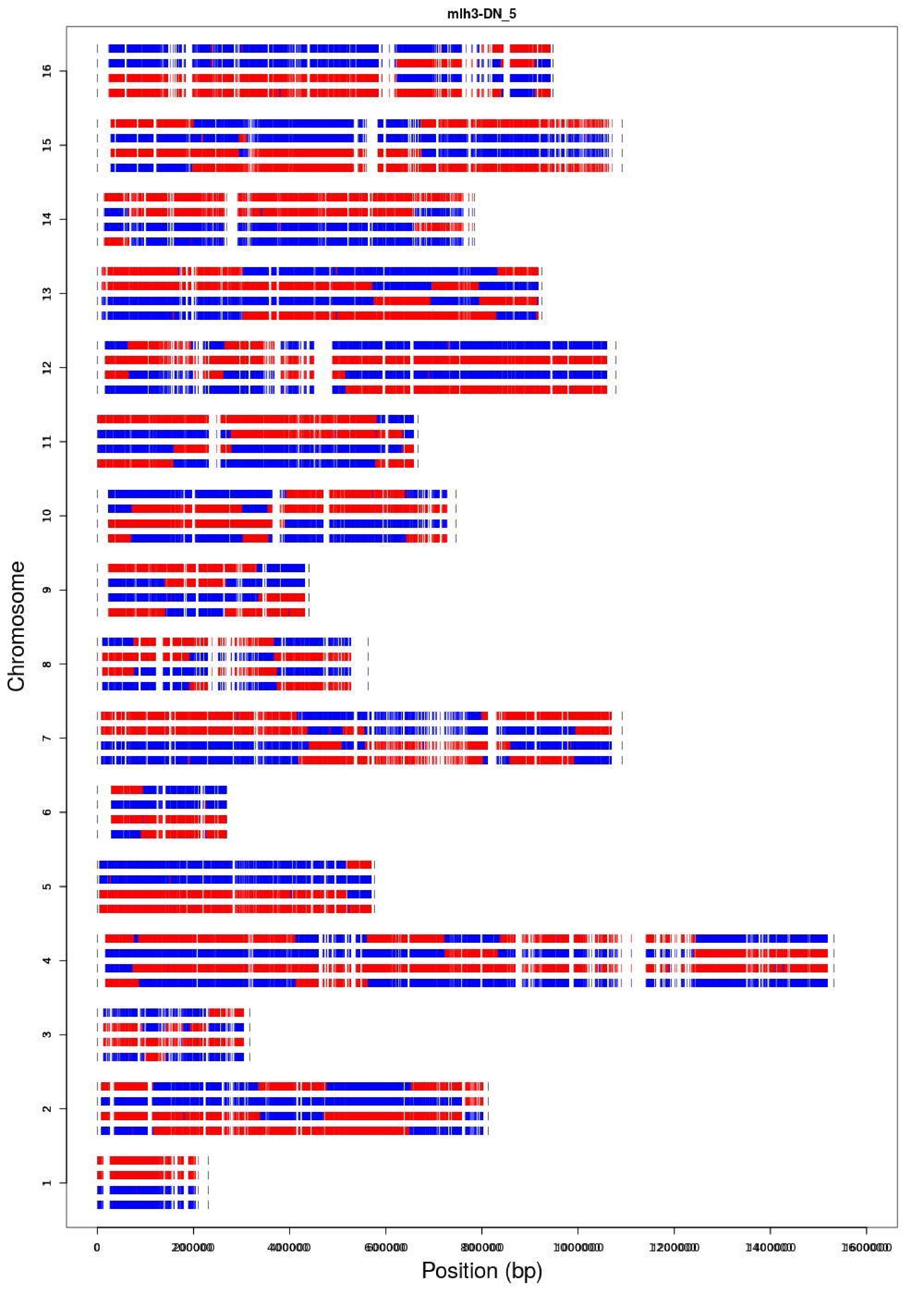
mlh3-DN_5
Chromosome
16
15
14
13
12
11
10
9
8
7
6
5
4
3
2
1
0
200000
400000
600000
800000
1000000
1200000
1400000
1600000
Position (bp)

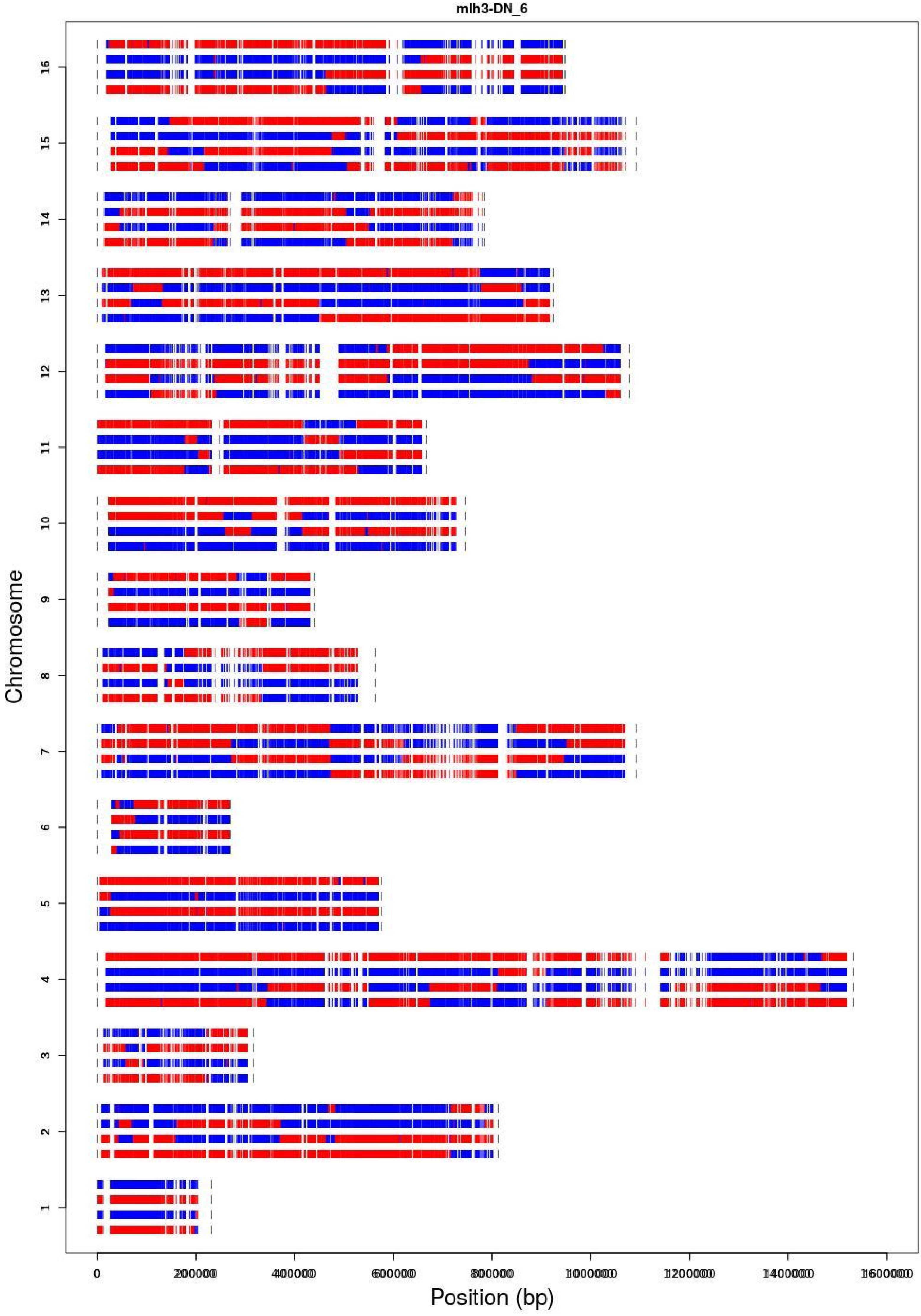

mlh3-DN_6
Chromosome
16
15
14
13
12
11
10
9
8
7
6
5
4
3
2
1
0
200000
400000
600000
800000
1000000
1200000
1400000
1600000
Position (bp)

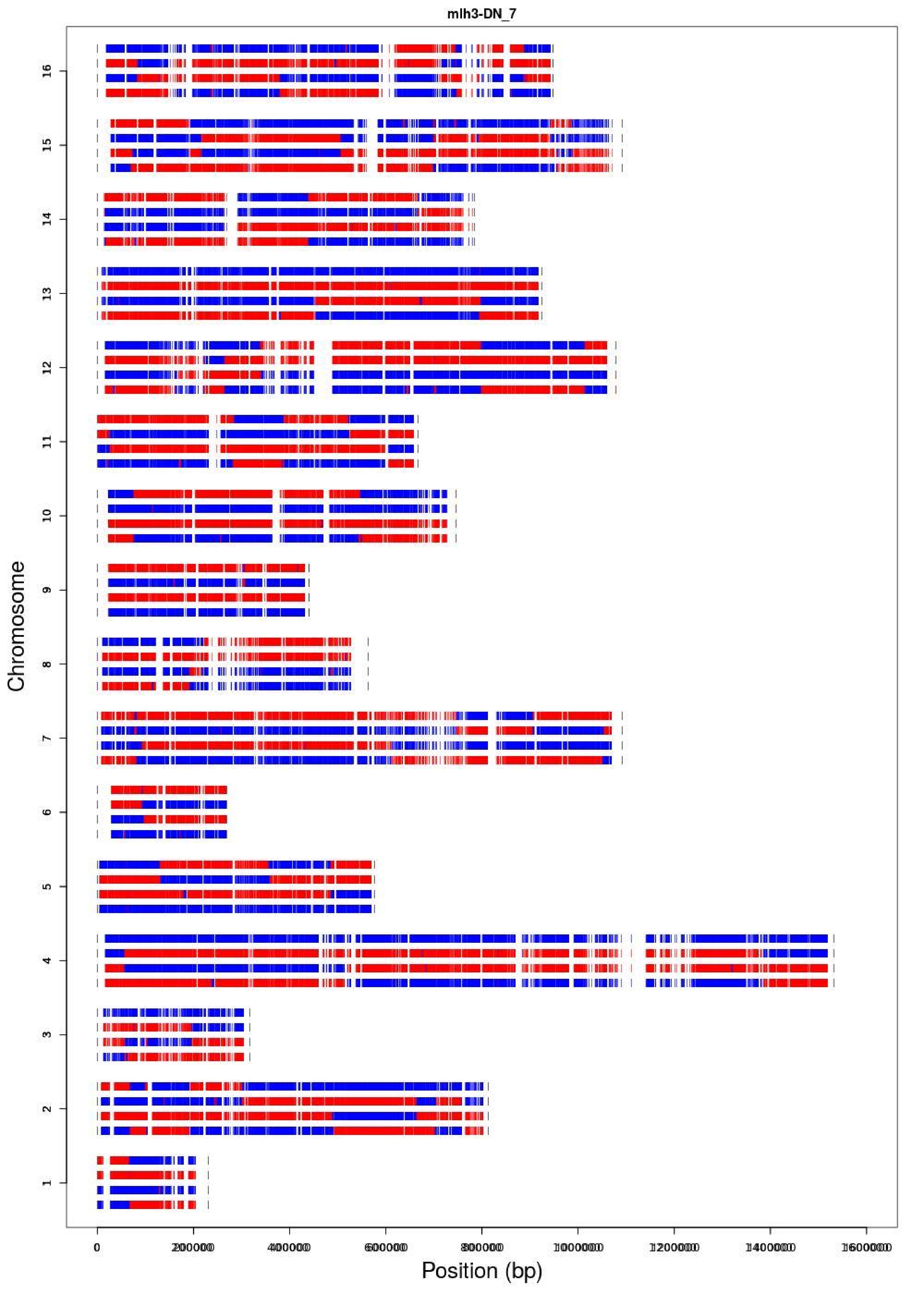

mlh3-DN_7
Chromosome
16
15
14
13
12
11
10
9
8
7
6
5
4
3
2
1
Position (bp)
0
200000
400000
600000
800000
1000000
1200000
1400000
1600000

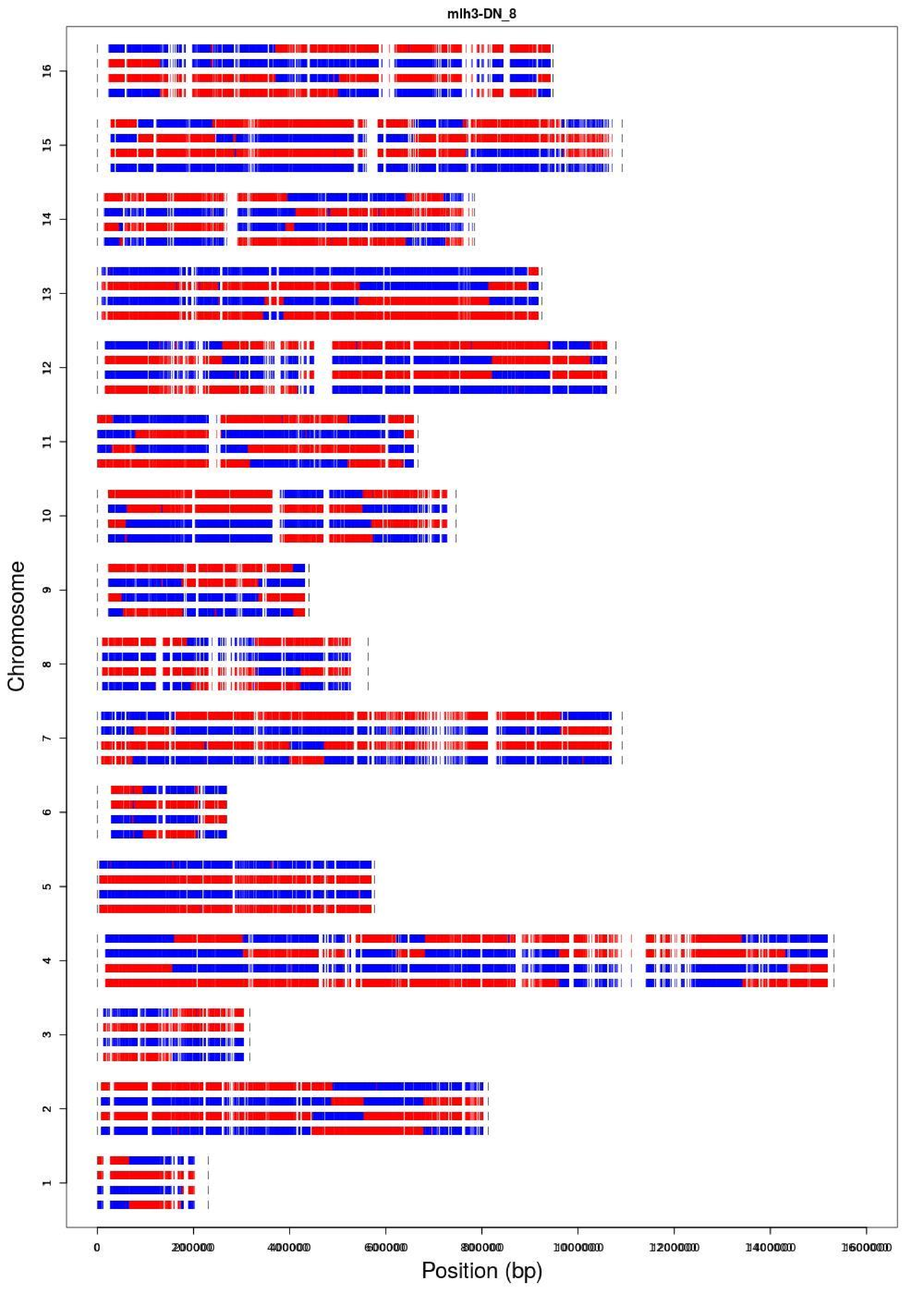
mlh3-DN_8
Chromosome
16
15
14
13
12
11
10
9
8
7
6
5
4
3
2
1
0
200000
400000
600000
800000
1000000
1200000
1400000
1600000
Position (bp)

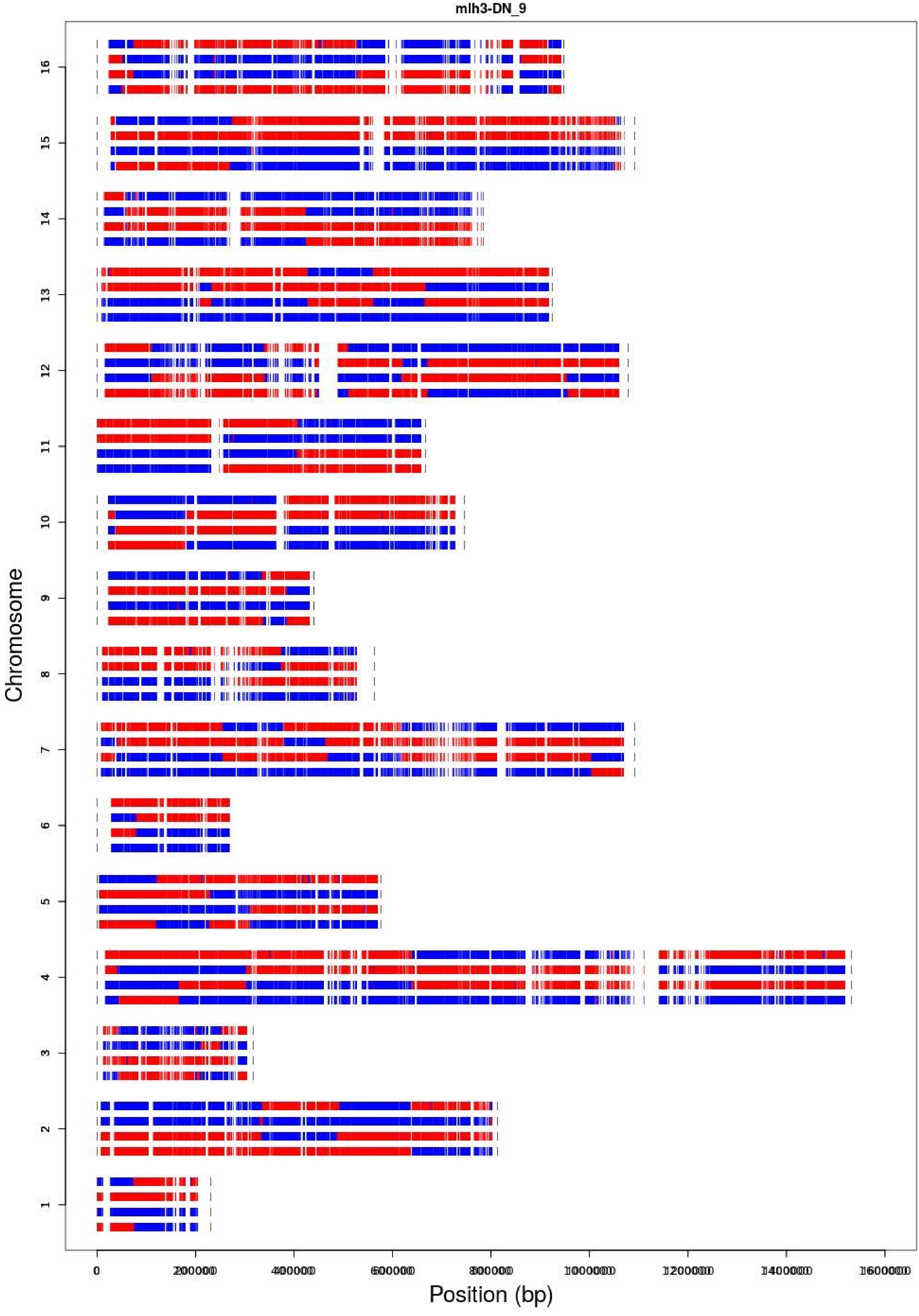
mlh3-DN_9
Chromosome
Position (bp)
16
15
14
13
12
11
10
9
8
7
6
5
4
3
2
1
0
200000
400000
600000
800000
1000000
1200000
1400000
1600000

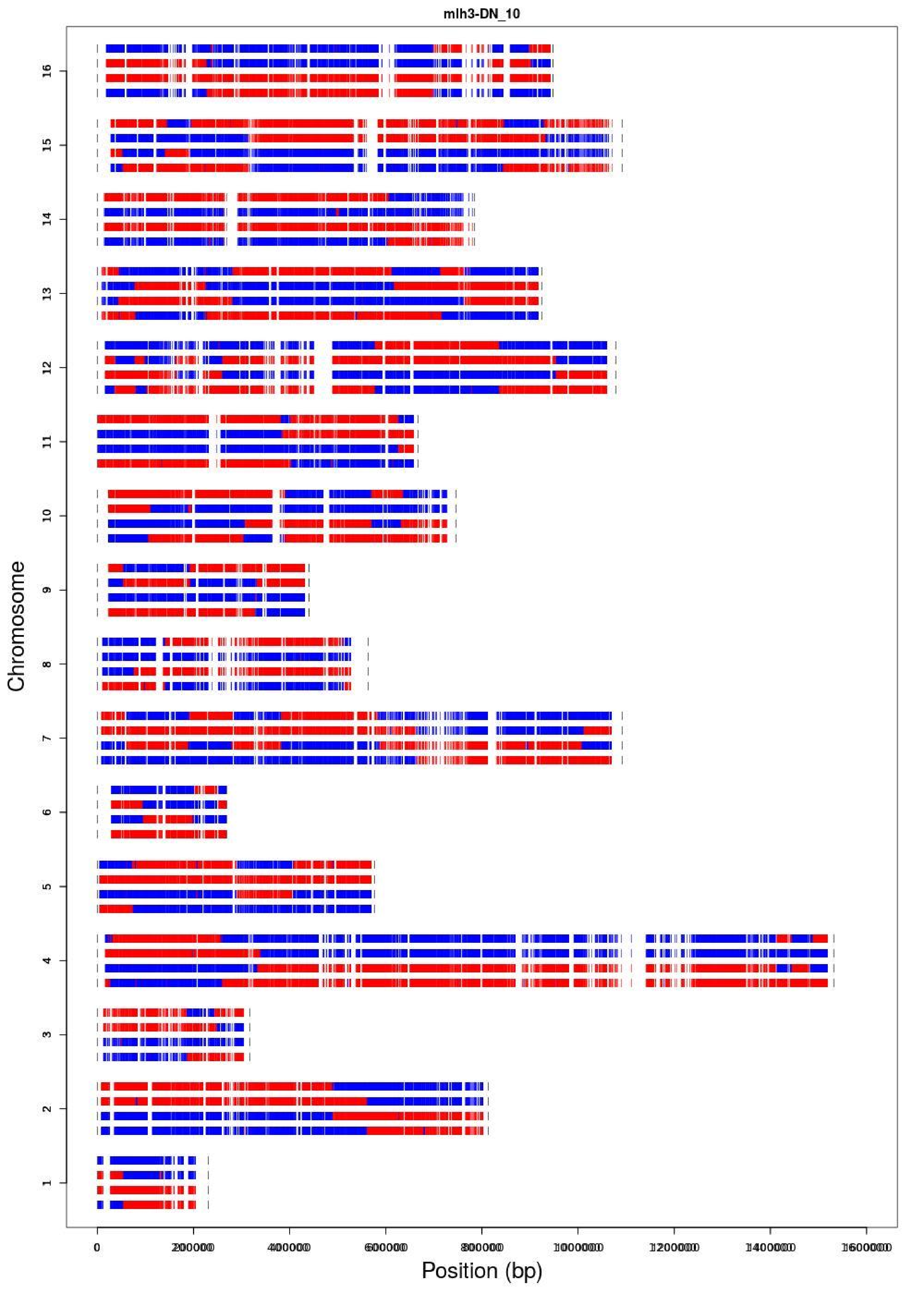

mlh3-DN_10
Chromosome
16
15
14
13
12
11
10
9
8
7
6
5
4
3
2
1
0
200000
400000
600000
800000
1000000
1200000
1400000
1600000
Position (bp)

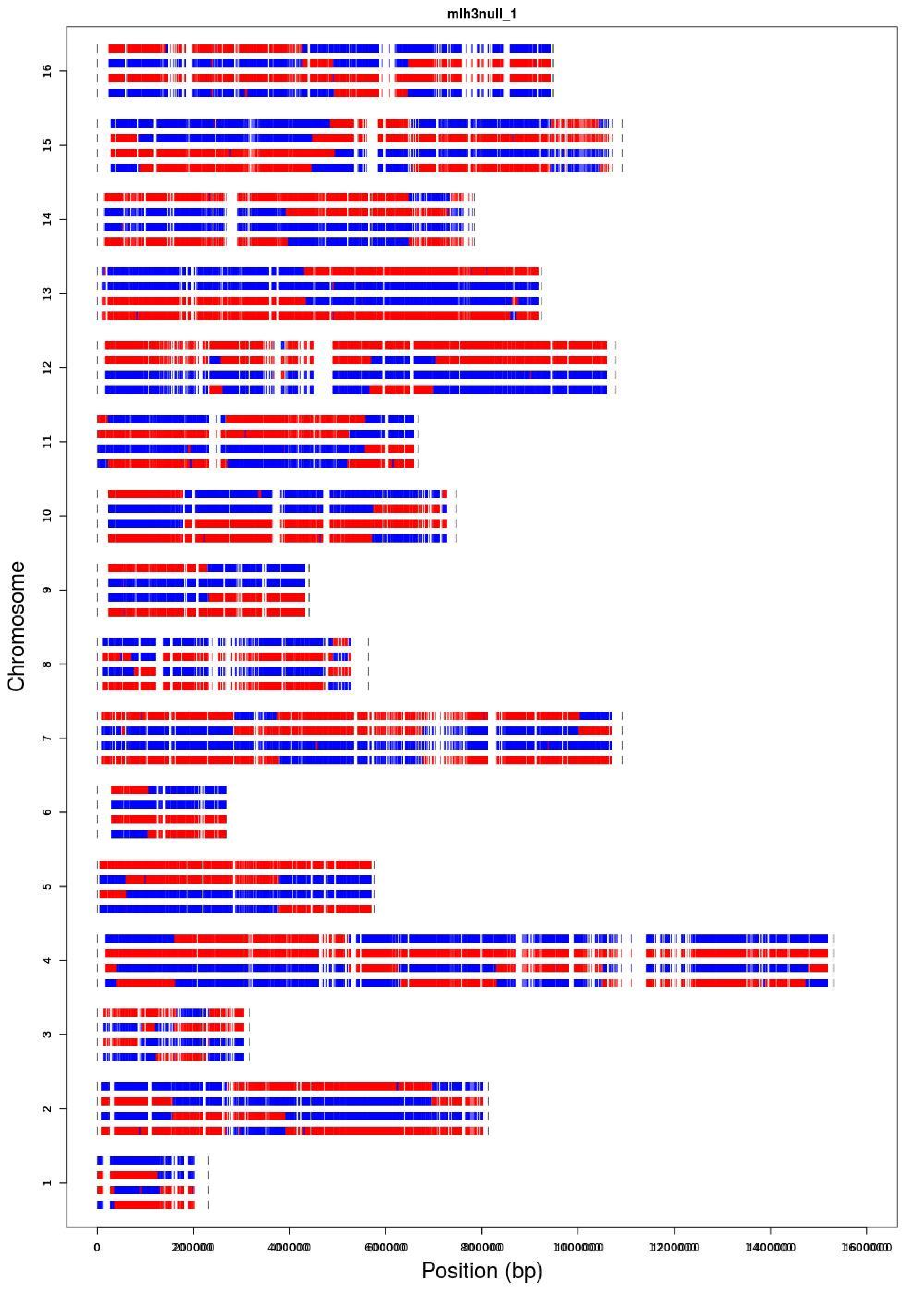
mlh3null_1
Chromosome
16
15
14
13
12
11
10
9
8
7
6
5
4
3
2
1
0
200000
400000
600000
800000
1000000
1200000
1400000
1600000
Position (bp)

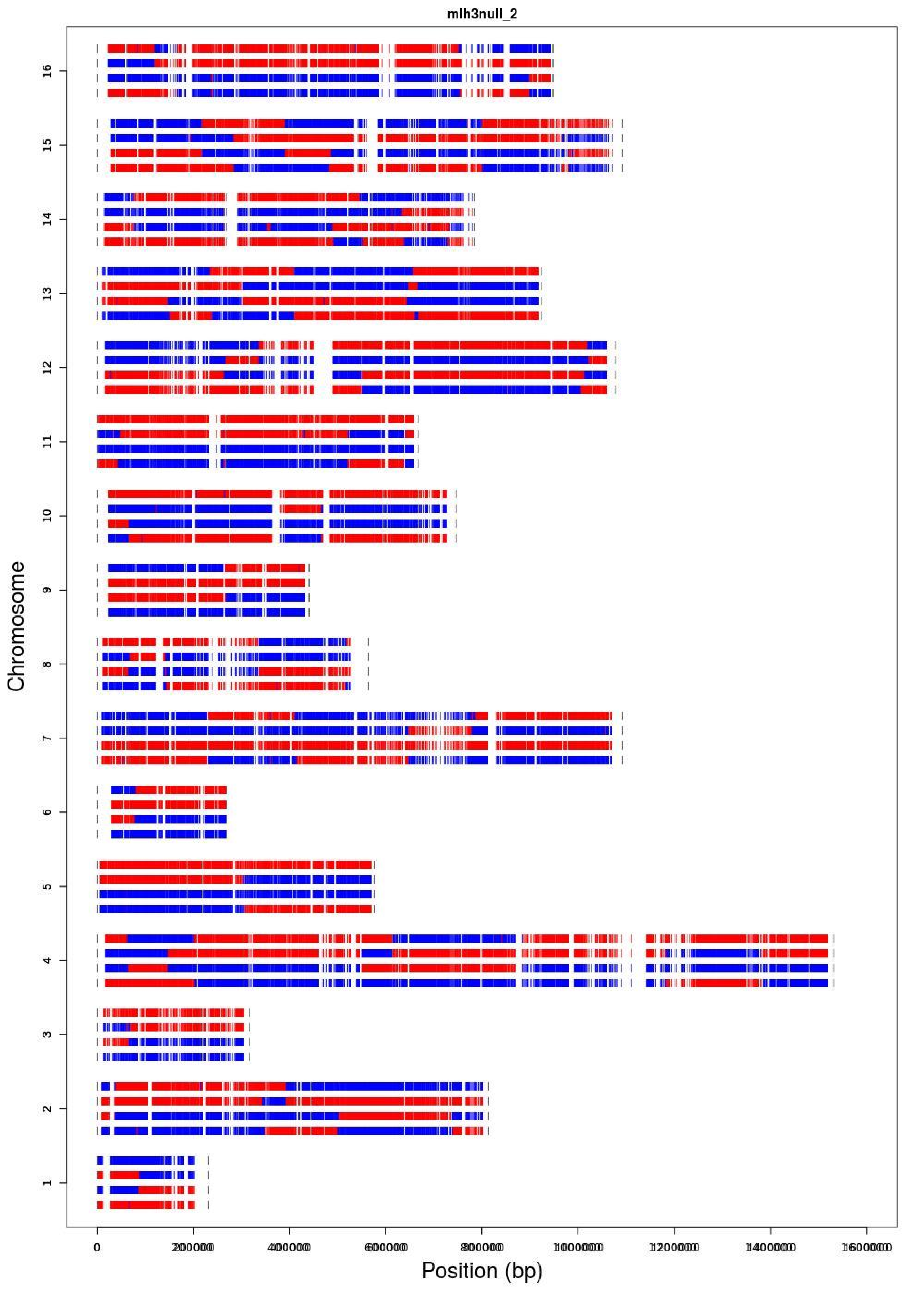
mlh3null_2
Chromosome
16
15
14
13
12
11
10
9
8
7
6
5
4
3
2
1
Position (bp)
0
200000
400000
600000
800000
1000000
1200000
1400000
1600000

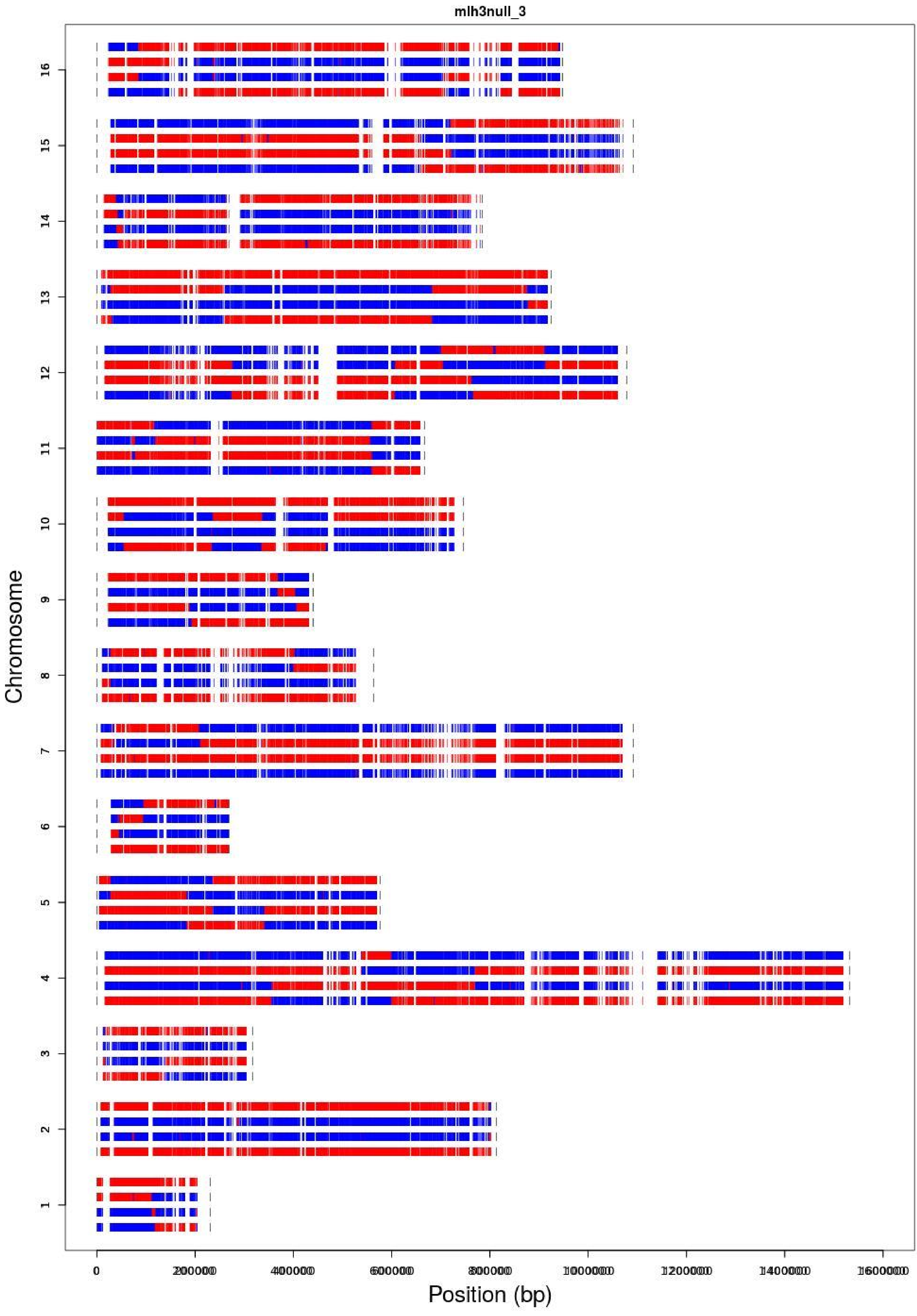

mlh3null_3
Chromosome
16
15
14
13
12
11
10
9
8
7
6
5
4
3
2
1
Position (bp)
0
200000
400000
600000
800000
1000000
1200000
1400000
1600000

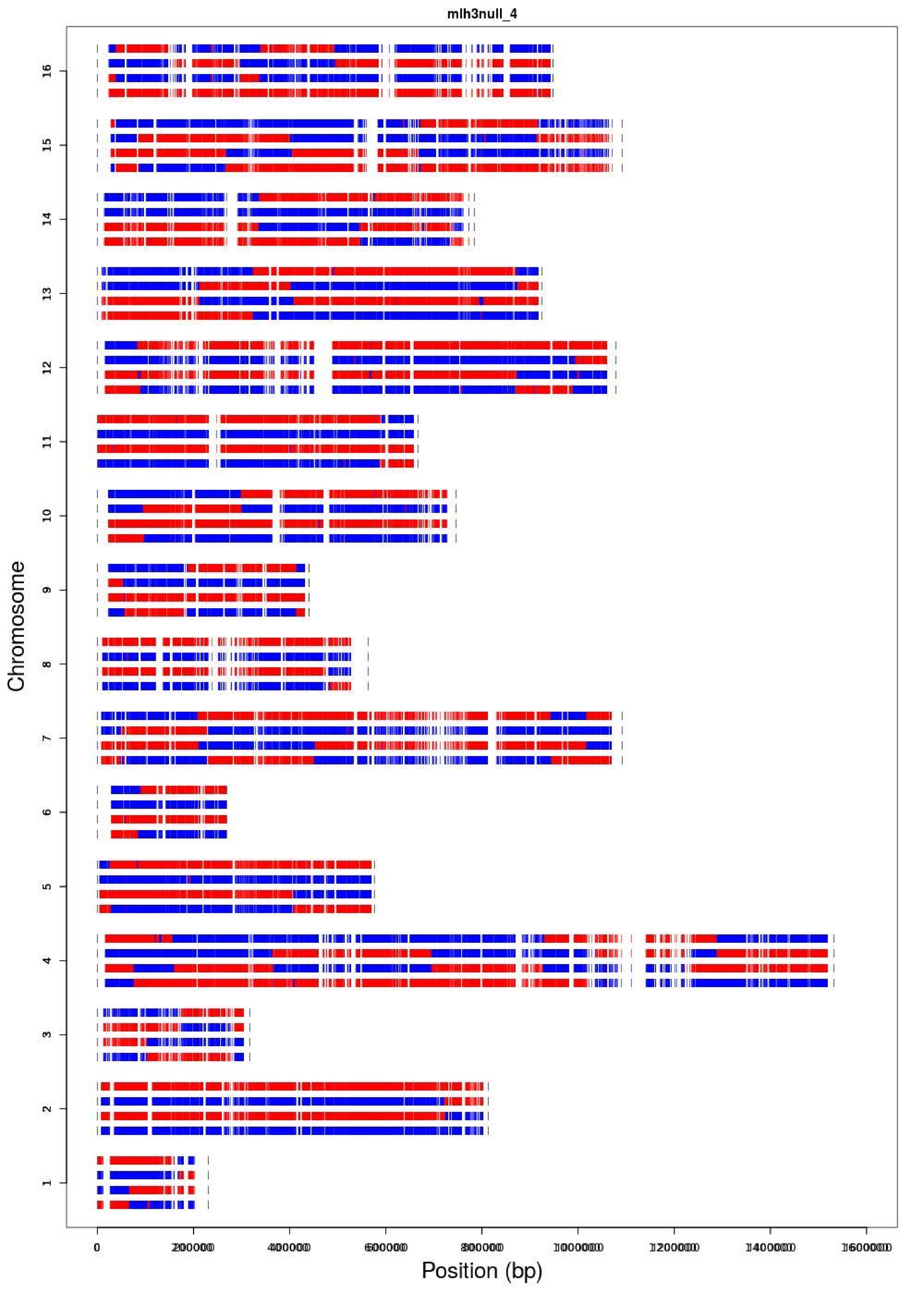

mlh3null_4
Chromosome
16
15
14
13
12
11
10
9
8
7
6
5
4
3
2
1
0
200000
400000
600000
800000
1000000
1200000
1400000
1600000
Position (bp)

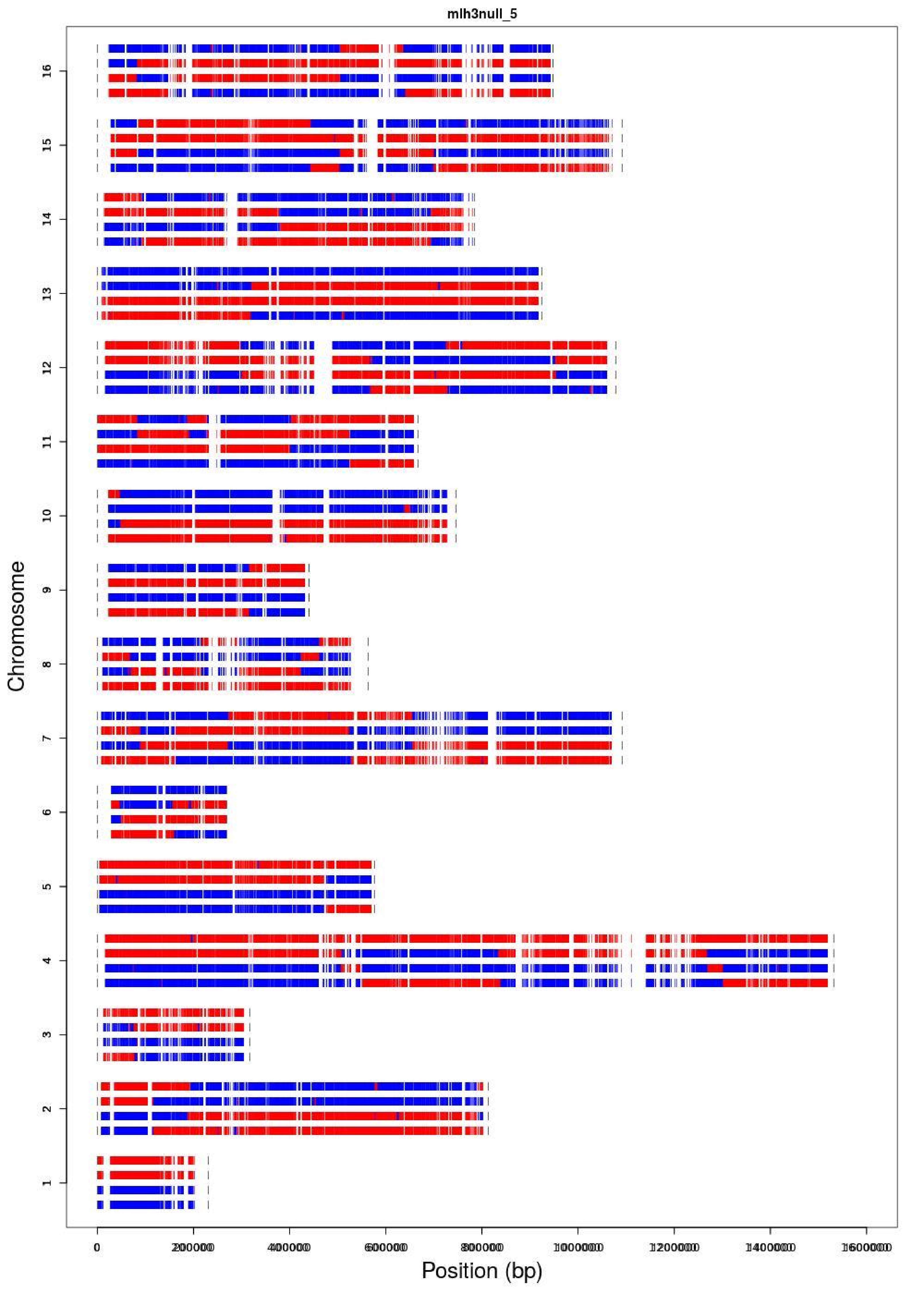
mlh3null_5
Chromosome
16
15
14
13
12
11
10
9
8
7
6
5
4
3
2
1
0
200000
400000
600000
800000
1000000
1200000
1400000
1600000
Position (bp)

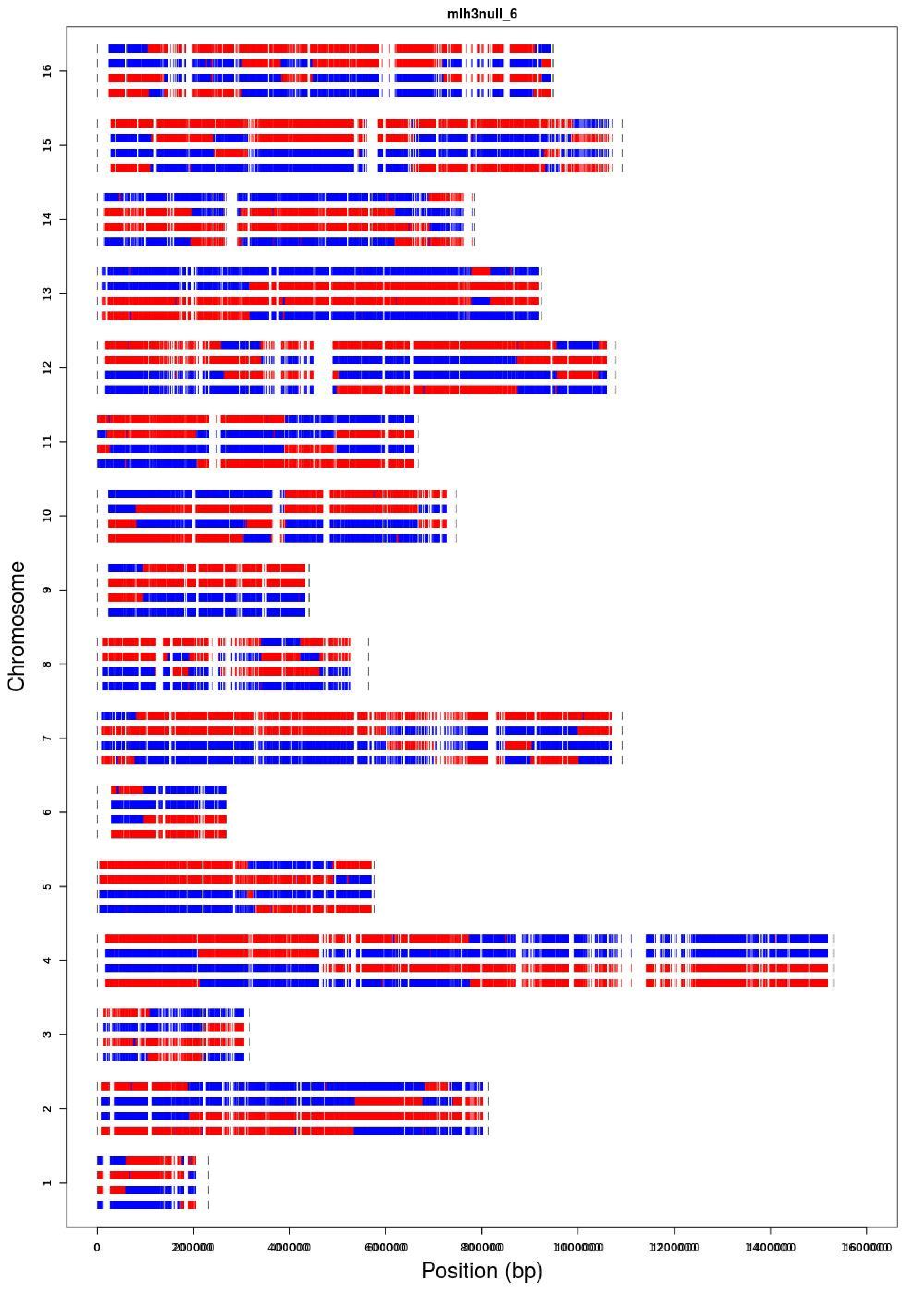

mlh3null_6
Chromosome
16
15
14
13
12
11
10
9
8
7
6
5
4
3
2
1
Position (bp)
0
200000
400000
600000
800000
1000000
1200000
1400000
1600000

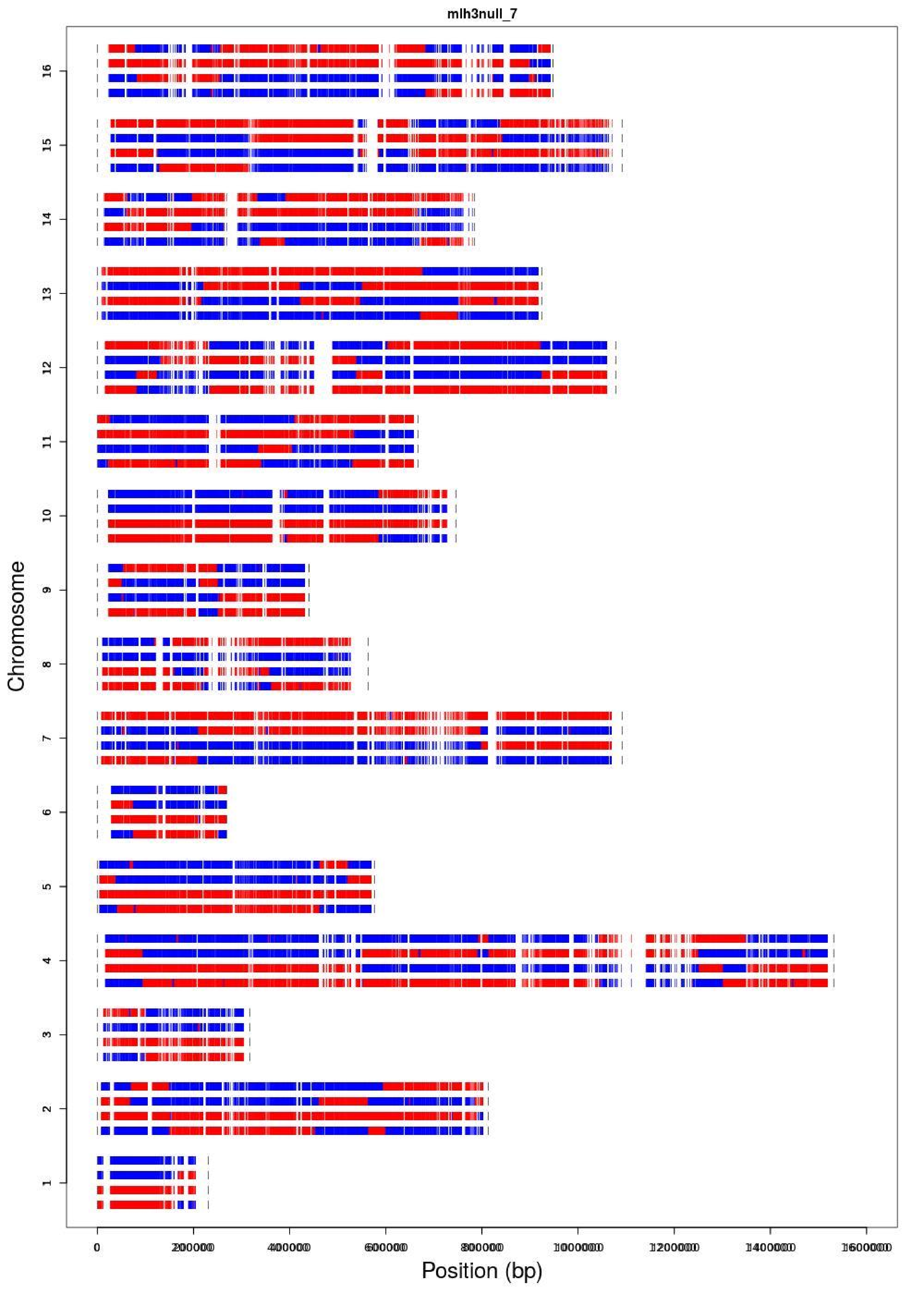
mlh3null_7
Chromosome
16
15
14
13
12
11
10
9
8
7
6
5
4
3
2
1
0
200000
400000
600000
800000
1000000
1200000
1400000
1600000
Position (bp)

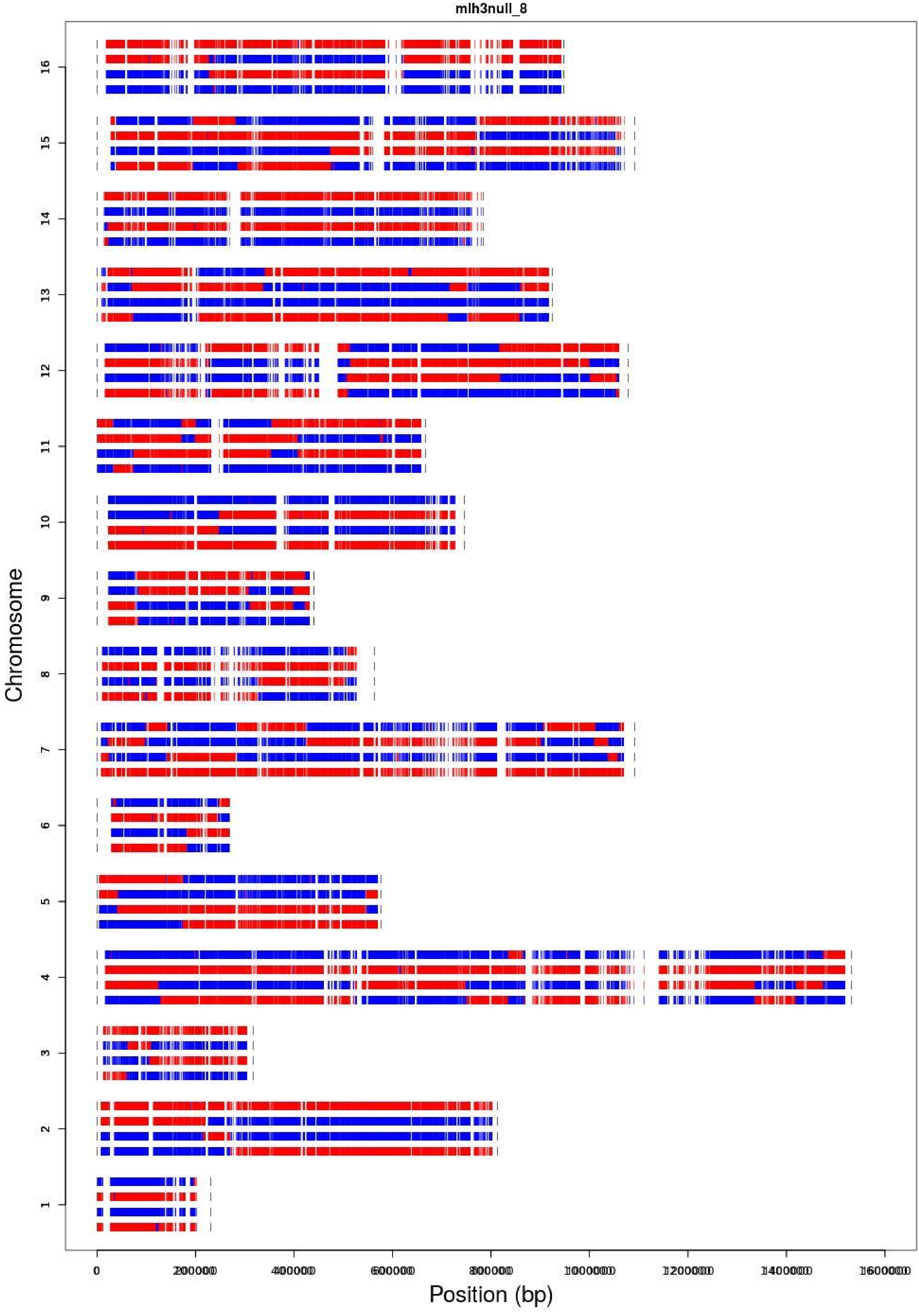

mlh3null_8
Chromosome
16
15
14
13
12
11
10
9
8
7
6
5
4
3
2
1
0
200000
400000
600000
800000
1000000
1200000
1400000
1600000
Position (bp)

mlh3null_9

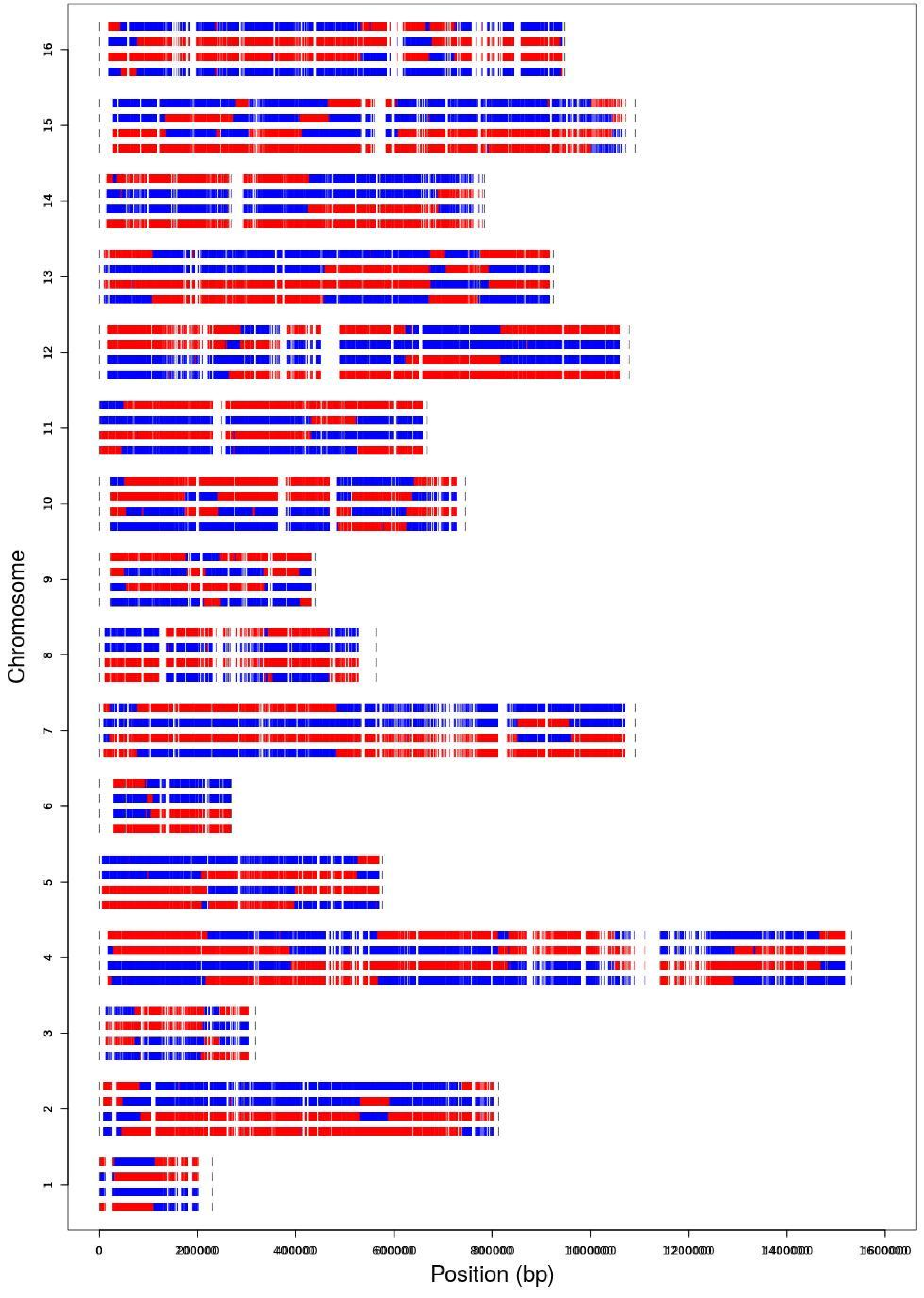

Chromosome
16
15
14
13
12
11
10
9
8
7
6
5
4
3
2
1
0
200000
400000
600000
800000
1000000
1200000
1400000
1600000
Position (bp)

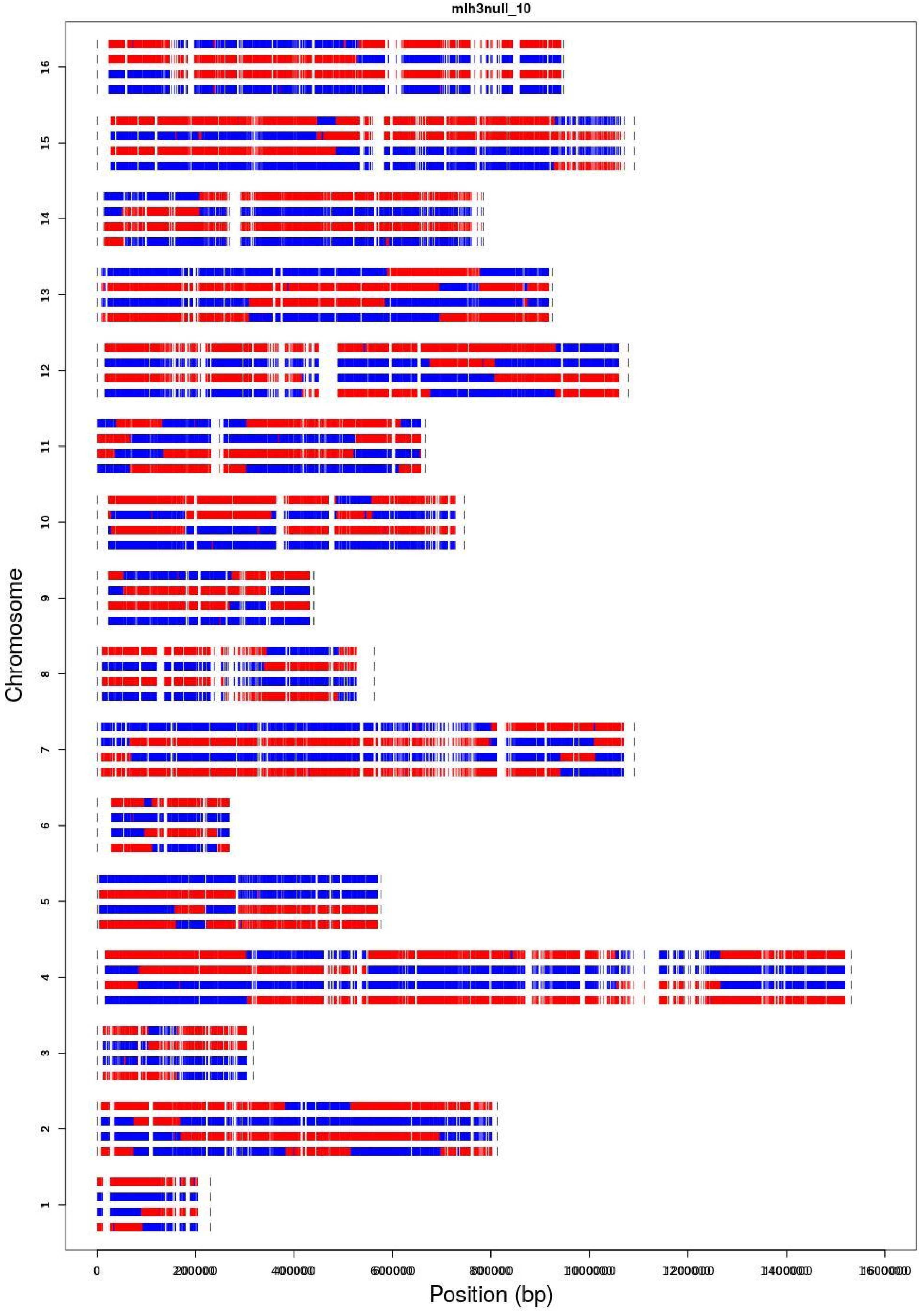

mlh3null_10
Chromosome
16
15
14
13
12
11
10
9
8
7
6
5
4
3
2
1
0
200000
400000
600000
800000
1000000
1200000
1400000
1600000
Position (bp)
