# Supplementary figures and images for: mlh3 mutations in baker’s yeast alter meiotic recombination outcomes by increasing noncrossover events genome-wide
Source: PLoS Genet. 2017 Aug 21;13(8):e1006974. doi: 10.1371/journal.pgen.1006974 (PMC5578695; doi:10.1371/journal.pgen.1006974)

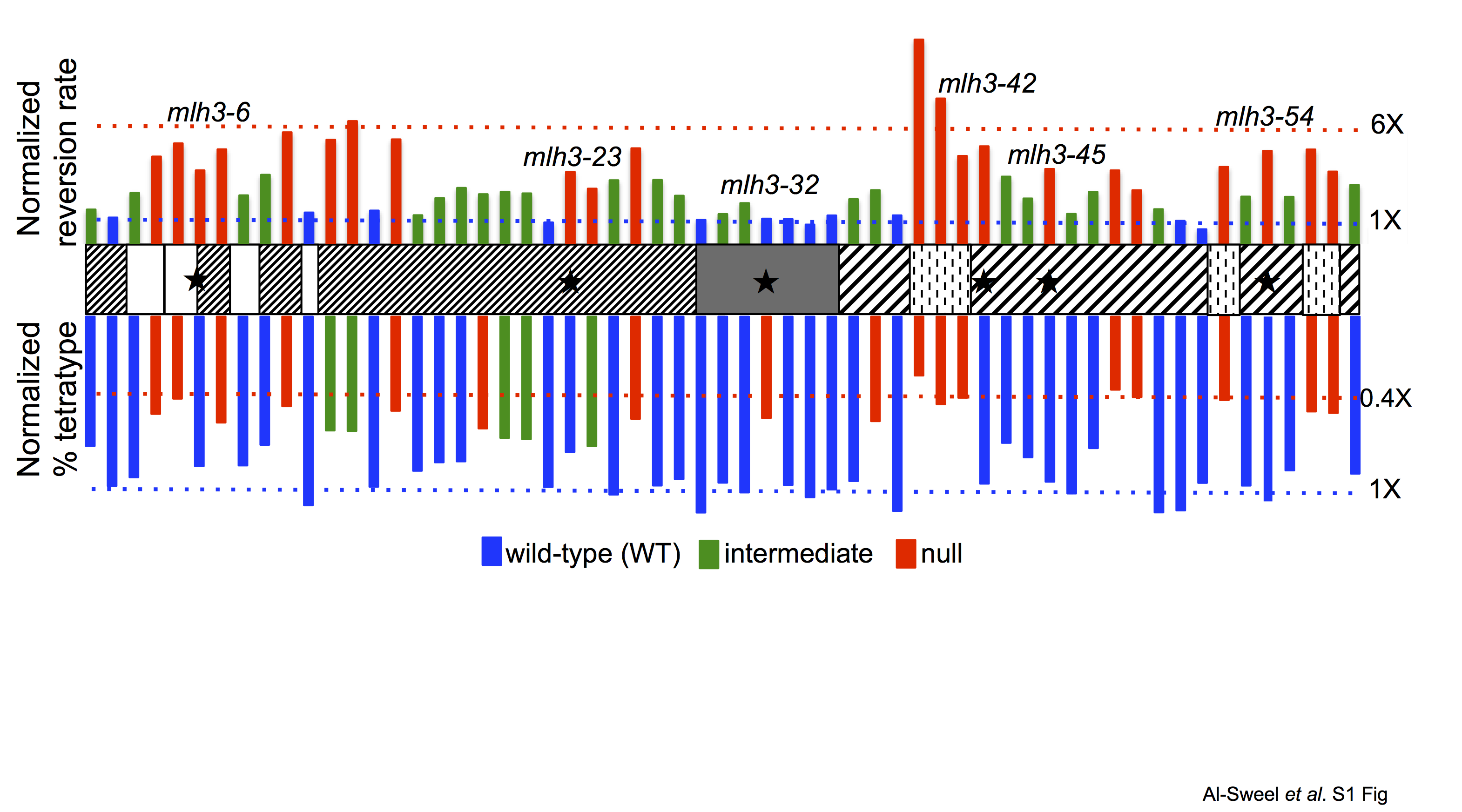

Supplement: S1 Fig — Mismatch repair (top) and crossing over (bottom) phenotype of MLH3 (blue) vs mlh3Δ (red) (Fig 3B). Mismatch repair was measured using the lys2-A14 reversion assay [55] and crossing over was measured using the assay [56] depicted in Fig 3A. Bars represent the median reversion rates and percent tetratype normalized to MLH3 (1X). The vertical bars indicate the approximate position of the mlh3 mutations analyzed in this study with the height of each bar corresponding to the phenotype relative to MLH3 (1X). Red indicates a null phenotype, blue indicates wild-type (WT), and green indicates intermediate. For mismatch repair (top), bars represent reversion rates of at least 10 independently tested cultures from two independently constructed strains presented here normalized to MLH3 median rate 1X = 1.43x10^-6^ (n = 140). For crossing over (bottom), bars represent percent tetratype of at least 250 tetrads from two independently constructed strains presented here normalized to MLH3 percent tetratype 1X = 36.7% (n = 226). Blue and red dotted lines represent MLH3 and mlh3Δ respectively. Black star indicates separation of function mutants (Table 1 and S2 File). (TIFF) [file pgen.1006974.s003.tiff]

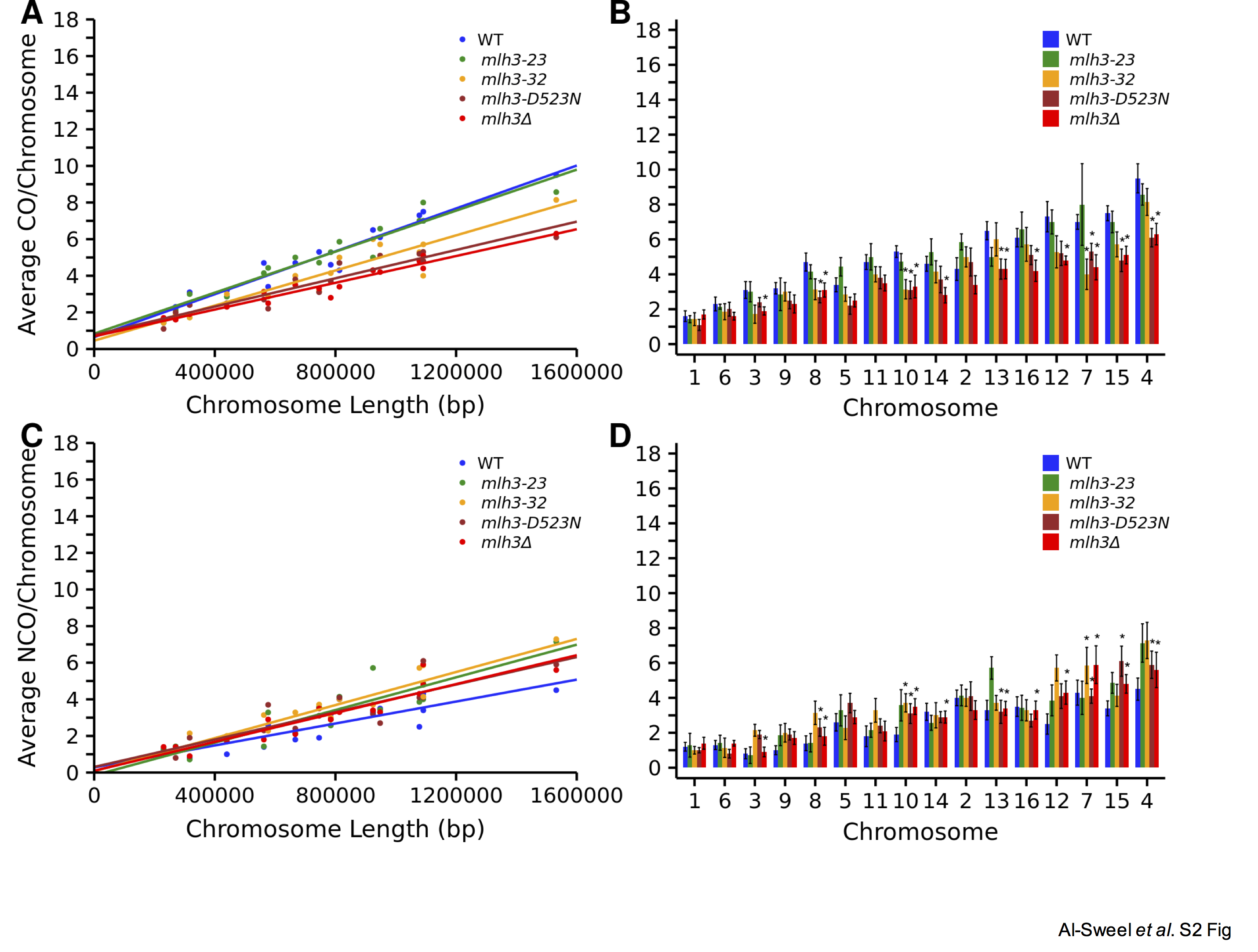

Supplement: S2 Fig — A. and C. Scatter plot of average crossover (CO, E2+E3) and noncrossover (NCO, E1) counts per chromosome against chromosome size [34]. The equations for the regression lines are: wild-type (CO = 5.85 x 10−6 x chr. size + 0.65; NCO = 2.99 x 10−6 x chr. size + 0.28);mlh3-23 (CO = 5.6 x 10−6 x chr. size + 0.84; NCO = 4.46 x 10−6 x chr. size − 0.15);mlh3-32 (CO = 4.79 x 10−6 x chr. size + 0.45; NCO = 4.51 x 10−6 x chr. size + 0.08);mlh3-D523N (CO = 3.8 x 10−6 x chr. size + 0.77; NCO = 3.75 x 10−6 x chr. size + 0.31);mlh3Δ (CO = 3.65 x 10−6 x chr. size + 0.69; NCO = 3.94 x 10−6 x chr. size + 0.1). B. and D. Bar plot of average crossover and noncrossover counts per chromosome. The asterisk symbol (*) marks chromosomes that have significant difference (two tailed t-test for difference in mean; P<0.05) in crossover / noncrossover counts compared to wild-type. Chromosomes are arranged by size from left to right. Error bars are mean ± standard error (S2 File). (TIFF) [file pgen.1006974.s004.tiff]

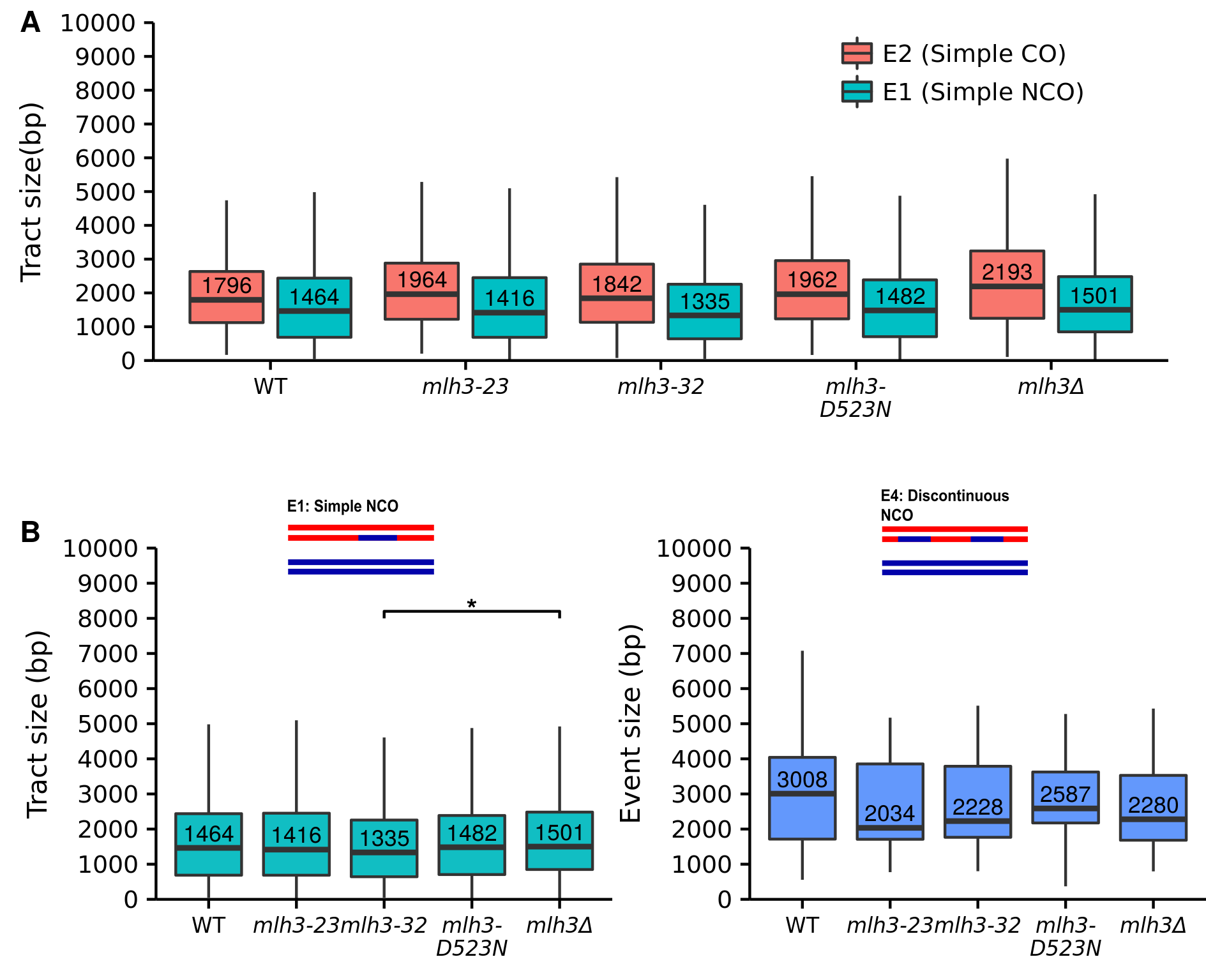

Supplement: S3 Fig — A. Distribution of gene conversion tract lengths associated with simple NCO (E1) and CO (E2) events (S2 File). B. Distribution of gene conversion tract lengths associated with simple NCO (E1) and discontinuous NCO (E4) events. * p ≤ 0.05 comparing mlh3Δ to mlh3-32. In A and B the minimum, first quantile, median, third quantile, and maximum count are indicated in the box plot. Outlier points are not shown (S2 File). (TIFF) [file pgen.1006974.s005.tiff]

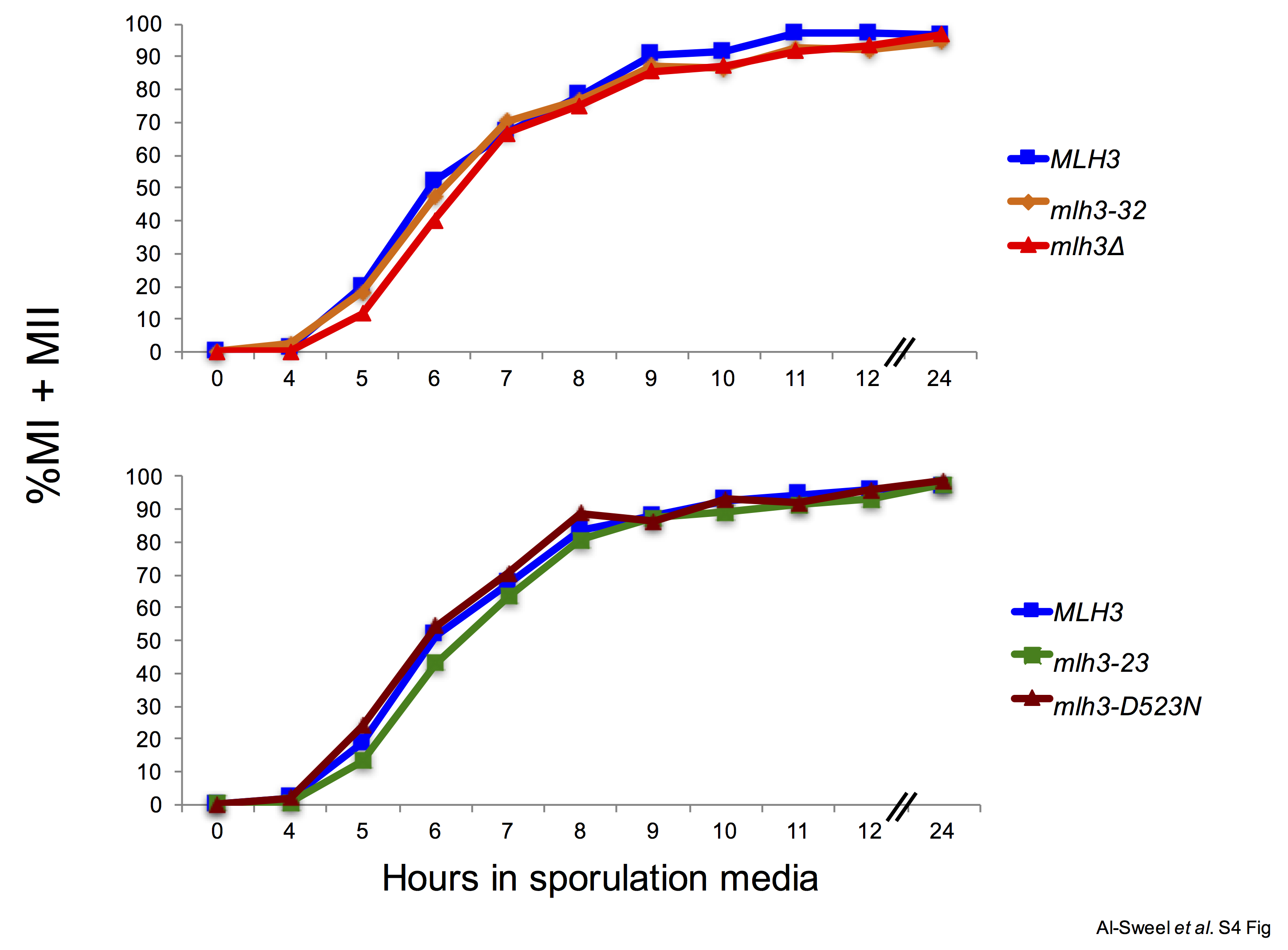

Supplement: S4 Fig — Representative time courses showing the completion of the MI division (MI+MII) in wild-type, mlh3-23, mlh3-32, mlh3-D523N and mlh3Δ strains. Cells with two, three, or four nuclei were counted as having completed MI (MI+MII). All strains for a single time course were grown in the same batch of media under identical conditions. Two independent transformants were measured per allele (S2 File). (TIFF) [file pgen.1006974.s006.tiff]

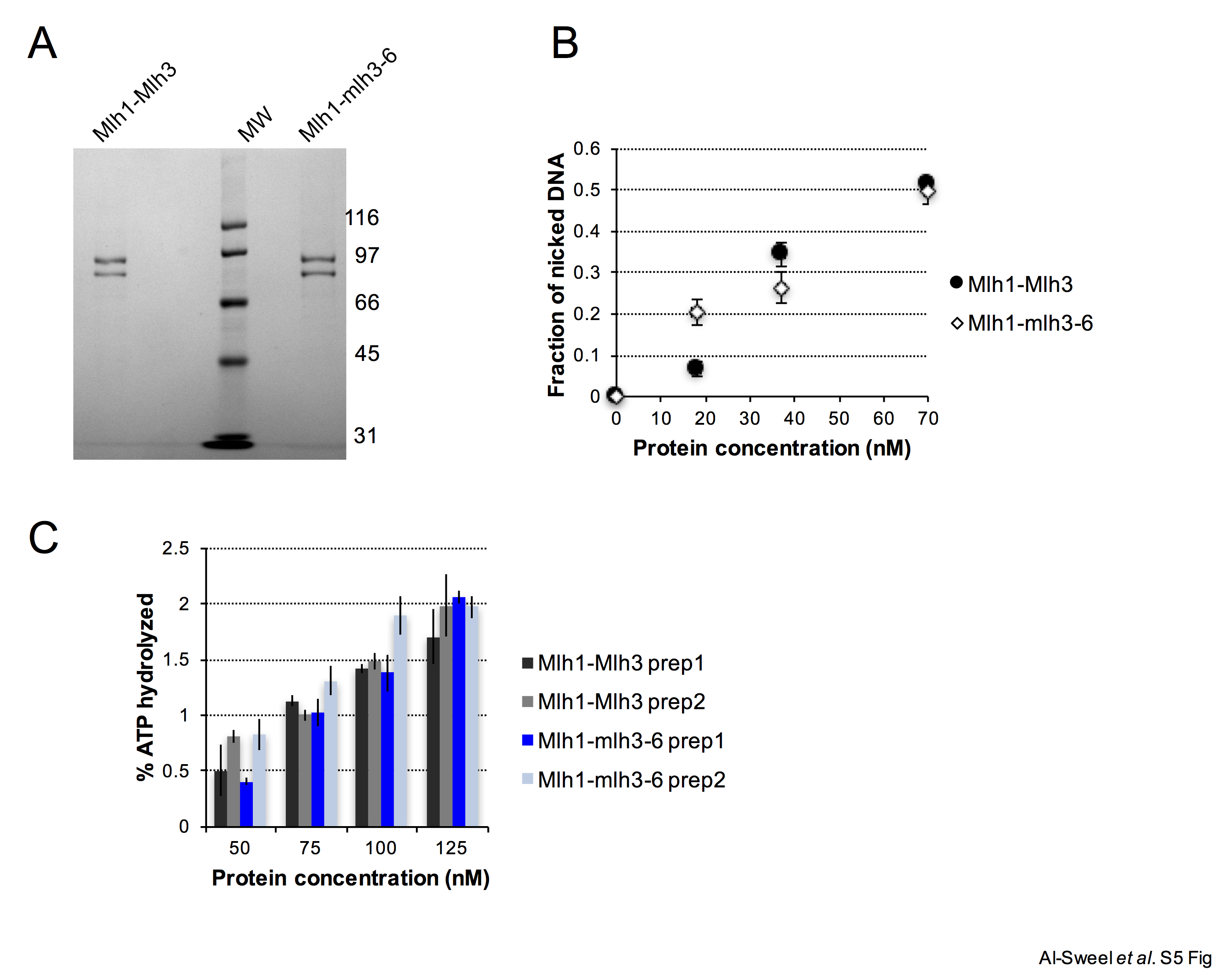

Supplement: S5 Fig — A. SDS-PAGE analysis of purified Mlh1-Mlh3 and Mlh1-mlh3-6. Coomassie Blue R250-stained 8% Tris-glycine gel. 0.5 μg of each protein is shown. MW = Molecular Weight Standards from top to bottom-116, 97, 66, 45, 31 kD). B, C. Mlh1-Mlh3 and Mlh1-mlh3-6 (18, 37, 70 nM) were incubated with 2.2 nM supercoiled pBR322 DNA, analyzed in agarose gel electrophoresis, and endonuclease activity was quantified (average of 6 independent experiments presented +/-SD) as described in the Methods (S2 File). C. ATPase assays were performed as described in Rogacheva et al. [16], but contained the indicated amounts of Mlh1-Mlh3 and Mlh1-mlh3-6 incubated with 100 μM 32P-γ-ATP. Reactions were performed in duplicate for two separate purifications of each, and the average values, +/-SD, are presented (S2 File). (TIFF) [file pgen.1006974.s007.tiff]
