# Supplementary material for: mlh3 mutations in baker’s yeast alter meiotic recombination outcomes by increasing noncrossover events genome-wide
Source: PLoS Genet. 2017 Aug 21;13(8):e1006974. doi: 10.1371/journal.pgen.1006974 (PMC5578695; doi:10.1371/journal.pgen.1006974)
Supplement: S1 Table — EAY3252, EAY3255 and derivatives, and EAY3486 are SK1 strains that contain spore autonomous fluorescent markers described in Thacker et al. [56]. EAY1112 and EAY2413 contain chromosome XV markers described in Argueso et al. [7]. KTY strains were used for whole genome recombination mapping as described in Krishnaprasad et al. [64] and Oke et al. [34]. (PDF) [file pgen.1006974.s008.pdf]

**S1 Table. Yeast strains used in this study.**

| Strain | Genotype | |
|---|---|---|
| EAY3252 | *MATα ho::hisG, ura3, leu2::hisG, trp1::hisG, ADE2, HIS4, CEN8Tomato::LEU2, MLH3, lys2::insE-A14* | |
| EAY3255 | *MATα ho::hisG, ura3, leu2::hisG, trp1::hisG, ADE2, his4xB, CEN8Tomato::LEU2, mlh3Δ::NATMX, lys2::insE-A14* | |
| | | amino acid substitution |
| EAY3490-91 | Same as EAY3255, but *mlh3-1::KANMX* | H4A,R6A,K7A |
| EAY3492-93 | Same as EAY3255, but *mlh3-2::KANMX* | E14A,R15A,K17A |
| EAY3494-95 | Same as EAY3255, but *mlh3-3::KANMX* | R30A,E31A |
| EAY3496-97 | Same as EAY3255, but *mlh3-4::KANMX* | D38A,H40A |
| EAY3498-99 | Same as EAY3255, but *mlh3-5::KANMX* | D59A,D60A |
| EAY3500-01 | Same as EAY3255, but *mlh3-6::KANMX* | R66A,D68A |
| EAY3502-03 | Same as EAY3255, but *mlh3-7::KANMX* | K80A,R82A,K83A |
| EAY3504-05 | Same as EAY3255, but *mlh3-8::KANMX* | R96A,D98A |
| EAY3506-07 | Same as EAY3255, but *mlh3-9::KANMX* | K114A,K115A,K166A,D117A |
| EAY3508-09 | Same as EAY3255, but *mlh3-10::KANMX* | R124A,K125A |
| EAY3510-11 | Same as EAY3255, but *mlh3-11::KANMX* | R151A,R152A,K154A |
| EAY3512-13 | Same as EAY3255, but *mlh3-12::KANMX* | E162A,D163A |
| EAY3514-15 | Same as EAY3255, but *mlh3-13::KANMX* | R171A,R172A,R173A |
| EAY3516-17 | Same as EAY3255, but *mlh3-14::KANMX* | K176A,E177A,E178A |
| EAY3518-19 | Same as EAY3255, but *mlh3-15::KANMX* | K188A,D190A |
| EAY3520-21 | Same as EAY3255, but *mlh3-16::KANMX* | D209A,K210A,R212A |
| EAY3522-23 | Same as EAY3255, but *mlh3-17::KANMX* | R220A,K222A |
| EAY3524-25 | Same as EAY3255, but *mlh3-18::KANMX* | K230A, H231A |
| EAY3526-27 | Same as EAY3255, but *mlh3-19::KANMX* | K252A, K253A |
| EAY3528-29 | Same as EAY3255, but *mlh3-20::KANMX* | R273A, K275A, D276A |
| EAY3530-31 | Same as EAY3255, but *mlh3-21::KANMX* | R285A, R286A |
| EAY3532-33 | Same as EAY3255, but *mlh3-22::KANMX* | E307A, K308A |
| EAY3534-35 | Same as EAY3255, but *mlh3-23::KANMX* | K314A, K316A |
| EAY3536-37 | Same as EAY3255, but *mlh3-24::KANMX* | R323A, H325A |
| EAY3538-39 | Same as EAY3255, but *mlh3-25::KANMX* | D331A, R333A |
| EAY3540-41 | Same as EAY3255, but *mlh3-26::KANMX* | D339A, D340A |
| EAY3542-43 | Same as EAY3255, but *mlh3-27::KANMX* | K347A, K348A |
| EAY3544-45 | Same as EAY3255, but *mlh3-28::KANMX* | H354A, R356A |
| EAY3546-47 | Same as EAY3255, but *mlh3-29::KANMX* | D379A, K380A, D382A |
| EAY3548-49 | Same as EAY3255, but *mlh3-30::KANMX* | D399A, R401A |
| EAY3550-51 | Same as EAY3255, but *mlh3-31::KANMX* | K406A, R407A |
| EAY3552-53 | Same as EAY3255, but *mlh3-32::KANMX* | K414A, K416A |
| EAY3554-55 | Same as EAY3255, but *mlh3-33::KANMX* | E442A, K443A, K445A |
| EAY3556-57 | Same as EAY3255, but *mlh3-34::KANMX* | R448A, D450A |
| EAY3558-59 | Same as EAY3255, but *mlh3-35::KANMX* | E471D, D472A, D474A |
| EAY3560-61 | Same as EAY3255, but *mlh3-36::KANMX* | K492A, E494A |
| EAY3562-63 | Same as EAY3255, but *mlh3-37::KANMX* | D500A, K501A, K502A |
| EAY3564-65 | Same as EAY3255, but *mlh3-38::KANMX* | H514A, R516A |
| EAY3566-67 | Same as EAY3255, but *mlh3-39::KANMX* | D523A, H525A |
| EAY3568-69 | Same as EAY3255, but *mlh3-40::KANMX* | D528A, E529A, R530A |
| EAY3570-71 | Same as EAY3255, but *mlh3-41::KANMX* | R532A, E534A, E535A |
| EAY3572-73 | Same as EAY3255, but *mlh3-42::KANMX* | R552A, D553A, K555A, D556A |
| EAY3574-75 | Same as EAY3255, but *mlh3-43::KANMX* | D562A, R563A, E565A |
| EAY3576-77 | Same as EAY3255, but *mlh3-44::KANMX* | K570A, H571A |

| | | |
|---|---|---|
| EAY3578-79 | Same as EAY3255, but *mlh3-45::KANMX* | K577A, K578A |
| EAY3580-81 | Same as EAY3255, but *mlh3-46::KANMX* | E596A, K598A |
| EAY3582-83 | Same as EAY3255, but *mlh3-47::KANMX* | D611A, K612A, D613A |
| EAY3584-85 | Same as EAY3255, but *mlh3-48::KANMX* | H622A, H624A, D625A |
| EAY3586-87 | Same as EAY3255, but *mlh3-49::KANMX* | K627A, D628A, K630A, K631A |
| EAY3588-89 | Same as EAY3255, but *mlh3-50::KANMX* | H638A, E640A, H642A |
| EAY3590-91 | Same as EAY3255, but *mlh3-51::KANMX* | D646A, K647A |
| EAY3592-93 | Same as EAY3255, but *mlh3-52::KANMX* | H662A, E663A |
| EAY3594-95 | Same as EAY3255, but *mlh3-53::KANMX* | D678A, E679A |
| EAY3596-97 | Same as EAY3255, but *mlh3-54::KANMX* | R682A, E684A |
| EAY3598-99 | Same as EAY3255, but *mlh3-55::KANMX* | R694A, H696A |
| EAY3600-01 | Same as EAY3255, but *mlh3-56::KANMX* | E713A, K715A |
| EAY3701-02 | Same as EAY3255, but *mlh3-57::KANMX* | C670A |
| EAY3703-04 | Same as EAY3255, but *mlh3-58::KANMX* | C701A |
| EAY3705-06 | Same as EAY3255, but *mlh3-59::KANMX* | H703A |
| EAY3707-08 | Same as EAY3255, but *mlh3-60::KANMX* | *716-DWSSFSKDYEI* |
| EAY3819-20 | Same as EAY3255, but *mlh3-D523N::KANMX* | |
| EAY3339 | *MATa ho::LYS2, lys2, ura3, leu2::hisG, trp1::hisG, THR1::m-Cerulean-TRP1* | |
| EAY3486 | Same as EAY3339, but *mlh3Δ::NATMX* | |
| EAY1112 | *MATα ho::hisG, trp1::hisG, leu2::hisG, lys2, ura3, ade2::hisG, his3Δ::hisG, TRP1insertion@CENXV* | |
| EAY1848 | Same as EAY1112, but *mlh3Δ::KANMX* | |
| EAY3712 | Same as EAY1112, but *mlh3Δ::URA3* | |
| EAY3713-14 | Same as EAY1112, but *mlh3-6::KANMX* | |
| EAY3715-16 | Same as EAY1112, but *mlh3-23::KANMX* | |
| EAY3717-18 | Same as EAY1112, but *mlh3-32::KANMX* | |
| EAY3719-20 | Same as EAY1112, but *mlh3-42::KANMX* | |
| EAY3721-22 | Same as EAY1112, but *mlh3-45::KANMX* | |
| EAY3723-24 | Same as EAY1112, but *mlh3-54::KANMX* | |
| EAY2413 | *MATa trp1:hisG, leu2::hisG, ho::hisG, ura3, lys2, URA3insertion@CENXV, LEU2insertion@chromXV, LYS2 insertion at position 505193 on chromosome XV, mlh3Δ::NATMX* | |
| S288c | *MATa ho lys5* | |
| YJM789 | *MATα ho::hisG lys2 cyh* | |
| KTY618 | Same as YJM789, but *SK1-MLH1::natMX4, mlh3Δ::kanMX4* | |
| KTY610 | Same as S288c, but *SK1-MLH3::kanMX4, mlh1Δ::hphMX4* | |
| KTY616 | Same as S288c, but *SK1-mlh3-23::kanMX4, mlh1Δ::hphMX4* | |
| KTY614 | Same as S288c, but *SK1-mlh3-32::kanMX4, mlh1Δ::hphMX4* | |
| KTY621 | Same as S288c, but *SK1-mlh3-D523N::kanMX4, mlh1Δ::hphMX4* | |
| KTY626 | Same as S288c, but *mlh3Δ::kanMX4, mlh1Δ::hphMX4* | |

EAY3252, EAY3255 and derivatives, and EAY3486 are SK1 strains that contain spore autonomous fluorescent markers described in Thacker et al. [56]. EAY1112 and EAY2413 contain chromosome XV markers described in Argueso et al. [7]. KTY strains were used for whole genome recombination mapping as described in Krishnaprasad et al. [64] and Oke et al. [34].
