# Supplementary material for: mlh3 mutations in baker’s yeast alter meiotic recombination outcomes by increasing noncrossover events genome-wide
Source: PLoS Genet. 2017 Aug 21;13(8):e1006974. doi: 10.1371/journal.pgen.1006974 (PMC5578695; doi:10.1371/journal.pgen.1006974)
Supplement: S2 Table — The indicated haploid strains were mated to form the diploids with the relevant genotype shown. (PDF) [file pgen.1006974.s009.pdf]

S2 Table. Diploid strains used to measure % tetratype, spore viability, meiotic progression, genetic map distances and for whole genome recombination mapping.

Diploid strain	Relevant genotype
Spore autonomous fluorescent protein assay [56], meiotic progression assay [93], and Sgs1 overexpression.	
EAY3252/EAY3486	*MLH3/mlh3Δ*
EAY3255/EAY3486	*mlh3Δ/mlh3Δ*
EAY3490 to EAY3708/EAY3486	*mlh3-1 to mlh3-60/mlh3Δ*
EAY3339/EAY3552-53	*MLH3/mlh3-32*
EAY3819-20/EAY3486	*mlh3-D523N/mlh3Δ*
Tetrad analysis [7]	
EAY1112/EAY2413	*MLH3/mlh3Δ*
EAY1848/EAY2413	*mlh3Δ/mlh3Δ*
EAY3713-14/EAY2413	*mlh3-6/mlh3Δ*
EAY3715-16/EAY2413	*mlh3-23/mlh3Δ*
EAY3717-18/EAY2413	*mlh3-32/mlh3Δ*
EAY3719-20/EAY2413	*mlh3-42/mlh3Δ*
EAY3721-22/EAY2413	*mlh3-45/mlh3Δ*
EAY3723-24/EAY2413	*mlh3-54/mlh3Δ*
Whole genome recombination mapping [34, 64]	
KTY618/KTY610	*SK1-MLH1/mlh1Δ, SK1-MLH3/mlh3Δ*
KTY618/KTY616	*SK1-MLH1/mlh1Δ, SK1-mlh3-23/mlh3Δ*
KTY618/KTY614	*SK1-MLH1/mlh1Δ, SK1-mlh3-32/mlh3Δ*
KTY618/KTY621	*SK1-MLH1/mlh1Δ, SK1-mlh3-D523N/mlh3Δ*
KTY618/KTY626	*SK1-MLH1/mlh1Δ, mlh3Δ/mlh3Δ*

The indicated haploid strains were mated to form the diploids with the relevant genotype shown.
