# Supplementary material for: mlh3 mutations in baker’s yeast alter meiotic recombination outcomes by increasing noncrossover events genome-wide
Source: PLoS Genet. 2017 Aug 21;13(8):e1006974. doi: 10.1371/journal.pgen.1006974 (PMC5578695; doi:10.1371/journal.pgen.1006974)
Supplement: S3 Table — A. All MLH3 mutagenesis plasmids are derived from pEAI254, a 7.8 kb MLH3SK1::KANMX integrating vector. pEAI254 was mutagenized by QuickChange to create the alleles listed. The DNA sequence of the entire ORF, and 70 bp upstream and 150 bp downstream, were confirmed by DNA sequencing using primers EAO318, EAO319, EAO1778 and EAO321. B. For the two-hybrid analysis, pEAM105 contains the entire MLH1 gene derived from the SK1 strain inserted immediately after the lexA binding domain in pBTM116. All GAL4 activating domain-mlh3 plasmids are derived from pEAM234, which contains DNA sequence encoding SK1 MLH3 amino acids 481 to 715 inserted immediately after the GAL4 activating domain in pGAD424. C. SGS1 was cloned into pRS426 to make pEAM266 as described in the Methods. Site directed mutagenesis of pEAM266 was performed to make pEAM270 carrying the helicase defective sgs1 allele as described in the Methods. (PDF) [file pgen.1006974.s010.pdf]

S3 Table. Plasmids used in this study.

A. *MLH3* mutagenesis plasmids	
	allele
pEAI254	*MLH3*
pEAI325	*mlh3-1*
pEAI326	*mlh3-2*
pEAI327	*mlh3-3*
pEAI328	*mlh3-4*
pEAI329	*mlh3-5*
pEAI330	*mlh3-6*
pEAI331	*mlh3-7*
pEAI332	*mlh3-8*
pEAI333	*mlh3-9*
pEAI334	*mlh3-10*
pEAI335	*mlh3-11*
pEAI336	*mlh3-12*
pEAI337	*mlh3-13*
pEAI338	*mlh3-14*
pEAI339	*mlh3-15*
pEAI340	*mlh3-16*
pEAI341	*mlh3-17*
pEAI342	*mlh3-18*
pEAI343	*mlh3-19*
pEAI344	*mlh3-20*
pEAI345	*mlh3-21*
pEAI346	*mlh3-22*
pEAI347	*mlh3-23*
pEAI348	*mlh3-24*
pEAI349	*mlh3-25*
pEAI350	*mlh3-26*
pEAI351	*mlh3-27*
pEAI352	*mlh3-28*
pEAI353	*mlh3-29*
pEAI354	*mlh3-30*
pEAI355	*mlh3-31*
pEAI356	*mlh3-32*
pEAI357	*mlh3-33*
pEAI358	*mlh3-34*
pEAI359	*mlh3-35*
pEAI360	*mlh3-36*
pEAI361	*mlh3-37*
pEAI362	*mlh3-38*
pEAI363	*mlh3-39*
pEAI364	*mlh3-40*
pEAI365	*mlh3-41*
pEAI366	*mlh3-42*
pEAI367	*mlh3-43*
pEAI368	*mlh3-44*
pEAI369	*mlh3-45*
pEAI370	*mlh3-46*

pEAI371	*mlh3-47*
pEAI372	*mlh3-48*
pEAI373	*mlh3-49*
pEAI374	*mlh3-50*
pEAI375	*mlh3-51*
pEAI376	*mlh3-52*
pEAI377	*mlh3-53*
pEAI378	*mlh3-54*
pEAI379	*mlh3-55*
pEAI380	*mlh3-56*
pEAI394	*mlh3-57*
pEAI395	*mlh3-58*
pEAI396	*mlh3-59*
pEAI397	*mlh3-60*
pEAI252	*mlh3-D523N*

B. Two-hybrid plasmids

	relevant genotype	
pBTM116	*pADH1-lexA*, dummy vector	*2μ, TRP1*
pEAM105	*pADH1-lexA-MLH1$_{SK1}$*	*2μ, TRP1*
pGAD424	*pADH1-GAL4AD*	*2μ, LEU2*
pEAM234	*pADH1-GAL4-AD-MLH3$_{SK1}$*	*2μ, LEU2*
pEAM235	*pADH1-GAL4-AD-mlh3-39*	*2μ, LEU2*
pEAM236	*pADH1-GAL4-AD-mlh3-40*	*2μ, LEU2*
pEAM237	*pADH1-GAL4-AD-mlh3-41*	*2μ, LEU2*
pEAM238	*pADH1-GAL4-AD-mlh3-42*	*2μ, LEU2*
pEAM241	*pADH1-GAL4-AD-mlh3-45*	*2μ, LEU2*
pEAM242	*pADH1-GAL4-AD-mlh3-48*	*2μ, LEU2*
pEAM244	*pADH1-GAL4-AD-mlh3-54*	*2μ, LEU2*
pEAM245	*pADH1-GAL4-AD-mlh3-60*	*2μ, LEU2*

C. 2μ *URA3* plasmids

	Relevant genotype
pRS426 (pEAO34)	Dummy vector
pEAM266	*SGS1*
pEAM270	*sgs1-K706A* (*sgs1-hd*)

A. All *MLH3* mutagenesis plasmids are derived from pEAI254, a 7.8 kb *MLH3$_{SK1}$::KANMX* integrating vector. pEAI254 was mutagenized by QuickChange to create the alleles listed. The DNA sequence of the entire ORF, and 70 bp upstream and 150 bp downstream, were confirmed by DNA sequencing using primers EAO318, EAO319, EAO1778 and EAO321. B. For the two-hybrid analysis, pEAM105 contains the entire *MLH1* gene derived from the SK1 strain inserted immediately after the lexA binding domain in pBTM116. All *GAL4* activating domain-*mlh3* plasmids are derived from pEAM234, which contains DNA sequence encoding SK1 *MLH3* amino acids 481 to 715 inserted immediately after the GAL4 activating domain in pGAD424. C. *SGS1* was cloned into pRS426 to make pEAM266 as described in the Methods. Site directed mutagenesis of pEAM266 was performed to make pEAM270 carrying the *sgs1* helicase defective allele as described in the Methods.
