# Supplementary material for: mlh3 mutations in baker’s yeast alter meiotic recombination outcomes by increasing noncrossover events genome-wide
Source: PLoS Genet. 2017 Aug 21;13(8):e1006974. doi: 10.1371/journal.pgen.1006974 (PMC5578695; doi:10.1371/journal.pgen.1006974)
Supplement: S4 Table — All mlh3 mutants are isogenic derivatives of EAY1112/EAY2413 (S2 Table; Methods). For single spores, recombination frequencies (recombinant spores/total spores) were multiplied by 100 to yield genetic map distances (cM). For tetrads, genetic distance in centimorgans (cM) was calculated using the RANA software without considering aberrant segregants (Argueso et al. [7]). The Stahl Laboratory Online Tools website (http://molbio.uoregon.edu/~fstahl/) was used to calculate standard error (SE) around the genetic distance for tetrads. n; number of single spores, N; four spore viable tetrads analyzed; Par, parental single spores; Rec, recombinant single spores. (PDF) [file pgen.1006974.s011.pdf]

S4 Table. Genetic map distances for *mlh3* separation of function mutants on chromosome XV from single spores and tetrads.

	Single Spores				Tetrads					
**Genotype**	**n**	**Par.**	**Rec.**	**cM**	**N**	**PD**	**TT**	**NPD**	**cM**	**SE**
URA3-LEU2										
MLH3	896	641	255	28.5	205	93	106	4	32.0	3.2
mlh3Δ	1028	876	152	14.8	210	146	61	2	17.5	2.5
mlh3-6	907	691	216	23.8	205	110	84	5	28.6	3.5
mlh3-23	480	391	89	18.5	105	69	32	1	18.6	3.6
mlh3-32	995	836	159	16.0	205	140	61	1	16.6	2.1
mlh3-42	731	551	180	24.6	154	79	73	1	25.8	2.7
mlh3-45	925	709	216	23.4	204	103	99	0	24.5	1.8
mlh3-54	694	533	161	23.2	155	79	70	2	27.2	3.3
LEU2-LYS2										
MLH3	896	680	216	24.1	205	103	99	1	25.9	2.2
mlh3Δ	1028	923	105	10.2	210	166	43	0	10.3	1.4
mlh3-6	907	738	169	18.6	205	129	68	2	20.1	2.6
mlh3-23	480	397	83	17.3	105	69	33	0	16.2	2.3
mlh3-32	995	854	141	14.2	205	144	58	0	14.4	1.6
mlh3-42	731	564	167	22.9	154	81	71	1	25.2	2.7
mlh3-45	925	739	186	20.1	204	123	78	1	20.8	2.2
mlh3-54	694	550	144	20.8	155	90	61	0	20.2	2.0
LYS2-ADE2										
MLH3	896	815	81	9.0	205	168	35	0	8.6	1.3
mlh3Δ	1028	988	40	3.9	210	193	16	0	3.8	0.9
mlh3-6	907	812	95	10.5	205	158	41	0	10.3	1.4
mlh3-23	480	454	26	5.4	105	92	10	0	4.9	1.5
mlh3-32	995	930	65	6.5	205	176	26	0	6.4	1.2
mlh3-42	731	663	68	9.3	154	124	29	0	9.5	1.6
mlh3-45	925	856	69	7.5	204	172	29	1	8.7	1.9
mlh3-54	694	627	67	9.7	155	121	30	0	9.9	1.6
ADE2-HIS3										
MLH3	896	560	336	37.5	205	63	129	11	48.0	4.5
mlh3Δ	1028	829	199	19.4	210	130	79	0	18.9	1.7
mlh3-6	907	665	242	26.7	205	98	95	6	32.9	3.8
mlh3-23	480	352	128	26.7	105	46	55	1	29.9	3.6
mlh3-32	995	811	184	18.5	205	129	70	3	21.8	2.9
mlh3-42	731	491	241	33.0	154	58	89	6	40.8	4.7
mlh3-45	925	650	275	29.7	204	85	116	1	30.2	2.2
mlh3-54	694	469	225	32.4	155	58	88	5	39.1	4.4

All *mlh3* mutants are isogenic derivatives of EAY1112/EAY2413 (S2 Table; Methods). For single spores, recombination frequencies (recombinant spores/total spores) were multiplied by 100 to yield genetic map distances (cM). For tetrads, genetic distance in centimorgans (cM) was calculated using the RANA software without considering aberrant segregants (Argueso et al. [7]). The Stahl Laboratory Online Tools website (http://molbio.uoregon.edu/~fstahl/) was used to calculate standard error (SE) around the genetic distance for tetrads. n; number of single spores, N; four spore viable tetrads analyzed; Par, parental single spores; Rec, recombinant single spores.
