# Supplementary material for: mlh3 mutations in baker’s yeast alter meiotic recombination outcomes by increasing noncrossover events genome-wide
Source: PLoS Genet. 2017 Aug 21;13(8):e1006974. doi: 10.1371/journal.pgen.1006974 (PMC5578695; doi:10.1371/journal.pgen.1006974)
Supplement: S5 Table — (PDF) [file pgen.1006974.s012.pdf]

S5 Table. Sequencing statistics for spores derived from wild type, *mlh3-23* , *mlh3-32, mlh3-D523N* and *mlh3*Δ in S288c/YJM789 hybrid bearing SK1-*MLH1/MLH3* alleles.

Spore ID	SNPs genotyped	Coverage Depth (X)
wt_2a	73593	66.40
wt_2b	74058	79.95
wt_2c	73682	70.88
wt_2d	73565	73.79
wt_5a	71120	31.09
wt_5b	74269	71.53
wt_5c	71985	40.23
wt_5d	71278	39.96
wt_6a	73967	31.05
wt_6b	73838	39.35
wt_6c	73779	40.65
wt_6d	72914	30.12
wt_7a	72106	17.09
wt_7b	70109	10.83
wt_7c	71589	12.98
wt_7d	73637	48.78
wt_8a	73934	26.02
wt_8b	72929	27.20
wt_8c	72853	23.29
wt_8d	72057	20.39
wt_9a	72729	25.17
wt_9b	73773	34.92
wt_9c	73366	29.59
wt_9d	72637	27.90
wt_10a	73326	88.63
wt_10b	73176	25.16
wt_10c	73198	30.37
wt_10d	73612	22.72
wt_11a	73020	40.30
wt_11b	73309	43.98
wt_11c	73553	31.89
wt_11d	73034	37.50
wt_12a	72904	30.86
wt_12b	73006	20.06
wt_12c	74485	29.35
wt_12d	74593	28.81
wt_13a	73772	39.24
wt_13b	73934	46.79
wt_13c	73051	37.72
wt_13d	73012	29.61
mlh3_23_6a	72821	26.00
mlh3_23_6b	73731	30.46
mlh3_23_6c	74242	74.35
mlh3_23_6d	73587	38.45
mlh3_23_7a	73307	31.01
mlh3_23_7b	72910	30.35
mlh3_23_7c	74113	26.86

mlh3_23_7d	73731	33.87
mlh3_23_8a	73652	42.29
mlh3_23_8b	73500	23.72
mlh3_23_8c	73523	35.44
mlh3_23_8d	73019	27.51
mlh3_23_9a	72890	24.92
mlh3_23_9b	72762	39.19
mlh3_23_9c	73009	23.54
mlh3_23_9d	73289	28.28
mlh3_23_10a	74434	34.56
mlh3_23_10b	71249	13.69
mlh3_23_10c	72944	27.91
mlh3_23_10d	73138	36.67
mlh3_23_11a	74791	44.61
mlh3_23_11b	72578	39.03
mlh3_23_11c	73377	30.72
mlh3_23_11d	74015	35.07
mlh3_23_12a	72112	20.22
mlh3_23_12b	72717	19.77
mlh3_23_12c	72680	23.84
mlh3_23_12d	72243	21.08
mlh3_32_6a	73361	36.57
mlh3_32_6b	73183	37.66
mlh3_32_6c	72332	24.60
mlh3_32_6d	71679	22.96
mlh3_32_7a	72832	198.87
mlh3_32_7b	71095	11.60
mlh3_32_7c	73330	31.58
mlh3_32_7d	73238	26.67
mlh3_32_8a	72758	217.13
mlh3_32_8b	70392	29.44
mlh3_32_8c	70315	27.33
mlh3_32_8d	72879	149.71
mlh3_32_9a	74387	98.47
mlh3_32_9b	63895	45.17
mlh3_32_9c	72482	141.67
mlh3_32_9d	73950	204.92
mlh3_32_11a	73202	29.09
mlh3_32_11b	73770	27.74
mlh3_32_11c	72274	34.69
mlh3_32_11d	73102	31.94
mlh3_32_12a	73497	41.42
mlh3_32_12b	72933	31.95
mlh3_32_12c	73648	32.22
mlh3_32_12d	73847	32.89
mlh3_32_13a	73579	34.68
mlh3_32_13b	73554	34.46
mlh3_32_13c	74400	71.42
mlh3_32_13d	72551	20.67
mlh3DN_1a	74093	53.98
mlh3DN_1b	74212	54.27

mlh3DN_1c	74196	52.60
mlh3DN_1d	74018	78.47
mlh3DN_2a	73782	67.29
mlh3DN_2b	73893	98.45
mlh3DN_2c	73947	104.45
mlh3DN_2d	74717	131.11
mlh3DN_3a	74808	91.32
mlh3DN_3b	73523	53.45
mlh3DN_3c	73649	55.91
mlh3DN_3d	73680	51.84
mlh3DN_4a	73994	46.26
mlh3DN_4b	74042	63.36
mlh3DN_4c	74334	85.10
mlh3DN_4d	74398	51.04
mlh3DN_5a	74793	49.82
mlh3DN_5b	74596	60.02
mlh3DN_5c	74230	47.14
mlh3DN_5d	73957	58.53
mlh3DN_6a	74602	57.33
mlh3DN_6b	74329	72.08
mlh3DN_6c	74203	48.69
mlh3DN_6d	74376	77.95
mlh3DN_7a	74205	48.52
mlh3DN_7b	74246	58.80
mlh3DN_7c	74130	60.66
mlh3DN_7d	73308	65.19
mlh3DN_8a	73246	36.71
mlh3DN_8b	74400	55.49
mlh3DN_8c	74116	78.77
mlh3DN_8d	73848	62.79
mlh3DN_9a	74698	64.18
mlh3DN_9b	74851	76.91
mlh3DN_9c	73978	50.63
mlh3DN_9d	73810	66.36
mlh3DN_10a	73932	66.84
mlh3DN_10b	74272	89.49
mlh3DN_10c	74783	91.61
mlh3DN_10d	74421	78.58
mlh3null_1a	72746	23.75
mlh3null_1b	71493	66.08
mlh3null_1c	73461	27.09
mlh3null_1d	73062	36.04
mlh3null_2a	73687	30.53
mlh3null_2b	73350	28.38
mlh3null_2c	70835	70.07
mlh3null_2d	71034	18.25
mlh3null_3a	72707	26.01
mlh3null_3b	73321	29.99
mlh3null_3c	74005	37.19
mlh3null_3d	72088	24.08
mlh3null_4a	74052	32.98

mlh3null_4b	72938	49.10
mlh3null_4c	74692	52.23
mlh3null_4d	73792	32.23
mlh3null_5a	73803	31.86
mlh3null_5b	74048	33.79
mlh3null_5c	72848	33.27
mlh3null_5d	72772	21.49
mlh3null_6a	71196	69.15
mlh3null_6b	73701	38.79
mlh3null_6c	71783	76.77
mlh3null_6d	71928	19.94
mlh3null_7a	73097	36.67
mlh3null_7b	72210	36.42
mlh3null_7c	75003	60.87
mlh3null_7d	73297	35.11
mlh3null_8a	73597	44.31
mlh3null_8b	73050	56.57
mlh3null_8c	73092	40.96
mlh3null_8d	73375	41.63
mlh3null_9a	73689	38.75
mlh3null_9b	72537	41.30
mlh3null_9c	73632	36.13
mlh3null_9d	73622	34.73
mlh3null_10a	74117	34.20
mlh3null_10b	71858	30.54
mlh3null_10c	73608	39.18
mlh3null_10d	73109	36.95
