# Supplementary material for: mlh3 mutations in baker’s yeast alter meiotic recombination outcomes by increasing noncrossover events genome-wide
Source: PLoS Genet. 2017 Aug 21;13(8):e1006974. doi: 10.1371/journal.pgen.1006974 (PMC5578695; doi:10.1371/journal.pgen.1006974)
Supplement: S6 Table — Crossovers are E2+E3 events and noncrossovers are E1 events [34] (S2 File). (PDF) [file pgen.1006974.s013.pdf]

S6 Table. Crossovers (CO) and noncrossovers (NCO) in tetrads of wild-type, *mlh3-23*, *mlh3-32, mlh3-D523N and mlh3Δ.*

Tetrad_ID	CO (E2+E3)	NCO (E1)
wt_2	73	53
wt_5	82	43
wt_6	71	37
wt_7	100	47
wt_8	78	37
wt_9	76	44
wt_10	83	46
wt_11	81	28
wt_12	85	34
wt_13	82	38
mlh3_23_6	92	63
mlh3_23_7	73	53
mlh3_23_8	63	43
mlh3_23_9	84	49
mlh3_23_10	73	55
mlh3_23_11	112	46
mlh3_23_12	70	51
mlh3_32_6	64	51
mlh3_32_7	72	45
mlh3_32_8	58	64
mlh3_32_9	56	64
mlh3_32_11	53	50
mlh3_32_12	73	55
mlh3_32_13	80	61
mlh3DN_1	61	45
mlh3DN_2	51	53
mlh3DN_3	58	49
mlh3DN_4	66	43
mlh3DN_5	58	55
mlh3DN_6	66	58
mlh3DN_7	55	57
mlh3DN_8	64	44
mlh3DN_9	52	46
mlh3DN_10	59	52
mlh3null_1	44	54
mlh3null_2	36	56
mlh3null_3	47	46
mlh3null_4	53	49
mlh3null_5	48	44
mlh3null_6	55	57
mlh3null_7	55	58
mlh3null_8	52	60
mlh3null_9	50	72
mlh3null_10	52	56

Crossovers are E2+E3 events and noncrossovers are E1 events [34].
