# Supplementary material for: mlh3 mutations in baker’s yeast alter meiotic recombination outcomes by increasing noncrossover events genome-wide
Source: PLoS Genet. 2017 Aug 21;13(8):e1006974. doi: 10.1371/journal.pgen.1006974 (PMC5578695; doi:10.1371/journal.pgen.1006974)
Supplement: S7 Table — Crossovers are E2+E3 events and noncrossovers are E1 events [34] (S2 File). (PDF) [file pgen.1006974.s014.pdf]

S7 Table. Average crossovers (CO) and noncrossovers (NCO) per chromosome for WT (wild type), *mlh3-23, mlh3-32, mlh3-D523N* and *mlh3Δ* mutants.

Chromosome	WT	*mlh3-23*	*mlh3-32*	*mlh3-D523N*	*mlh3Δ*
I	1.6	1.4	1.4	1.1	1.7
II	4.3	5.9	5.0	4.7	3.4
III	3.1	3.0	1.7	2.4	1.9
IV	9.5	8.6	8.1	6.1	6.3
V	3.4	4.4	2.9	2.2	2.5
VI	2.3	2.1	1.9	2.0	1.6
VII	7.0	8.0	4.0	5.3	4.4
VIII	4.7	4.1	3.1	2.7	3.1
IX	3.2	2.9	3.0	2.5	2.3
X	5.3	4.7	3.1	3.1	3.3
XI	4.7	5.0	4.0	3.8	3.5
XII	7.3	7.0	5.3	5.2	4.8
XIII	6.5	5.0	6.0	4.3	4.3
XIV	4.6	5.3	4.1	3.7	2.8
XV	7.5	7.0	5.7	4.8	5.1
XVI	6.1	6.6	5.7	5.1	4.2
**Total_CO**	**81.0**	**81.0**	**65.0**	**59.0**	**55.0**

Chromosome	WT	*mlh3-23*	*mlh3-32*	*mlh3-D523N*	*mlh3Δ*
I	1.2	1.3	1.0	1.0	1.4
II	4.0	4.1	4.0	4.1	3.3
III	0.8	0.7	2.1	1.9	0.9
IV	4.5	7.1	7.3	5.9	5.6
V	2.6	3.3	2.3	3.7	2.9
VI	1.3	1.4	1.1	0.8	1.4
VII	4.3	4.0	5.9	4.1	5.9
VIII	1.4	1.4	3.1	2.3	1.8
IX	1.0	1.9	2.0	1.9	1.7
X	1.9	3.6	3.7	3.1	3.5
XI	1.8	2.1	3.3	2.4	2.1
XII	2.5	3.9	5.7	4.1	4.3
XIII	3.3	5.7	3.7	3.2	3.4
XIV	3.2	2.6	3.0	2.9	2.9
XV	3.4	4.9	4.1	6.1	4.8
XVI	3.5	3.4	3.3	2.7	3.3
**Total_NCO**	**41.0**	**51.0**	**56.0**	**50.0**	**49.0**

Crossovers are E2+E3 events and noncrossovers are E1 events [34].
