# Supplementary material for: mlh3 mutations in baker’s yeast alter meiotic recombination outcomes by increasing noncrossover events genome-wide
Source: PLoS Genet. 2017 Aug 21;13(8):e1006974. doi: 10.1371/journal.pgen.1006974 (PMC5578695; doi:10.1371/journal.pgen.1006974)
Supplement: S8 Table — E1, E2, E4 are classified by groupEvents [34]. P-values show statistical significance of difference in median CO and NCO gene conversion tract lengths of wild-type and mlh3 alleles compared to mlh3Δ using the Wilcoxon rank sum test (Methods; S2 File). (PDF) [file pgen.1006974.s015.pdf]

S8 Table. Gene conversion tract lengths in *mlh3Δ*, Wild type, *mlh3-23, mlh3-32* and *mlh3-D523N* mutants.

Strain	E2 median (kb)	*P* value	E1 median (kb)	*P* value	E4 median (kb)	*P* value
mlh3Δ	2.19		1.50		2.28	
Wild type	1.79	0.005	1.46	0.51	3.01	0.37
mlh3-23	1.96	0.18	1.42	0.37	2.03	0.69
mlh3-32	1.84	0.034	1.34	0.045	2.23	0.65
mlh3-D523N	1.96	0.49	1.48	0.43	2.59	0.40

E1, E2, E4 are classified by groupEvents [34]. P-values show statistical significance of difference in median CO and NCO gene conversion tract lengths of wild-type and *mlh3* alleles compared to *mlh3Δ* using the Wilcoxon rank sum test (Methods).
