# Supplementary material for: mlh3 mutations in baker’s yeast alter meiotic recombination outcomes by increasing noncrossover events genome-wide
Source: PLoS Genet. 2017 Aug 21;13(8):e1006974. doi: 10.1371/journal.pgen.1006974 (PMC5578695; doi:10.1371/journal.pgen.1006974)
Supplement: S9 Table — (PDF) [file pgen.1006974.s016.pdf]

S9 Table. Summary of all event categories in wt, *mlh3-23*, *mlh3-32*, *mlh3-D523N* and *mlh3Δ*.

	**WT**	***mlh3-23***	***mlh3-32***	***mlh3-DN***	***mlh3Δ***
Number of tetrads	10.00	7.00	7.00	10.00	10.00
Total E1	407.00	360.00	390.00	502.00	492.00
E1 per tetrad	40.70	51.43	55.71	50.20	49.20
E1 length mean	1821.22	1804.54	1748.45	1811.19	1895.31
E1 length median	1464.50	1415.75	1335.00	1482.50	1501.00
Total E2	728.00	485.00	407.00	519.00	501.00
E2 per tetrad	72.80	69.29	58.14	51.90	50.10
E2 length mean	1418.21	1530.45	1512.03	1570.09	1775.29
E2 length median	1166.00	1146.50	1153.00	1263.00	1433.50
E2 length(>0) median	1795.00	1964.00	1842.00	1962.00	2192.00
Total E3	83.00	82.00	49.00	71.00	51.00
E3 per tetrad	8.30	11.71	7.00	7.10	5.10
E3 length mean	3154.80	3253.14	2565.66	3588.28	3396.47
E3 length median	3069.00	3036.25	2545.50	3163.00	2870.00
E3 track length mean	1254.65	1094.16	800.45	1079.31	1258.72
E3 tract length median	974.00	920.50	709.00	850.25	966.50
E3 GC length mean	669.32	772.35	641.97	741.70	542.78
E3 GC length median	340.50	304.25	189.00	331.00	0.00
E3 gap length mean	1352.59	1520.39	1247.16	1718.63	1474.08
E3 gap length median	870.50	1128.00	972.00	1488.25	1352.00
Total E4	20.00	22.00	19.00	24.00	13.00
E4 per tetrad	2.00	3.14	2.71	2.40	1.30
E4 length mean	3113.55	2924.32	2762.08	2961.08	2514.19
E4 length median	3008.00	2034.50	2227.50	2587.00	2279.50
E4 track length mean	1182.68	1006.43	806.79	810.73	907.46
E4 tract length median	802.00	649.50	661.50	493.50	553.50
E4 gap length mean	572.66	681.32	741.15	968.88	584.50
E4 gap length median	440.75	484.00	520.75	701.50	276.75
Total E5	29.00	39.00	27.00	45.00	34.00
E5 per tetrad	2.90	5.57	3.86	4.50	3.40
E5 length mean	3358.71	3685.97	3389.41	3616.73	3718.44
E5 length median	2438.50	3654.50	3305.00	3682.50	3364.00
Total E5A	17.00	16.00	16.00	23.00	21.00
E5A per tetrad	1.70	2.29	2.29	2.30	2.10
E5A length mean	2550.94	3695.19	3305.63	3260.50	3714.02
E5Alength median	2408.00	3350.50	3211.25	3571.50	3360.00
Total E6	80.00	53.00	40.00	50.00	43.00
E6 per tetrad	8.00	7.57	5.71	5.00	4.30
E6 length mean	3724.04	4055.31	3863.69	3992.64	4050.65
E6 length median	3199.75	3663.00	3368.25	3520.50	3829.00
Total E7	10.00	5.00	3.00	15.00	8.00
E7 per tetrad	1.00	0.71	0.43	1.50	0.80
E7 length mean	3499.25	3163.50	4589.33	3299.13	5029.06
E7 length median	3832.00	2661.00	3392.50	3832.00	4572.75
Total JM	930.00	664.00	526.00	700.00	637.00
Total JM per tetrad	93.00	94.86	75.14	70.00	63.70
Total NCO	427.00	382.00	409.00	526.00	505.00
Total NCO per tetrad	42.70	54.57	58.43	52.60	50.50
Total IH	1357.00	1046.00	935.00	1226.00	1142.00
Total IH per tetrad	135.70	149.43	133.57	122.60	114.20
