# Supplementary material for: Correction: mlh3 mutations in baker’s yeast alter meiotic recombination outcomes by increasing noncrossover events genome-wide
Source: PLoS Genet. 2017 Oct 24;13(10):e1007067. doi: 10.1371/journal.pgen.1007067 (PMC5655472; doi:10.1371/journal.pgen.1007067)
Supplement: S1 File — (PDF) [file pgen.1007067.s001.pdf]

PLOS GENETICS

RESEARCH ARTICLE

mlh3 mutations in baker's yeast alter meiotic recombination outcomes by increasing noncrossover events genome-wide

Najla Al-Sweel[1], Vandana Raghavan[1], Abhishek Dutta[2], V. P. Ajith[2], Luigi Di Vietro[3¤], Nabila Khondakar[1], Carol M. Manhart[1], Jennifer A. Surtees[4], K. T. Nishant[2,5]*, Eric Alani[1]*

1 Department of Molecular Biology and Genetics, Cornell University, Ithaca, New York, United States of America, **2** School of Biology, Indian Institute of Science Education and Research Thiruvananthapuram, Trivandrum, India, **3** Department of Life Sciences and Systems Biology, University of Turin, Via Verdi, Turin, Italy, **4** Department of Biochemistry, University at Buffalo, State University of New York, Buffalo, New York, United States of America, **5** Center for Computation Modelling and Simulation, Indian Institute of Science Education and Research Thiruvananthapuram, Trivandrum, India

¤ Current address: Bayer CropScience, Lyon, France
* eea3@cornell.edu (EA); nishantkt@iisertvm.ac.in (KTN)

Check for updates

OPEN ACCESS

Citation: Al-Sweel N, Raghavan V, Dutta A, Ajith VP, Di Vietro L, Khondakar N, et al. (2017) *mlh3* mutations in baker's yeast alter meiotic recombination outcomes by increasing noncrossover events genome-wide. PLoS Genet 13(8): e1006974. https://doi.org/10.1371/journal.pgen.1006974

Editor: Michael Lichten, National Cancer Institute, UNITED STATES

Received: February 14, 2017

Accepted: August 12, 2017

Published: August 21, 2017

Copyright: © 2017 Al-Sweel et al. This is an open access article distributed under the terms of the Creative Commons Attribution License, which permits unrestricted use, distribution, and reproduction in any medium, provided the original author and source are credited.

Data Availability Statement: All relevant data are within the paper and its Supporting Information files with the exception of the following: The raw recombination data files and the custom R scripts are available online at the Dryad digital repository (http://dx.doi.org/10.5061/dryad.bb702). Sequence data are available from the National Centre for Biotechnology Information Sequence Read Archive (Accession numbers SRP096621 and SRP110341).

Abstract

Mlh1-Mlh3 is an endonuclease hypothesized to act in meiosis to resolve double Holliday junctions into crossovers. It also plays a minor role in eukaryotic DNA mismatch repair (MMR). To understand how Mlh1-Mlh3 functions in both meiosis and MMR, we analyzed in baker's yeast 60 new *mlh3* alleles. Five alleles specifically disrupted MMR, whereas one (*mlh3-32*) specifically disrupted meiotic crossing over. Mlh1-mlh3 representatives for each class were purified and characterized. Both Mlh1-mlh3-32 (MMR$^+$, crossover$^-$) and Mlh1-mlh3-45 (MMR$^-$, crossover$^+$) displayed wild-type endonuclease activities *in vitro*. Msh2-Msh3, an MSH complex that acts with Mlh1-Mlh3 in MMR, stimulated the endonuclease activity of Mlh1-mlh3-32 but not Mlh1-mlh3-45, suggesting that Mlh1-mlh3-45 is defective in MSH interactions. Whole genome recombination maps were constructed for wild-type and MMR$^+$ crossover$^-$, MMR$^-$ crossover$^+$, endonuclease defective and null *mlh3* mutants in an S288c/YJM789 hybrid background. Compared to wild-type, all of the *mlh3* mutants showed increases in the number of noncrossover events, consistent with recombination intermediates being resolved through alternative recombination pathways. Our observations provide a structure-function map for Mlh3 that reveals the importance of protein-protein interactions in regulating Mlh1-Mlh3's enzymatic activity. They also illustrate how defective meiotic components can alter the fate of meiotic recombination intermediates, providing new insights for how meiotic recombination pathways are regulated.

Author summary

During meiosis, diploid germ cells that become eggs or sperm undergo a single round of DNA replication followed by two consecutive chromosomal divisions. The segregation of chromosomes at the first meiotic division is dependent in most organisms on at least one

Funding: NAS, VR, LDV, NK, and EA were supported by NIH GM53085. NAS was also supported by fellowships provided by the Saudi Arabian Cultural Mission and the Harry and Samuel Mann Award. LDV was also supported by internship funds provided by the University of Turin. CMM was supported by NIH training grant F32 GM112435. JAS was supported by NIH GM087549. KTN, AD, and VPA are supported by a Wellcome Trust-Department of Biotechnology (DBT) India Alliance Intermediate fellowship (IA/I/11/2500268) and Indian Institute of Science Education and Research Thiruvananthapuram (IISER-TVM) intramural funds. VPA was also supported by a UGC (University Grants Commission) fellowship from the Government of India. The content is solely the responsibility of the authors and does not necessarily represent the official views of the National Institute of General Medical Sciences or the National Institutes of Health. The funders had no role in study design, data collection and analysis, decision to publish, or preparation of the manuscript.

Competing interests: The authors have declared that no competing interests exist.

genetic exchange, or crossover event, between chromosome homologs. Homologs that do not receive a crossover frequently undergo nondisjunction at the first meiotic division, yielding gametes that lack chromosomes or contain additional copies. Such events have been linked to human disease and infertility. Recent studies suggest that the Mlh1-Mlh3 complex is an endonuclease that resolves recombination intermediates into crossovers. Interestingly, this complex also acts as a matchmaker in DNA mismatch repair (MMR) to remove DNA replication errors. How does one complex act in two different processes? We investigated this question by performing a mutational analysis of the baker's yeast Mlh3 protein. Five mutations were identified that disrupted MMR but not crossing over, and one mutation disrupted crossing over while maintaining MMR. Using a combination of biochemical and genetic analyses to further characterize these mutants we illustrate the importance of protein-protein interactions for Mlh1-Mlh3's activity. Importantly, our data illustrate how defective meiotic components can alter the outcome of meiotic recombination events. They also provide new insights for the basis of infertility syndromes.

Introduction

During mismatch repair (MMR), insertion/deletion and base-base mismatches that form as the result of DNA replication errors are recognized by MutS homolog (MSH) proteins, which in turn recruit MutL homolog (MLH) proteins to form ternary complexes containing mismatched DNA, MSH factors, and MLH factors. These interactions result in the recruitment of downstream excision and resynthesis proteins to remove the error [1]. In *S. cerevisiae* repair of insertion deletion loops greater than one nucleotide in size primarily involves the MSH heterodimer Msh2-Msh3 and the MLH heterodimer Mlh1-Pms1 [1]. The MLH heterodimer Mlh1-Mlh3 has been shown to play a minor role in this process and can partially substitute for Mlh1-Pms1 in Msh2-Msh3-dependent MMR [2–4]. However, Mlh1-Mlh3 has been shown to play a major role in meiotic crossing over [5–8]. Accurate chromosome segregation in Meiosis I in most eukaryotes requires reciprocal exchange of genetic information (crossing over) between homologs [9–12]. Failure to achieve at least one crossover (CO) per homolog pair results in homolog nondisjunction and the formation of aneuploid gametes. Errors in meiotic chromosome segregation are a leading cause of spontaneous miscarriages and birth defects [13].

Yeast Mlh1-Pms1 and its human ortholog MLH1-PMS2 both exhibit an endonuclease activity that is essential for MMR [14–15]. This activity is dependent on the integrity of a highly conserved (DQHA$(X)_2$E$(X)_4$E) metal binding motif also found in Mlh3. Previous work demonstrated that a point mutation within this motif (*mlh3-D523N*) conferred *mlh3*Δ-like defects in MMR and crossing over. These included a mutator phenotype, a decrease in spore viability to 70% (from 97% in wild-type), and a two-fold reduction in genetic map distances [5]. Consistent with these observations, Mlh1-Mlh3 is an endonuclease that nicks circular duplex DNA *in vitro*, and Mlh1-mlh3-D523N is defective in endonuclease activity [16–17].

Approximately 200 double strand breaks (DSBs) are induced throughout the genome in a *S. cerevisiae* cell in meiotic prophase, of which ~90 are repaired to form COs between homologous chromosomes, with the rest repaired to form noncrossovers (NCOs; [18–23]). In this pathway a DSB, which forms on one chromatid of a homologous pair, is resected by 5' to 3' exonucleases, resulting in the formation of 3' single-strand tails on both sides of the DSB (Fig 1). One of these tails invades the other unbroken homolog and is extended and stabilized to

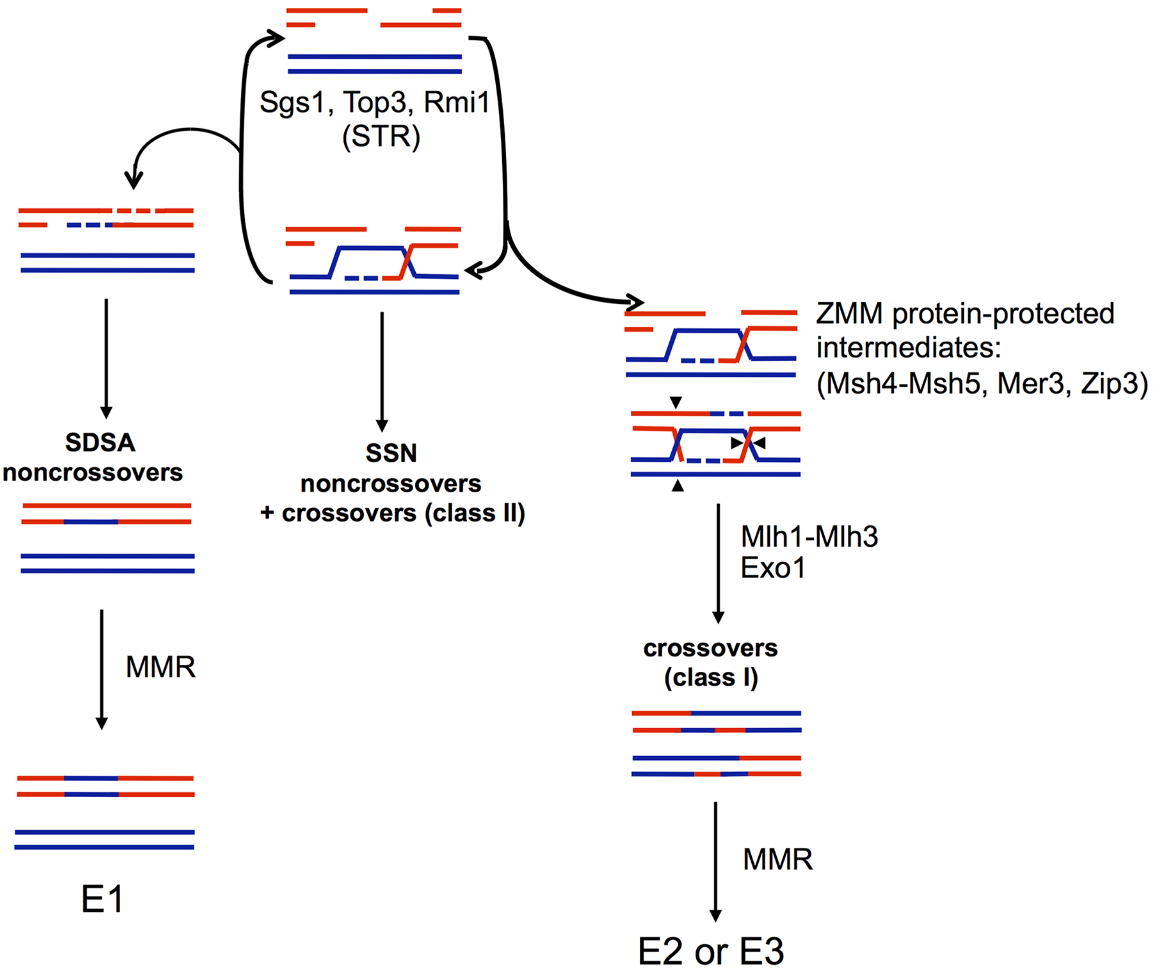

Fig 1. DSB repair pathways in meiosis. Model adapted from Kaur et al. [30] depicting wild-type meiosis and the central role of the STR complex (Sgs1-Top3-Rmi1 helicase/topoisomerase) in disassembling strand invasion intermediates to facilitate synthesis dependent strand annealing (SDSA) or return of events to the original DSB state to allow capture and protection by the ZMM proteins and dHJ formation for ultimate resolution as class I crossovers by Mlh1-Mlh3 and Exo1. Events that escape STR disassembly form unregulated joint molecules that are resolved by the structure selective nucleases (SSNs-Mus81-Mms4, Yen1, Slx-Slx4) as noncrossovers or class II crossovers. The "E" classification of recombination classes was described in Oke et al. [34]. The majority event classes are presented here and result from MMR of heteroduplex DNA intermediates. E1 events are simple noncrossovers (NCO), E2 are simple crossovers (CO) with or without continuous gene conversion, and E3 are COs with discontinuous gene conversion. A set of definitions for these classes can also be found in Fig 5.

https://doi.org/10.1371/journal.pgen.1006974.g001

create a single-end invasion intermediate (SEI). A second invasion event initiating from the SEI, known as second-end capture, can re-anneal and ligate to the other side of the DSB resulting in the formation of a double Holliday junction (dHJ). The dHJ can be acted upon by Holliday junction (HJ) resolvases to form CO and NCO products. In baker's yeast the majority of COs are formed through an interference-dependent CO pathway (class I COs) in which the vast majority of dHJs are resolved to form evenly spaced COs in steps requiring the ZMM proteins Zip1-4, Mer3, and Msh4-Msh5 as well as the Sgs1-Top3-Rmi1 (STR) helicase/topoisomerase complex, Mlh1-Mlh3, and Exo1 [8, 24–31]. These steps are biased to resolve the two junctions present in the dHJ so that the resulting product is exclusively a CO. A second interference-independent pathway was identified that accounts for a small (~10%) number of CO

events (class II COs). In this pathway, which does not involve the ZMM proteins, the two junctions are resolved independently by the Mus81-Mms4 endonuclease, leading to a mixture of CO and NCO products [7, 8, 32, 33].

Genetic and physical studies summarized below support a major role for Mlh1-Mlh3 in promoting meiotic CO formation in the interference-dependent CO pathway. 1. Genetic studies performed in yeast showed that *mlh1* and *mlh3* mutants display approximately two-fold reductions in crossing over [7, 35, 36]. 2. There is significant redundancy of factors required to resolve dHJs into COs. This redundancy involves the endonucleases Mlh1-Mlh3, Mus81-Mms4, Yen1, and Slx1-Slx4 [5, 7, 8], with Yen1 and Slx1-Slx4 acting in cryptic or backup roles. When all four factors were removed, crossing over was reduced to very low levels; however, in an *mms4 slx4 yen1* triple mutant, in which Mlh1-Mlh3 is maintained, relatively high CO levels (~70% of wild-type levels) were observed, suggesting that Mlh1-Mlh3 is the primary factor required for CO resolution in the interference-dependent CO pathway [8]. 3. MLH1 and MLH3 play critical roles in mammalian meiosis [37, 38]. For example, $mlh3^{-/-}$ mice are sterile with an 85–94% reduction in the number of COs; germ cells in these mice fail to maintain homologous pairing at metaphase and undergo apoptosis [37, 39].

Much remains to be understood on how biased resolution of dHJs in the interference-dependent pathway is achieved. A working model, supported by genetic and molecular studies outlined below, is that the STR complex and a subset of ZMM proteins process and interact with DSB repair and SEI intermediates to create a dHJ substrate that can be resolved by the Mlh1-Mlh3 endonuclease and Exo1 to form primarily COs [5–8, 16, 29, 30, 31, 36, 40–46]. In this model, the biased cleavage of a dHJ suggests coordination between the two junctions that would likely require asymmetric loading of meiotic protein complexes at each junction. However, little is known at the mechanistic level about how such coordination could be accomplished. A recent bioinformatics study by the Fung group, which involved the analysis of CO-associated gene conversion patterns in yeast tetrads, suggested that Zip3, a SUMO E3 ligase, is required for biased cleavage [34]. Curiously, they found that biased resolution of dHJs was maintained in *msh4* mutants. Based on these findings and other observations they propose that Msh4-Msh5 is required at the invading end of the DSB to stabilize recombination intermediates such as SEIs, while Zip3 acts to promote second-end capture steps at the ligating end of the DSB [34]. In support of this model, the ZMM heterodimer Msh4-Msh5 has been shown to promote COs in the same pathway as Mlh1-Mlh3, and human MSH4-MSH5 was shown to bind to SEI and Holliday junction substrates *in vitro* [7, 43]. Furthermore, cytological observations in mouse have shown that MSH4-MSH5 foci appear prior to MLH1-MLH3 [37, 44, 47, 48]. Consistent with these observations, MLH1 and MLH3 foci formation requires MSH4-MSH5 [48].

Additional support for the above model was obtained from analysis of the STR complex [8, 30, 31, 46, 49]. The STR complex has recently been labeled the master regulator of meiotic DSB repair, acting as both a positive and negative CO coordinator (Fig 1; [30, 49]). Initially, the Sgs1 helicase was characterized as anti-CO because it facilitates unwinding of DSB repair intermediates to promote NCOs via synthesis-dependent strand annealing (SDSA). However, deleting either Sgs1 or Mlh3 in yeast strains that lack all other meiotic resolvases (*mms4*, *slx4*, *yen1*) results in a similar reduction of CO levels (~10% of wild-type levels) suggesting a pathway where Sgs1-dependent COs require Mlh1-Mlh3 [8]. Similar results were observed in *mms4*, *slx4*, *yen1* strains deficient in Top3 or Rmi1 [30, 31]. These data indicate that the STR complex promotes the majority of COs in conjunction with a resolvase that is not Mus81-Mms4, Slx1-Slx4 or Yen1.

A role for Exo1 in crossing over is supported by genetic studies that show Exo1 and Mlh3 acting in the same CO pathway [29]. Interestingly, Exo1's role in maintaining wild-type levels of crossing over is independent of its catalytic activity, suggesting a structural role for this pro-CO factor [29]. Consistent with the above observations, Msh4-Msh5, STR, Exo1 and Zip3 have all been shown to interact with one another and/or with Mlh1-Mlh3 [50].

In this study, we created a structure-function map of Mlh3 by analyzing 60 new *mlh3* alleles in *S. cerevisiae*. Five alleles predicted to disrupt the Mlh1-Mlh3 endonuclease motif conferred defects in both MMR and crossing over, providing further support that endonuclease activity is required for both functions. Importantly, we identified five *mlh3* mutations that specifically disrupted MMR, and one mutation that specifically disrupted crossing over. By performing biochemical and genetic analyses of the separation of function Mlh1-mlh3 complexes we suggest that the defects seen in our mutants can be explained by a weakening of protein-protein interactions, which can be tolerated in meiosis, but not MMR. Importantly, our high-resolution recombination mapping of these mutants revealed ways in which defective meiotic components can alter the fate of meiotic recombination intermediates.

Results

Rationale for site-directed mutagenesis of *mlh3*

Mlh3 contains a highly conserved N-terminal ATP binding motif, a dynamic and unstructured motif known as the linker arm, and an endonuclease active site that overlaps with a C-terminal Mlh1 interaction domain [51]. We performed a clustered charged-to-alanine scanning mutagenesis of the *S. cerevisiae MLH3* gene to create 60 *mlh3* variants (Fig 2; S1–S3 Tables). Charged residues were considered "clustered" if there were at least two charged residues, consecutive or separated by at most one amino acid, within the primary sequence of Mlh3. Such a directed approach, in the absence of a complete crystal structure, is aimed at targeting the surface of a protein where clusters of charged residues likely reside, while minimizing changes within the interior. In this model, replacement of a charged patch from Mlh3's surface with alanine residues would disrupt protein-protein or protein-DNA interactions without affecting Mlh3 structure. This unbiased mutagenesis has been successfully applied to study the functional domains of several proteins [52, 53], and has provided a comprehensive view of the functional organization of *MLH1* [54]. As shown below, we identified a subset of mutations that caused strong defects in either MMR or crossing over, but not both, likely through disrupted interactions with Mlh1 and other MMR and meiotic CO factors.

Structure-function analysis of Mlh3

We analyzed the effect of *mlh3* mutations on MMR in vegetatively grown cells and on meiotic COs in diploids induced to undergo sporulation. For MMR we employed the *lys2-A*$_{14}$ reversion assay to assess the mutation rate in *mlh3* haploid strains (S1 Table; [55]). In this assay, the median reversion rate of *mlh3Δ* is six-fold higher than wild-type (Fig 3B; S1 Fig; Table 1; [5, 6]). To measure meiotic crossing over we crossed mutant *mlh3* strains to *mlh3Δ* strains (*mlh3* is haplosufficient [6]) to form diploids that were then sporulated (S2 Table). The resulting tetrads were directly visualized for chromosome VIII CO events using a spore autonomous fluorescence assay ([56]; Fig 3A). In *mlh3Δ* strains we observed a more than two-fold decrease in crossing over, as measured by percent tetratype, compared to wild-type (Fig 3B; S1 Fig; Table 1). Similar effects of the *mlh3Δ* mutation on crossing over were seen at other genetic intervals [5–8]. It is important to note that nonparental ditype (NPD) events were not scored because they cannot be distinguished from Meiosis I nondisjunction events [56].

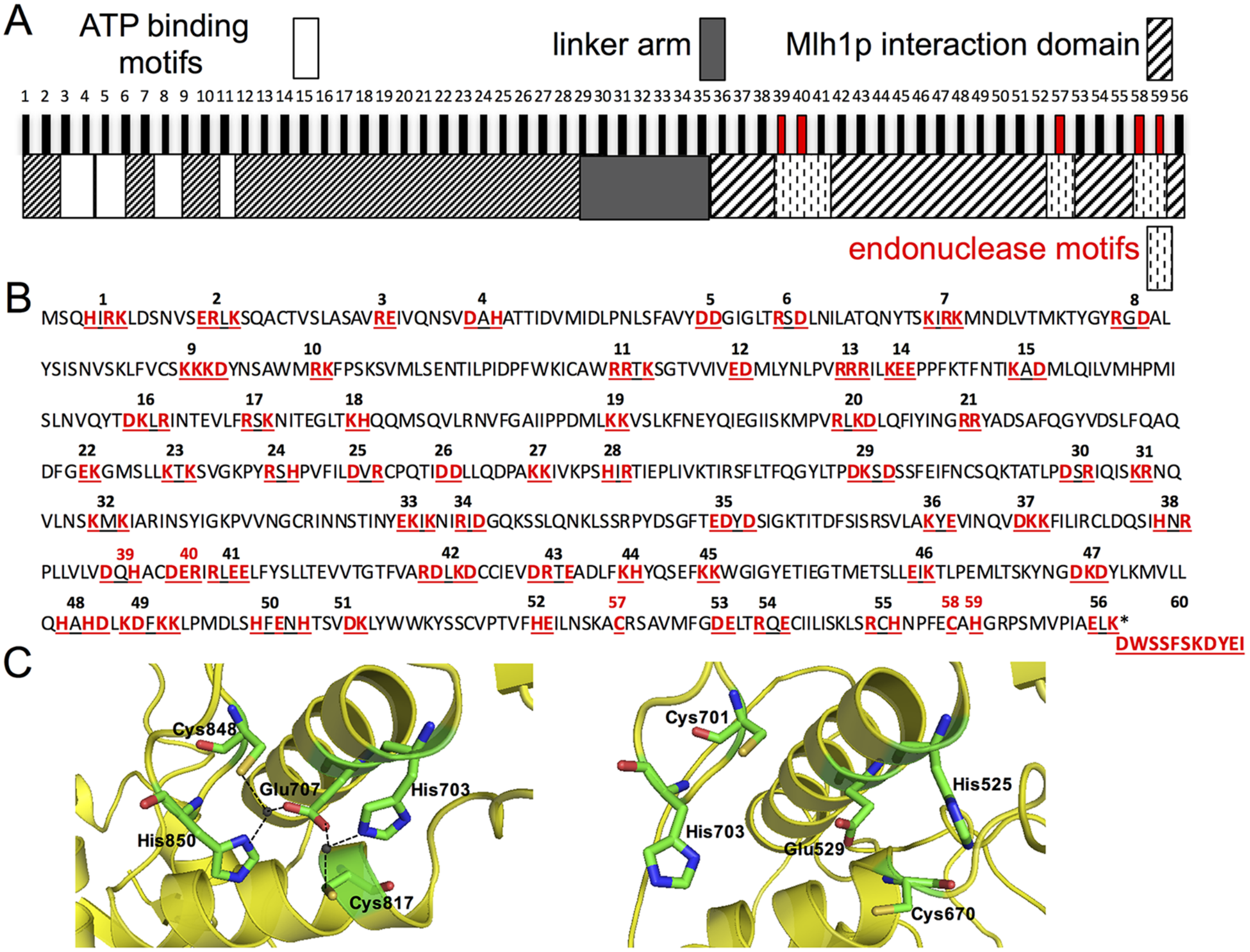

Fig 2. Site directed mutagenesis of *mlh3*. A. Functional organization of Mlh3 based on sequence homology and secondary structure prediction [51]. The vertical bars indicate the approximate position of the *mlh3* mutations (except *mlh3-60*) analyzed in this study and described in panel B. *mlh3-39*, *-40*, *-57*, *-58*, and *-59* colored in red are based on highly conserved residues in the endonuclease motifs of Pms1 which were shown in the crystal structure of Mlh1-Pms1 to form a single metal binding site [51] described in panel C. B. Amino acid positions of charged-to-alanine substitutions presented in red on the primary sequence of *Saccharomyces cerevisiae* Mlh3. Each cluster of underlined residues represents one allele corresponding to the vertical bars in panel A. *mlh3-39*, *-40*, *-57*, *-58*, and *-59* are colored in red as in panel A. *mlh3-60* represents the last 11 residues of Pms1 which constitute patch II of the heterodimerization interface of Mlh1-Pms1 [51]. C. Metal binding site of Pms1 (left panel) from [51] comprised of the five highlighted residues (H703, E707, C817, C848, and H850) were found to be highly conserved in Mlh3 (right panel) based on sequence alignment and structural modeling (H525, E529, C670, C701, and H703) and were targeted in the mutagenesis described in this study (alleles represented in red in A and B).

https://doi.org/10.1371/journal.pgen.1006974.g002

Similar to work performed on a smaller number of *mlh3* alleles and a structure-function analysis of *MLH1*, we found that *mlh3* MMR functions were more sensitive to mutagenesis than CO functions (S1 Fig; [6, 54]). Phenotypes exhibited by *mlh3* strains containing mutations in the ATP-binding motif suggested that this region plays a more critical role in MMR compared to crossing over. However, a region just beyond the ATP-binding domain appeared insensitive to mutagenesis. A null phenotype for both functions was observed in strains bearing mutations in endonuclease motifs, further confirming that endonuclease activity is essential for MMR and crossing over (S1 Fig; Table 1; [5, 8, 16]).

Comparison of the MMR and CO assay results for each individual allele led to the identification of six separation of function mutations, defined as showing strong defects in one

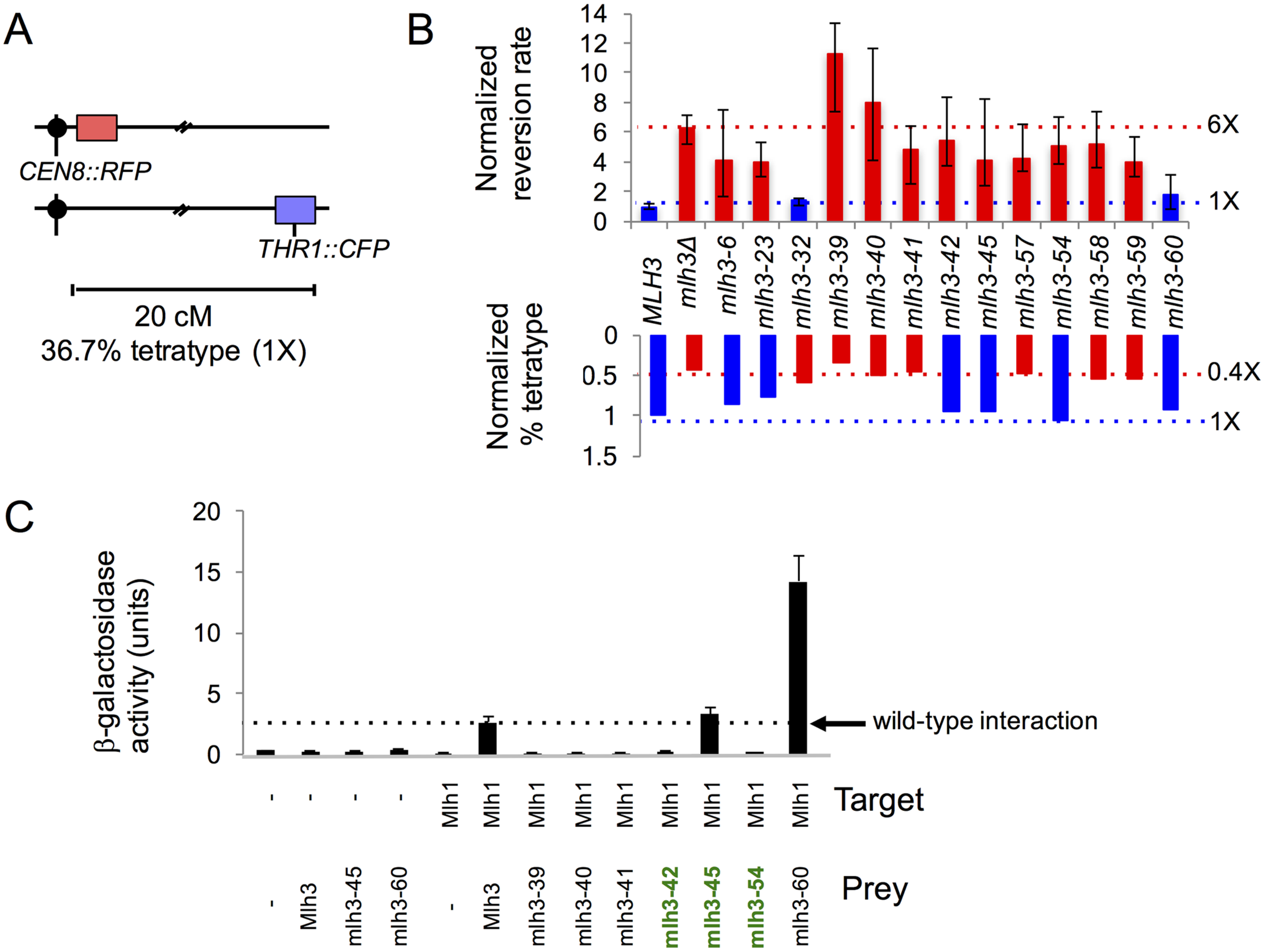

Fig 3. Identification and characterization of *mlh3* separation of function alleles. A. Spore-autonomous fluorescent protein expression was used to quantify crossing over [56]. Shown is the starting parental configuration on chromosome VIII with a map distance of 20 cM separating the red fluorescent protein (RFP) marker and the blue fluorescent protein (CFP) marker. Percent tetratype at this interval in wild-type meiosis is 36.7%. B. MMR (top) and CO (bottom) phenotypes for *mlh3* and *mlh3* null (*mlh3*Δ), separation of function, endonuclease, and C-terminal tail (*mlh3-60*) mutants. Mismatch repair was measured using the *lys2-A$_{14}$* reversion assay [55] and crossing over was measured using the assay depicted in panel A. Bars represent the median reversion rates (error bars based on 95% confidence intervals) and percent tetratype normalized to *mlh3* (1X). For mismatch repair (top), bars represent reversion rates of at least 10 independently tested cultures from two independently constructed strains presented here normalized to *mlh3* median rate of 1X = 1.43x10^{-6} (n = 140). For crossing over (bottom), bars represent percent tetratype of at least 250 tetrads from two independently constructed strains presented here normalized to *mlh3* percent tetratype 1X = 36.7% (n = 226; Table 1 and S2 File). Blue and red dotted lines represent *mlh3* and *mlh3*Δ respectively. C. *mlh3-42*, *-54* weaken Mlh1 interaction yet maintain crossover function. Yeast two-hybrid interactions between lexA-Mlh1 (target) and Gal4-Mlh3 (amino acids 481–715; prey) or Gal4-mlh3-39, -40, -41, -42, -45, -54, -60 derivative constructs, as measured in the ONPG assay for β-galactosidase activity. Error bars indicate standard error of mean from at least three independent assays (S2 File). *mlh3* separation of function alleles indicated in green font.

https://doi.org/10.1371/journal.pgen.1006974.g003

function (e.g. MMR) relative to another (e.g. CO), in the Mlh3 ATP-binding motifs, N-terminal domain beyond the ATP-binding motifs, linker arm, and the interaction domain (Fig 3B; S1 Fig, indicated by stars). One of these alleles (*mlh3-32*) conferred a nearly wild-type phenotype for MMR and a null phenotype for crossing over on chromosome VIII (hereafter referred to as MMR^{+}, CO^{-}). The remaining five mutations (*mlh3-6*, *mlh3-23*, *mlh3-42*, *mlh3-45*, and

Table 1. Mismatch repair (MMR) and crossover (CO) phenotypes of the *mlh3* variants as measured in *lys2-A14* reversion and spore autonomous fluorescent assays.

Allele	Reversion rate (x10^{-6})	95% CI (x10^{-6})	Relative to WT	% tetratype	Relative to WT	Phenotype	
						MMR	CO
mlh3	1.43	1.23–1.65	1.00	36.70	1.00	+	+
mlh3Δ	9.07	7.4–10.28	6.34	16.10	0.44	−	−
mlh3-1	2.72	2.14–3.13	1.90	27.27	0.74	±	+
mlh3-2	2.09	1.63–2.94	1.46	35.69	0.97	+	+
mlh3-3	4.03	3.21–5.39	2.82	33.86	0.92	±	+
mlh3-4	6.89	4.16–8.68	4.82	20.51	0.56	−	−
mlh3-5	7.93	6.79–9.65	5.55	17.30	0.47	−	−
mlh3-6	5.80	2.44–10.7	4.06	31.52	0.86	−	+
mlh3-7	7.46	5.59–10.16	5.22	22.33	0.61	−	−
mlh3-8	3.84	2.77–5.10	2.68	31.37	0.85	±	+
mlh3-9	5.46	3.78–6	3.82	27.02	0.74	±	+
mlh3-10	8.80	7.72–11.98	6.16	18.89	0.51	−	−
mlh3-11	2.49	1.57–2.83	1.74	39.77	1.08	+	+
mlh3-12	8.22	6.2–14.44	5.75	24.00	0.65	−	±
mlh3-13	9.69	6.08–25.7	6.77	24.10	0.66	−	±
mlh3-14	2.64	1.57–3.99	1.85	35.85	0.98	+	+
mlh3-15	8.25	5.84–9.6	5.77	19.85	0.54	−	−
mlh3-16	2.27	1.7–4.78	1.59	32.49	0.89	±	+
mlh3-17	3.62	2.7–6.39	2.53	30.71	0.84	±	+
mlh3-18	4.42	2.74–6.40	3.09	30.51	0.83	±	+
mlh3-19	3.93	3.65–5.23	2.75	23.57	0.64	±	−
mlh3-20	4.12	3.19–6.05	2.88	25.56	0.70	±	±
mlh3-21	3.99	3.41–5.32	2.79	25.81	0.70	±	±
mlh3-22	1.71	1.17–2.96	1.19	35.92	0.98	+	+
mlh3-23	5.69	4.37–7.56	3.98	28.54	0.78	−	+
mlh3-24	4.37	2.57–10.26	3.05	27.31	0.74	−	±
mlh3-25	5.03	4.57–6.22	3.52	37.50	1.02	±	+
mlh3-26	7.54	3.97–12.11	5.28	21.56	0.59	−	−
mlh3-27	5.05	2.75–6.78	3.53	35.60	0.97	±	+
mlh3-28	3.81	2.49–4.51	2.66	34.25	0.93	±	+
mlh3-29	1.91	0.61–4.69	1.33	41.29	1.13	+	+
mlh3-30	2.36	1.7–3.6	1.65	35.00	0.95	±	+
mlh3-31	3.23	2.01–6.91	2.26	37.06	1.01	±	+
mlh3-32	2.00	1.54–2.22	1.40	21.40	0.58	+	−
mlh3-33	1.97	1.65–2.58	1.38	35.49	0.97	+	+
mlh3-34	1.54	1.25–2.82	1.07	38.06	1.04	+	+
mlh3-35	2.26	0.65–3.21	1.58	36.43	0.99	+	+
mlh3-36	3.54	2.37–5.45	2.47	34.66	0.94	±	+
mlh3-37	4.25	2.54–5.15	2.97	22.04	0.60	±	−
mlh3-38	2.26	1.14–3.94	1.58	40.94	1.12	+	+
mlh3-39	16.11	10.54–19	11.26	12.42	0.34	−	−
mlh3-40	11.46	5.81–16.57	8.02	18.45	0.50	−	−
mlh3-41	6.94	3.56–9.1	4.85	17.11	0.47	−	−
mlh3-42	7.70	5.38–12	5.39	35.20	0.96	−	+
mlh3-43	5.31	4.09–7.23	3.71	26.61	0.73	±	+

(*Continued*)

Table 1. (*Continued*)

Allele	Reversion rate (x10^{-6})	95% CI (x10^{-6})	Relative to WT	% tetratype	Relative to WT	Phenotype	
						MMR	CO
mlh3-44	3.59	2.87–4.25	2.51	29.65	0.81	±	+
mlh3-45	5.92	3.49–11.7	4.14	34.79	0.95	−	+
mlh3-46	2.38	1.75–3.01	1.66	37.27	1.02	±	+
mlh3-47	4.10	2.98–5.29	2.87	27.72	0.76	±	+
mlh3-48	5.80	4.01–8.93	4.06	15.41	0.42	−	−
mlh3-49	4.24	3.6–9.72	2.97	16.98	0.46	−	−
mlh3-50	2.75	2.24–3.33	1.92	41.26	1.12	±	+
mlh3-51	1.83	1.04–3.23	1.28	40.84	1.11	+	+
mlh3-52	1.17	0.79–2.7	0.82	35.04	0.95	+	+
mlh3-57	6.07	4.74–9.4	4.25	17.60	0.48	−	−
mlh3-53	3.74	2.52–6.9	2.62	35.61	0.97	±	+
mlh3-54	7.34	5.59–9.97	5.13	38.93	1.06	−	+
mlh3-55	3.72	2.22–5.45	2.60	32.41	0.88	±	+
mlh3-58	7.45	5.23–10.63	5.21	20.00	0.54	−	−
mlh3-59	5.71	4.28–8.19	3.99	20.31	0.55	−	−
mlh3-56	4.65	3.53–6.11	3.25	33.09	0.90	±	+
mlh3-60	2.50	1.18–4.45	1.75	33.83	0.92	+	+

Two independently constructed strains with *mlh3* variants were analyzed in the EAY3255 background which contains the *lys2*::*insE-A*$_{14}$ for MMR testing and the red fluorescent protein for meiotic testing. Haploid strains were examined for reversion to Lys$^+$. At least n = 10 reversion assays were performed per allele. Median reversion rates are presented with 95% confidence intervals (CI), and relative reversion rates compared with the wild-type strain are shown. The haploid strains were mated to EAY3486, which contains the blue fluorescent protein to make diploids suitable for meiotic testing. Diploid strains were induced for meiosis and % tetratype was measured. At least 250 tetrads were counted for each allele (S2 File). WT, wild-type. +, indistinguishable from WT as measured by 95% CI (for reversion rates) or $\chi 2$ (p<0.01, for % tetratype). -, indistinguishable from null as measured by 95% CI or $\chi 2$ (p<0.01). +/-, distinguishable from both wild-type and null as measured by 95% CI or $\chi 2$ (p<0.01).

https://doi.org/10.1371/journal.pgen.1006974.t001

mlh3-54) conferred null MMR phenotypes and nearly wild-type levels of crossing over (hereafter referred to as MMR$^-$, CO$^+$). As indicated below, *mlh3-23* displayed different CO phenotypes in different strain backgrounds, and so its designation as a separation of function allele is less clear.

The phenotypes observed in the separation of function mutants may result from a defect in DNA binding/substrate specificity, endonuclease activity, interactions with specific MMR and meiotic CO factors, or changes in protein conformation. It is important to note that a co-crystal structure of the N-terminal domain of *E*. *coli* MutL (LN40) and *E*. *coli* MutS was recently solved. This work showed that conformational changes license MutS-MutL interaction and are essential for MMR [57, 58].

The endonuclease active sites in Mlh3 and Pms1 appear to be similar

S. *cerevisiae* Mlh1-Pms1 and Mlh1-Mlh3 and human MLH1-PMS2 display latent endonuclease activities dependent on the integrity of a highly conserved metal binding motif DQHA$(X)_2$E$(X)_4$E [14–17]. This motif is critical for Mlh3's MMR and meiotic functions [5]. Two additional motifs were implicated in MLH family endonuclease function based on sequence alignment: ACR and C(P/N)HGRP [59]. In the Mlh1-Pms1 C-terminal domains crystal structure, five Pms1 residues, located in the three conserved motifs (H703, E707, C817, C848, H850), form a metal binding site through folding of the Pms1 C-terminal domain (Fig 2C; [51]). This

organization was also seen in the crystal structure of the C-terminal domain of *B. subtilis* endonuclease MutL (all but H703 are conserved; [60]).

We performed a sequence alignment of Mlh3, Pms1, and *B. subtilis* MutL and found all three possess conserved metal binding motifs, with the following five residues predicted to form the endonuclease active site in Mlh3: H525, E529, C670, C701, and H703. In addition, we constructed a homology model of *S. cerevisiae* Mlh3, and found the C-terminal domain can potentially fold in a similar manner to Pms1 such that these five conserved residues form a single putative metal binding site (Fig 2C). These residues were targeted for site directed mutagenesis of *mlh3* (Fig 2B, shown in red). As shown in S1 Fig (represented in the C-terminal domain by dotted white squares) and Fig 3B and Table 1, mutations in the putative conserved metal binding motifs of Mlh3 (*mlh3-39(D523A, H525A)*, *-40 (D528A, E529A, R530A)*, *-57(C670A)*, *-58(C701A)*, *and -59(H703A)*) conferred null phenotypes for MMR and crossing over, indicating they are essential for Mlh3 function. Thus, these genetic data, combined with the high sequence homology, suggest that H525, E529, C670, C701, and H703 in the C-terminal domain of Mlh3 form the catalytic active site (Fig 2C).

The endonuclease motifs in Mlh3 overlap with the C-terminal Mlh1 interaction domain. To determine if mutations in the Mlh3 endonuclease motifs disrupted interaction with Mlh1, three alleles spanning the DQHA$(X)_2$E$(X)_4$E endonuclease motif (*mlh3-39*, *-40*, *and -41*) were analyzed by yeast two-hybrid for interaction with Mlh1. We also tested these alleles because a previously characterized mutation in the DQHA$(X)_2$E$(X)_4$E endonuclease motif (*mlh3-E529K*) disrupts Mlh1-Mlh3 interactions [5]. As shown in Fig 3C, these mutations disrupted Mlh1-Mlh3 interactions, possibly by altering the endonuclease active site structure. This idea is supported by the Mlh1-Pms1 crystal structure. In this model, heterodimer stability is maintained through interactions between the C-terminal domain of Mlh1 and the endonuclease active site of Pms1 [51]. We cannot rule out the possibility that the null phenotypes observed for MMR and crossing over in *mlh3-39*, *-40*, *and -41* were caused by specifically mutating residues that comprise the Mlh1-Mlh3 dimerization interface without causing a gross disruption in protein folding.

The Mlh1-Pms1 C-terminal domain structure reveals three patches constituting the heterodimerization interface of Mlh1-Pms1 [51]. Patch I is a pseudosymmetric hydrophobic core, Patch II is composed of the last 12 residues of Pms1 and contributes two salt bridges, and Patch III involves the C-terminus of Mlh1 and contributes to the Pms1 metal binding site [51]. Patches I and III are likely maintained in the Mlh1-Mlh3 heterodimerization interface, but Mlh3 lacks the last 11 residues that comprise the bulk of Patch II. This finding gives a likely explanation for partial disruption of the Mlh1-Mlh3 complex when we attempted to analyze it further by gel-filtration [16]. We hypothesized that restoring Patch II to the Mlh1-Mlh3 interaction interface will strengthen this interaction. We engineered a fusion construct of Mlh3 carrying the last 11 residues of Pms1 (*mlh3-60*, Fig 2B in red). As shown in Fig 3C, when we inserted the last 11 residues of Pms1 after the C-terminal residue of Mlh3, we observed a striking increase in the strength of the interaction between Mlh1 and Mlh3 as measured in the yeast two-hybrid assay (2.6 ± 0.5 Miller units of β-galactosidase activity for wild-type Mlh1-Mlh3 compared to 14.2 ± 2.1 for Mlh1-mlh3-60). We initially hypothesized that such an enhanced interaction would be detrimental to MMR because it would sequester Mlh1 from the major MMR endonuclease Pms1. Surprisingly we did not observe a significant effect of the *mlh3-60* mutation on MMR or on crossing over (Fig 3B; Table 1), suggesting that strengthening the interaction between Mlh1-Mlh3 does not affect formation of the Mlh1-Pms1 heterodimer.

mlh3-42 and *mlh3-54* weaken interaction with Mlh1 yet maintain wild-type levels of crossing over

Three of the five MMR$^-$, CO$^+$ alleles (*mlh3-42*, *-45*, and *-54*) contained mutations that mapped to the Mlh1 interaction interface. We performed a two-hybrid assay to test whether these mutations affected Mlh1-Mlh3 dimerization. The *mlh3-45* mutation did not alter Mlh1-Mlh3 interactions; however, both the *mlh3-42* and *mlh3-54* mutations disrupted this interaction (Fig 3C). While such a result could explain the null MMR phenotype conferred by *mlh3-42* and *mlh3-54*, it does not explain why these strains are functional for meiotic crossing over. One explanation is that additional pro-CO factors act as structural scaffolds to stabilize the weakened Mlh1-mlh3 heterodimer, thus allowing it to perform its function at dHJs. Several observations support this idea: 1. The pro-CO factors Msh4-Msh5, Sgs1-Top3-Rmi1, Zip3, and Exo1 interact with one another and/or with Mlh1-Mlh3 [29, 40, 41, 42, 61, 62]. 2. Studies in mice showed that MLH1 and MLH3 do not form a complex until mid to late pachytene; at early to mid pachytene, only MLH3 foci are seen [37, 44]. 3. Exo1's role in crossing over is independent of its enzymatic activity; it is suggested to play a structural role, acting as a platform for pro-CO factors [29]. Together these observations support the presence of a resolvase complex at CO sites that regulates the endonuclease activity of Mlh1-Mlh3 (see Discussion). Alternatively, a weak Mlh1-Mlh3 interaction defect is sufficient to inhibit a yeast-two hybrid interaction, but not affect meiotic recombination if the strength of the Mlh1-Mlh3 interaction is not a limiting factor for CO resolution.

Cumulative genetic distance and spore viability measurements confirm fluorescent assay results for *mlh3* separation of function alleles

The meiotic CO phenotype for the separation of function mutants was determined at four consecutive intervals in chromosome (XV) using traditional genetic map distance analyses (Fig 4A; [5–7]). The overall effect of *mlh3* mutations in crossing over on chromosome XV was similar to that determined on chromosome VIII (Figs 3B and 4B; Table 1; S4 Table). *mlh3-6*, *mlh3-42*, *mlh3-45*, and *mlh3-54* appear similar to wild-type; *mlh3-23* displays an intermediate phenotype, and *mlh3-32* appears similar to the null (Fig 4B; S4 Table). Thus, these mutants, phenotypes of which are summarized in Table 2, were confirmed as separation of function alleles, and are candidates for in-depth characterization and high-resolution recombination mapping.

It is important to note that the spore viability and genetic distance measurements of the six separation of function alleles indicated that CO levels, represented by genetic distance measurements in Chr. XV, can be reduced from 115 cM to ~70 cM without compromising spore viability (Fig 4C; Table 2). A similar observation was made with *msh4/5* hypomorph alleles where crossing over in the four same intervals in Chr. XV could be reduced to ~50 cM without affecting spore viability [63–64]. Together, these observations and the high resolution mapping below indicate that the baker's yeast meiotic cell does not require the full amount of COs maintained by CO homeostasis (~90; see [65]) for accurate chromosome segregation and to form viable spores.

High-resolution recombination maps illustrate unexpected effects of *mlh3* mutants on resolving meiotic recombination intermediates

We characterized three *mlh3* alleles (*mlh3-23*-MMR$^-$, CO$^+$, *mlh3-32*-MMR$^+$, CO$^-$, *mlh3-D523N*) in a genome-wide meiotic recombination assay [66]. *mlh3-23* and *mlh3-32* were chosen as they confer stronger defects in one function relative to the other. The *mlh3-D523N* endonuclease mutation contains an aspartic acid to asparagine substitution in the DQHA(X)$_2$E(X)$_4$E metal

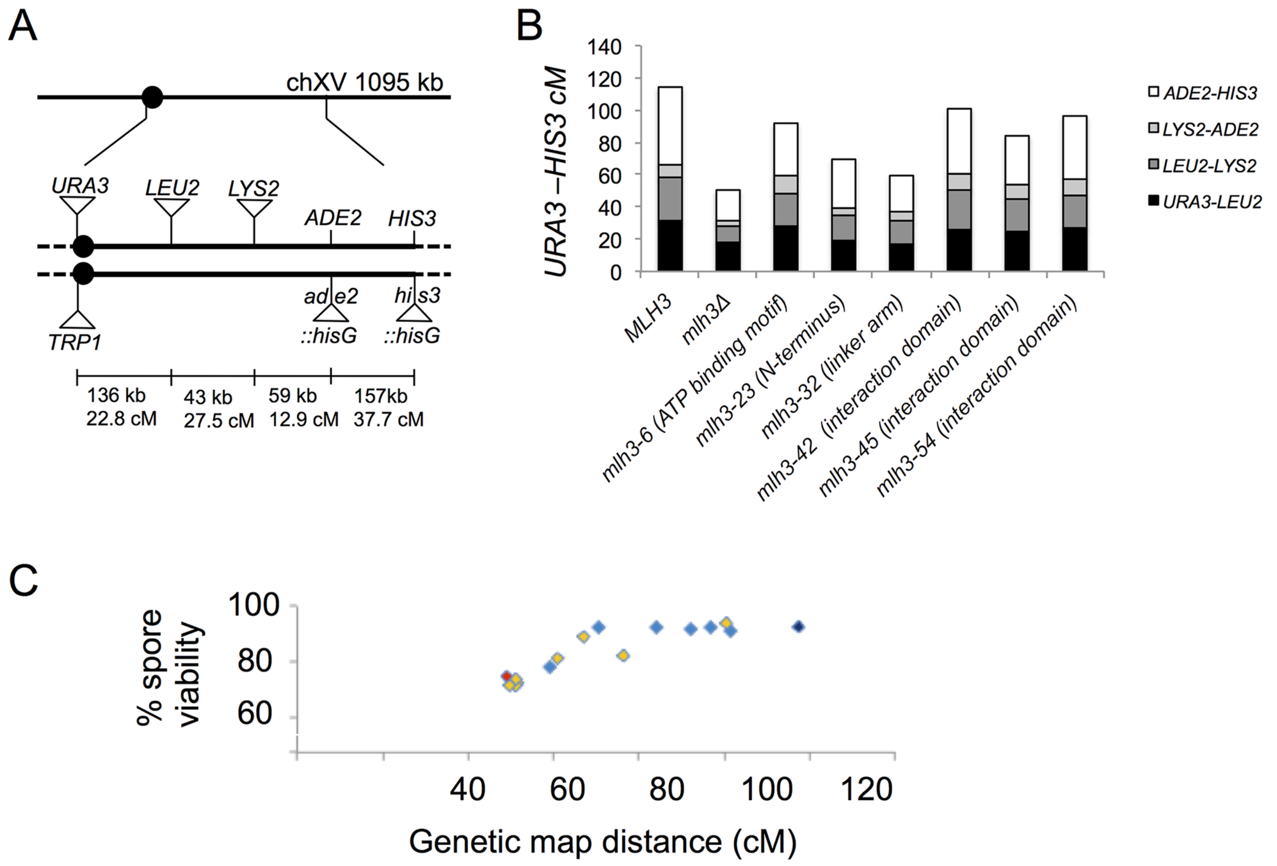

Fig 4. Cumulative genetic distance and spore viability of *mlh3* separation of function mutants. A. Distribution of genetic markers on chromosome XV used to determine genetic distances in the EAY1112/EAY2413 background (S1 Table). The solid circle indicates the centromere. The distances between markers are not drawn to scale. The actual physical and genetic distances in the wild-type diploid are given numerically for each interval and for the entire region between *CENXV* and *HIS3* [7]. B. Cumulative genetic distances between *URA3* and *HIS3* markers from tetrads of *mlh3* and indicated *mlh3* variants. Each bar is further divided into sectors that correspond to the four genetic intervals that span *URA3-HIS3* (S4 Table). C. Spore viabilities are plotted vs. genetic map distances from panel B for *mlh3* (dark blue), *mlh3Δ* (red), and the separation of function mutants (light blue). Yellow diamonds represent data from Sonntag Brown et al. [6] (S2 File).

https://doi.org/10.1371/journal.pgen.1006974.g004

Table 2. Summary of *mlh3* separation of function phenotypes.

Genotype	%SV	Genetic map distance (cM)	% tetratype	Reversion rate X10^{-6} (95%CI)	β-gal units (±SEM)
mlh3	93.3	114.5	36.7	1.4 (1.2–1.7)	2.6 (±0.5)
mlh3Δ	74.9	50.5	16.1	9.1 (7.4–10.3)	ND
mlh3-6	91.4	91.9	31.5	5.8 (2.4–10.7)	ND
mlh3-23	90.9	69.6	28.5	5.7 (4.4–7.6)	ND
mlh3-32	78.5	59.2	21.4	2.0 (1.5–2.2)	ND
mlh3-42	91.8	101.3	35.2	7.7 (5.4–12.0)	0.20 (±0.04)
mlh3-45	92.9	84.2	34.8	5.9 (3.5–11.7)	3.3 (±0.6)
mlh3-54	92.3	96.4	38.9	7.3 (5.6–10.0)	0.16 (±0.03)

Data were obtained from Table 1 (% tetratype, reversion rate), S4 Table (% spore viability (SV), genetic distance in cM), Fig 3C and S2 File (β-gal units in the two-hybrid assay).

ND, not determined.

https://doi.org/10.1371/journal.pgen.1006974.t002

binding motif of Mlh3. This mutation does not disrupt formation of the Mlh1-Mlh3 complex; however, it conferred a null phenotype for *mlh3* functions in MMR and meiotic CO assays, and the Mlh1-mlh3-D523N complex is defective for endonuclease activity [5, 16].

The SK1 *mlh3*, *mlh3Δ*, *mlh3-23*, *mlh3-32*, and *mlh3-D523N* alleles were analyzed in the S288c/YJM789 hybrid background (Fig 5A; S1 and S2 Tables; Methods; [64, 66]). To avoid genetic incompatibilities between Mlh1 and Mlh3, we analyzed SK1 *mlh3*, SK1 *mlh3* mutations, and *mlh3Δ* in the presence of SK1 *MLH1* (Methods). The spore viabilities of *mlh3-23* (84%), *mlh3-32* (82%), and *mlh3-D523N* (82%) were similar to *mlh3Δ* (80%) and the wild-type hybrid containing *SK1-MLH1/3* (82%; Table 3). Why do wild-type and *mlh3* strains show similar viability in the S288c/YJM789 hybrid? *mlh3Δ* mutants display a range of spore viabilities (70 to 92%) that appear to depend on strain background [5, 6, 67]. This is likely to be a partial explanation; however, another study suggested that sequence divergence present in the hybrid strains can affect spore viability through mismatch repair or rejection mechanisms that act on heteroduplex DNA formed during genetic recombination [68].

Seven four-viable spore tetrads each of *mlh3-23* and *mlh3-32*, and ten four-viable spore tetrads each of *mlh3-D523N*, *mlh3Δ*, and *mlh3* were sequenced (S5 Table). The sequence data for all the above *mlh3* point and null mutants along with the control strain are available from the National Centre for Biotechnology Information Sequence Read Archive under the accession numbers SRP096621 and SRP110341. The segregation of the SNPs in all 44 tetrads is shown in S1 File. The CO and NCO counts for all tetrads and the average CO and NCO counts per chromosome are shown in S6 and S7 Tables, respectively.

As described below, high-resolution recombination mapping analysis using groupEvents [34] revealed overall patterns of average CO and NCO events that grouped *mlh3-23* close to wild-type, and *mlh3-D523N* and *mlh3-32* close to *mlh3Δ* (Fig 5). Importantly, this analysis showed that *mlh3* point and null mutants displayed, compared to wild-type, genome-wide increases in NCO events. We did not obtain any evidence that the number of DSBs increased in *mlh3* point mutants based on measuring the total number of interhomolog (IH) events (Fig 6A); such an increase would have provided a simple explanation for why an increase in NCO events was observed, though it is important to note that small changes in total events are challenging to interpret, partly because NCO events can escape detection due to a lack of a polymorphic marker, and we do not have a good estimation of how many such invisible events exist, or how they could be altered by differences in conversion tract length. Thus, we recognize that analyzing total events does not provide a particularly good proxy for DSB (e.g. [69]) levels. Together, these data provide evidence for *mlh3* mutants altering the resolution of meiotic recombination intermediates (see Discussion).

***mlh3-23*, *mlh3-32*, and *mlh3-D523N* display distinct CO phenotypes.** We used groupEvents ([34]; 5 kb threshold for merging nearby events) to categorize all IH events, focusing primarily on the major CO (E2, E3) and NCO events (E1; Figs 1 and 5B), but also examining minority NCO (E4) events that can provide information on whether events resulted from defective mismatch repair or multiple invasion intermediates (Figs 5 and 6; S3 Fig; Table 3; S9 Table; Methods).

Wild-type and *mlh3-23* displayed the same average number of CO events (E2+E3) ($p = 0.99$), and *mlh3Δ*, *mlh3-D523N*, and *mlh3-32* displayed CO levels lower than wild-type ($p < 0.01$). *mlh3-D523N* and *mlh3Δ* displayed similar average CO levels ($p = 0.23$), but *mlh3-32* displayed levels higher than *mlh3Δ* ($p = 0.05$) (Fig 5C and Table 3). An analysis of average CO counts per chromosome was consistent with these observations; *mlh3-23* had a CO distribution similar to wild-type, and *mlh3-32* and *mlh3-D523N* had distributions similar to *mlh3Δ* (S2 Fig). Significant reduction in crossovers were observed primarily on medium (0.5 to 0.9 Mb-II, V, VIII, X, XI, XIV) and large (>0.9 Mb-IV, VII, XII, XIII, XV, XVI) chromosomes in

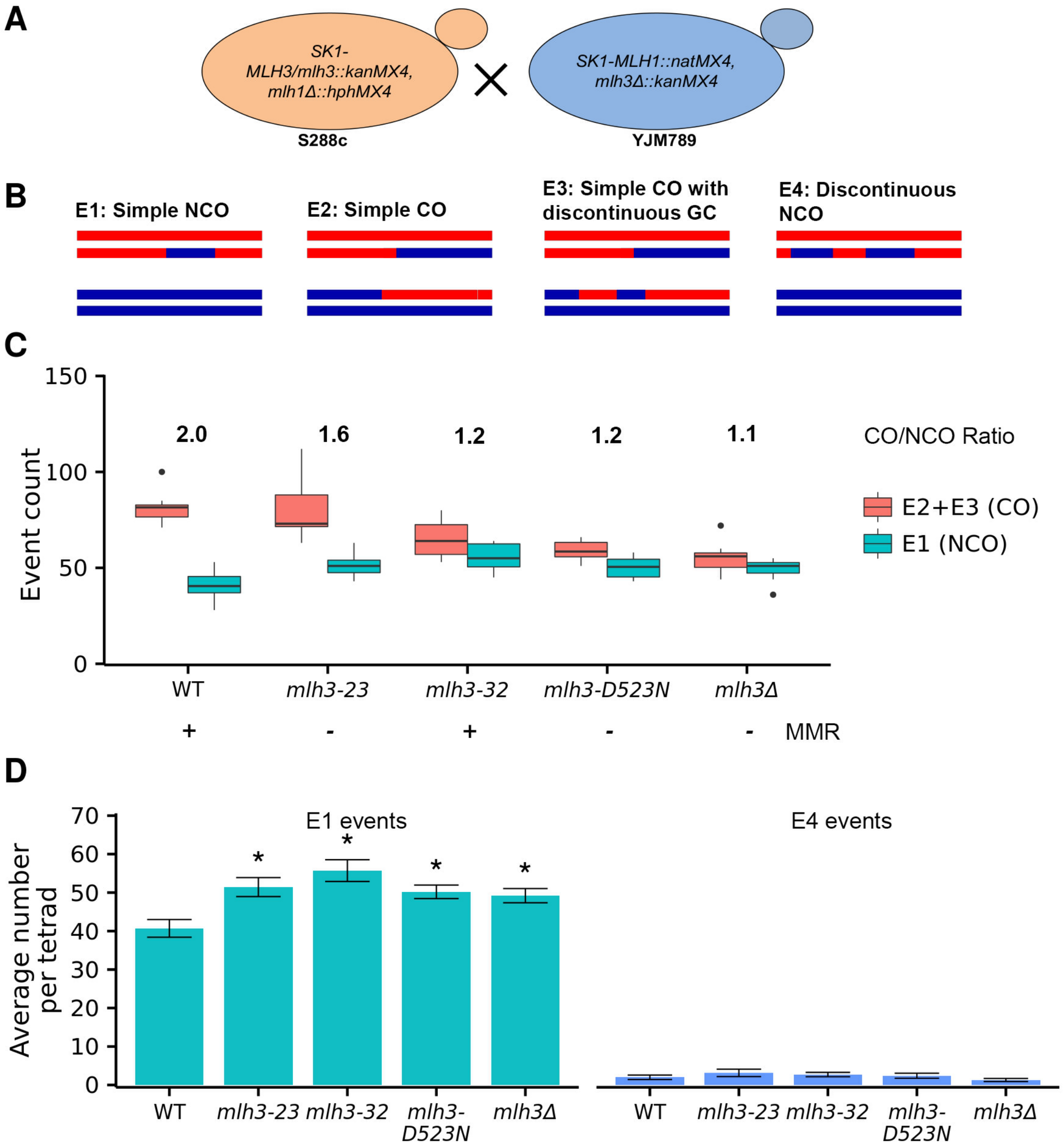

Fig 5. Genome-wide increase in simple noncrossover events (E1) compared to wild-type in *mlh3-23, mlh3-32, mlh3-D523N* and *mlh3Δ* mutants. A. Generation of S288c/YJM789 isogenic strains with SK1 *MLH1*, *mlh3* and the *mlh3-23*, *mlh3-32*, *mlh3-D523N* and *mlh3Δ* mutant alleles (Methods). B. Cartoon description of simple NCO (E1; 3:1 tract on one chromatid, not within 5 kb of another CO or NCO), simple CO (E2; CO with or without an associated gene conversion (GC) tract, and not within 5 kb of another CO or NCO), simple CO with discontinuous gene conversion tracts (E3; same definition as for E2, except with one or more gene conversions within 5 kb and on one of the same chromatids as the CO chromatid), and discontinuous NCOs (E4; two or more NCOs consecutively on one chromatid, with 2:2 marker segregation separating them) as presented in Oke *et al*. [34]. C. Crossover (CO, E2+E3) and noncrossover (NCO, E1) counts per meiosis for wild-type, *mlh3-23*, *mlh3-32*, *mlh3-D523N*, and *mlh3Δ*. The minimum, first quantile, median, third quantile and maximum count are indicated in the box plot. The ratio of CO to NCO events is presented above the box plots. The proficiency of the *mlh3* alleles in mismatch repair is shown as +, MMR proficient, or –, MMR deficient. D. Average number of simple NCO (E1) and NCO with discontinuous tract (E4) events per tetrad (+/- standard error). * $p \leq 0.05$ compared to wild-type (Table 3 and S2 File).

https://doi.org/10.1371/journal.pgen.1006974.g005

Table 3. Spore viability, crossover (CO) and noncrossover (NCO) values for *mlh3-23*, *mlh3-32*, *mlh3-D523N*, and *mlh3Δ* mutants in the S288c/YJM789 hybrid.

Genotype: S288c x YJM789 with SK1-*MLH1*	N	% SV	Tetrads genotyped	Avg. CO (E2 + E3) ± SEM (Median)	Avg. NCO (E1) ± SEM (Median)	Avg. NCO (E4) ± SEM (Median)
SK1-*mlh3*	168	82	10	81±3 (82)	41±2 (41)	2.0±0.6 (1.0)
SK1-*mlh3-23*	80	84	7	81±6 (73)	51±3 (51)	3.1±0.9 (3.0)
SK1-*mlh3-32*	80	82	7	65±4 (64)	56±3 (55)	2.7±0.6 (3.0)
SK1-*mlh3-D523N*	88	82	10	59±2 (59)	50±2 (51)	2.4±0.7 (2.0)
mlh3Δ	80	80	10	55±3 (56)	49±2 (51)	1.3±0.4 (1.5)

N = number of tetrads dissected for measuring spore viability (SV).

The E classes were all analyzed in groupEvents. E2 +E3 = total crossovers;

E1 = simple noncrossover and E4 = discontinuous noncrossover. SEM, standard error of the mean (S2 File).

https://doi.org/10.1371/journal.pgen.1006974.t003

mlh3Δ (III, VIII, X, XIV, XIII, XVI, XII, VII, XV, IV), *mlh3-D523N* (VIII, X, XIII, VII, XV, IV) and *mlh3-32* (X, VII; S2B Fig; [70]). Together, these observations are consistent with the analysis of map distances at specific loci, though the *mlh3-23* mutant displayed an improved CO phenotype in the S288c/YJM789 background compared to the SK1 background (Figs 3, 4 and 5C).

The median gene conversion tract lengths associated with COs (E2 events; Fig 6B; S3 Fig; S8 Table) were significantly longer ($p \leq 0.03$) for *mlh3Δ* (2.19 kb) compared to *mlh3* (1.79 kb) and *mlh3-32* (1.84 kb), but were not significantly longer ($p \geq 0.18$) for *mlh3-23* (1.96 kb), and *mlh3-D523N* (1.96 kb). We found this interesting because *mlh3* and *mlh3-32* mutants appeared similarly functional for MMR, whereas *mlh3Δ*, *mlh3-23*, and *mlh3-D523N* all displayed MMR defects (Table 1). These observations encouraged us to test if Mlh1-Mlh3 MMR functions affected the processing of gene conversion tracts (see below and Discussion).

Genome-wide increase in noncrossovers in *mlh3-23*, *mlh3-32*, *mlh3-D523N* and *mlh3Δ* mutants. The average numbers of NCOs (E1 events) in *mlh3Δ* (49), *mlh3-23* (51), *mlh3-32* (56), and *mlh3-D523N* (50) were significantly higher than the wild-type value (41; t-test, p = 0.01, 0.0067, 0.0012, 0.0044, respectively; Table 3; Fig 5D; S2C Fig). This was unexpected because CO levels were similar to wild-type for *mlh3-23* mutants and similar to *mlh3Δ* for *mlh3-32* and *mlh3-D523N* mutants (see Discussion). Compared to *mlh3Δ*, the average number of NCO events in *mlh3-23*, *mlh3-32*, and *mlh3-D523N* was similar (p = 0.48, 0.080, 0.70, respectively). Significant increases in NCOs on chromosomes were observed for *mlh3-32* (X and VII), *mlh3-DN* (VIII, X, XIII, VII, XV, IV), *mlh3Δ* (III, VIII, X, XIV, XIII, XVI, XII, VII, XV, IV). No increase in NCOs on specific chromosomes was observed for *mlh3-23* (S2B Fig).

It is possible that some of the increase in NCOs seen in *mlh3* mutants was due to the presence of discontinuous gene tracts that arose from the same initiating DSB. Such discontinuous tracts could have resulted from defective MMR but are not included in the E1 NCO class above. The E4 class (Fig 5B) is a minority discontinuous NCO event that is hypothesized to arise from defective MMR or multiple invasion intermediates [34]. As shown in Fig 5D, none of the E4 events in the *mlh3* mutants were significantly different from wild-type (p = 0.33 to 0.80). This suggests that discontinuous tracts are unlikely to significantly contribute to the increased level of NCO events seen in *mlh3* mutants.

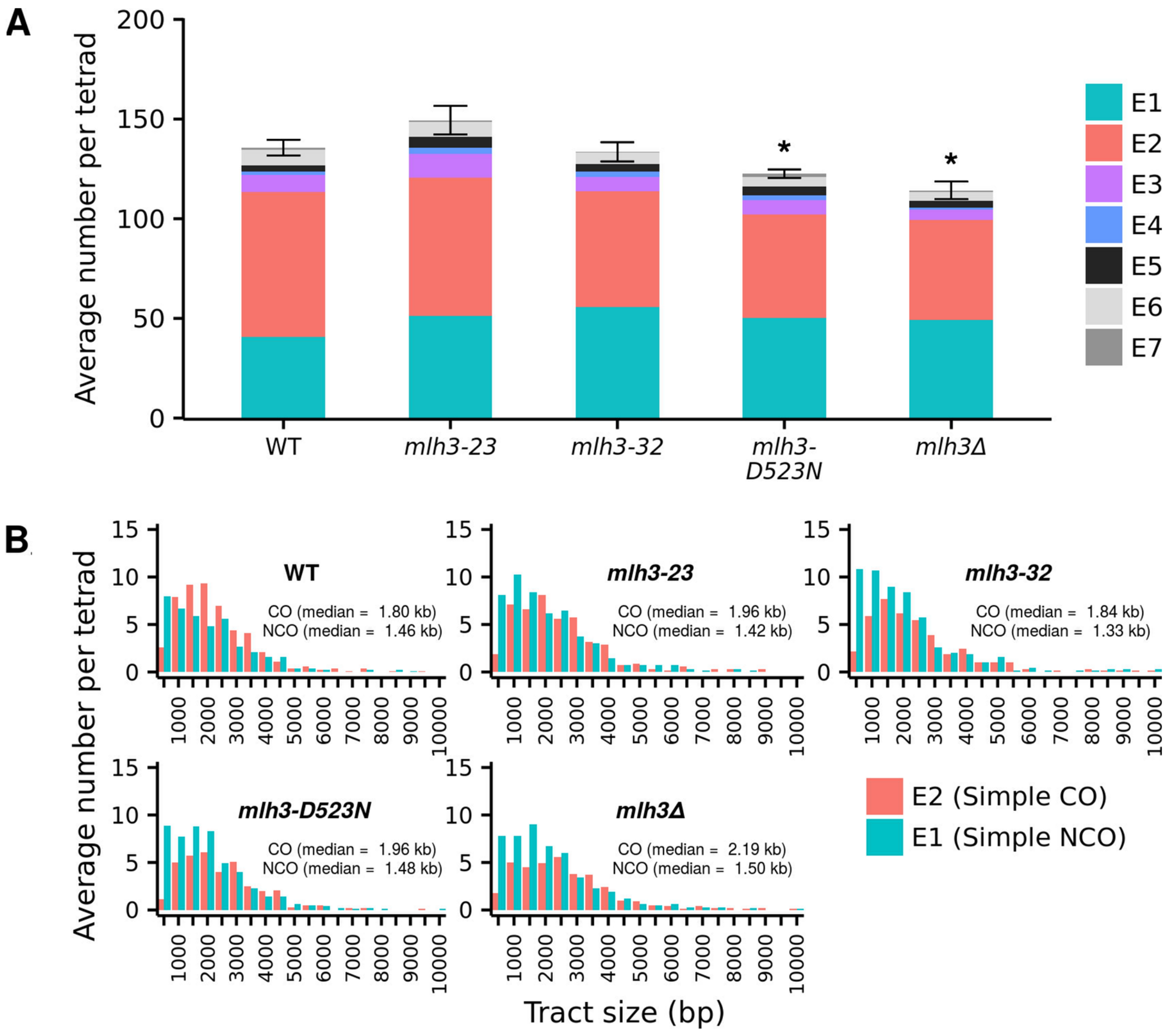

Fig 6. Total interhomolog events and distribution of gene conversion tract lengths associated with NCO and CO events in wild-type, *mlh3-23*, *mlh3-32*, *mlh3-D523N* and *mlh3Δ* mutants. A. Total average inter-homolog events (IH; S9 Table), and each event type (E1-E7) as a fraction of the total, in *mlh3*, *mlh3-23*, *mlh3-32*, *mlh3-D523N*, and *mlh3Δ* mutants. * $p \leq 0.05$ compared to wild-type (S2 File). B. Average number of tracts ordered by size for simple CO (E2) and simple NCO (E1) events. Median CO and NCO tract sizes are also presented, and events were assigned as described by Oke et al. [34] (S2 File). The Y axis represents pooled data from all tetrads, normalized by dividing the number of tetrads.

https://doi.org/10.1371/journal.pgen.1006974.g006

The average NCO (E1) tract lengths were similar to wild-type (1.46 kb) in all of the *mlh3* mutants analyzed (1.34 to 1.50 kb; p = 0.23 to 0.91; S3 Fig). The NCO tract length distribution suggested an enrichment of short NCO tract lengths (<1 kb) in the *mlh3* mutants compared to wild-type (Fig 6B). We found this potentially interesting because Oke et al. [34] identified such an enrichment in *sgs1* mutants; they hypothesized that the short NCO class arose from joint molecules or joint molecule-like intermediates that were resolved by the activity of structure-selective nucleases (SSNs). However, both Kolmogorov–Smirnov and Wilcoxon Rank-Sum tests indicated that an apparent enrichment of short NCO tract lengths in *mlh3* mutants, compared to wild-type, was not significant. A similarly weak pattern (an insignificant increase in *mlh3* mutants compared to wild-type) was seen for the E5A class (S9 Table, [34]), which is thought to represent double COs and is predicted to arise if joint molecule resolution is not biased (Class II COs resolved by SSNs; Fig 1).

One explanation for the increase in NCO events seen in *mlh3Δ*, *mlh3-23*, *mlh3-32* and *mlh3-D523N* mutants is that these mutants experienced meiotic progression delays that resulted in the continued accumulation of NCOs, possibly through increased DSB formation. Increases in NCO events and a meiotic delay were observed in *ndt80* and the ZMM *zip1*, *zip3* and *msh5* mutants as a result of impeding feedback circuits that inhibit DSB formation [27, 30, 49, 71, 72]. To investigate this possibility, we examined total interhomolog (IH) events, which consists of the average of all joint molecule (E2, E3, E5, E6, E7) and NCO (E1, E4) events, and serves as a rough estimate of double-strand break levels [34]. As shown in Fig 6A and S9 Table, the IH value for wild-type was 135.7 events per tetrad. None of the *mlh3* mutants displayed a significantly higher value than wild-type, but *mlh3-D523N* (122.6 events, $p = 0.011$) and *mlh3Δ* (114.2 events, $p = 0.002$) displayed lower values.

We also examined meiotic progression in *mlh3*, *mlh3Δ*, *mlh3-32*, *mlh3-23* and *mlh3-D523N* SK1 strains by measuring the completion of the first meiotic division. This would be difficult to do in S288c/YJM789 strains because they do not show the highly synchronous and efficient meiotic progression profile seen in SK1. As shown in S4 Fig, *mlh3*, *mlh3Δ*, *mlh3-32*, *mlh3-23*, and *mlh3-D523N* mutants showed similar kinetics for completion of at least the first meiotic division (MI+MII). These data suggest that the increase in NCO events in *mlh3Δ*, *mlh3-32*, *mlh3-23*, and *mlh3-D523N* cannot simply be explained by an increase in DSB levels, MMR defects, or a meiotic progression delay.

In summary, the increase in NCO events in *mlh3* mutants is consistent with a possible role for SSNs and/or the STR complex (see Discussion), and is unlikely to be explainable by an increase in DSBs or MMR defects.

Mlh1-mlh3-32 and Mlh1-mlh3-45 display wild-type endonuclease activities but only Mlh1-mlh3-32 endonuclease is stimulated by Msh2-Msh3

We examined Mlh1-mlh3 mutant complexes for endonuclease activity [16, 17], focusing on opposite separation of function mutants Mlh1-mlh3-32 (MMR^+, CO^-), Mlh1-mlh3-6, and Mlh1-mlh3-45 (MMR^-, CO^+). Mlh1-mlh3-45, located in the C-terminal Mlh1 interaction domain, was chosen because it is the only separation of function mutant in that domain that displayed wild-type Mlh1-Mlh3 interactions as measured in the two-hybrid assay (Fig 3C). As shown in Fig 7 and S5 Fig, all three mutant complexes purified as heterodimers and display endonuclease activities similar to wild-type. When this work was initiated we thought that separation of function mutant complexes might show endonuclease defects indicating that this activity is more critical for MMR or crossing over, or show no endonuclease defects because mutant complexes were defective in interacting with MMR or CO specific factors. Our finding that all three mutants have enzymatic activity comparable to wild-type is consistent with the interaction defect model (see below). The *mlh3-6* mutation maps close to conserved sites in the ATP binding motif (S1 Fig). We then tested whether the mutant complex displayed a defect in ATPase activity. As shown in S5C Fig, Mlh1-Mlh3 and Mlh1-mlh3-6 displayed similar ATPase activities.

Because Mlh1-Mlh3's endonuclease activity is enhanced by Msh2-Msh3 [16], we tested whether the opposite separation of function phenotypes of Mlh1-mlh3-32 and Mlh1-mlh3-45 could be explained by defective interactions with MSH complexes. As shown in Fig 7D, Mlh1-mlh3-32 endonuclease activity but not Mlh1-mlh3-45 could be stimulated by Msh2-Msh3. These data are consistent with the MMR^-, CO^+ phenotype exhibited by *mlh3-45* mutants resulting from a defect in interacting with the MMR component Msh2-Msh3, and the *mlh3-32* mutant likely being defective in interactions with meiosis-specific factors. In a first

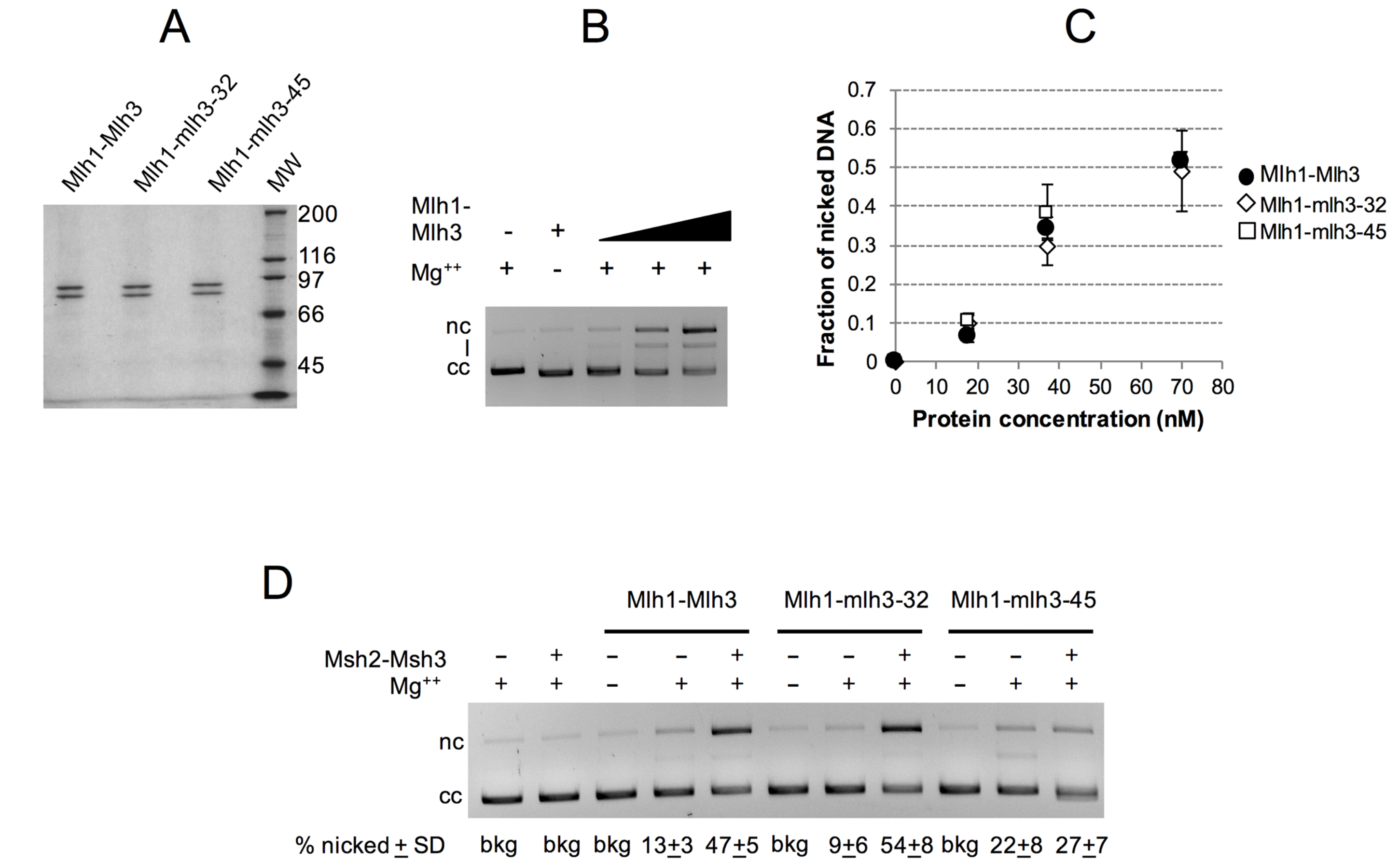

Fig 7. Mlh1-mlh3-32 and Mlh1-mlh3-45 display wild-type endonuclease activities that are differentially stimulated by Msh2-Msh3. A. SDS-PAGE analysis of purified Mlh1-Mlh3, Mlh1-mlh3-32 and Mlh1-mlh3-45. Coomassie Blue R250-stained 8% Tris-glycine gel. 0.5 μg of each protein is shown. MW = Molecular Weight Standards from top to bottom- 200, 116, 97, 66, 45 kD). B, C. Mlh1-Mlh3, Mlh1-mlh3-32 and Mlh1-mlh3-45 (18, 37, 70 nM) were incubated with 2.2 nM supercoiled pBR322 DNA, and analyzed in agarose gel electrophoresis (C) and the endonuclease activity was quantified (average of 6 independent experiments presented +/-SD) as described in the Methods (S2 File). Ladder: 1 kb DNA ladder (New England BioLabs). Migration of closed circular (cc), nicked (nc) and linear (l) pBR322 DNA is indicated. D. Endonuclease assays were performed as in B., but contained 20 nM of the indicated wild-type or mutant Mlh1-Mlh3 complex and 40 nM Msh2-Msh3 when indicated. Reactions were performed in triplicate, samples were resolved on agarose gels, and the fraction of nicked DNA was quantified, averaged, and the standard deviation between experiments was calculated. The average fraction of supercoiled substrate cleaved is presented +/-S.D. below the gel. (bkg) background, (cc) closed circular DNA, (nc) nicked DNA.

https://doi.org/10.1371/journal.pgen.1006974.g007

step to test if *mlh3-32* mutants are defective in interactions with meiotic CO factors, we determined if the *mlh3-32* mutation is dominant; such a phenotype could provide hints on the nature of the *mlh3-32* meiotic defect. We mated EAY3552 (*mlh3-32*, *CEN8Tomato*::*LEU2*) to EAY3339 (relevant genotype *mlh3*, *THR1*::*m-Cerulean-TRP1;* S1 and S2 Tables), and the sporulated progeny displayed a tetratype frequency similar to wild-type (40.9%, n = 252 tetrads), indicating that *mlh3-32* is recessive.

Sgs1 but not sgs1-hd overexpression differentially affects spore viability in *mlh3Δ* vs. *mlh3-32*

As presented in Fig 1, the STR complex can act as both a negative and positive regulator of CO formation in meiotic prophase [8, 30, 31, 46, 49]. In its role as a negative regulator, STR is thought to prevent the formation of aberrant recombination structures by disassembling branched recombination intermediates to form early NCOs via synthesis dependent strand

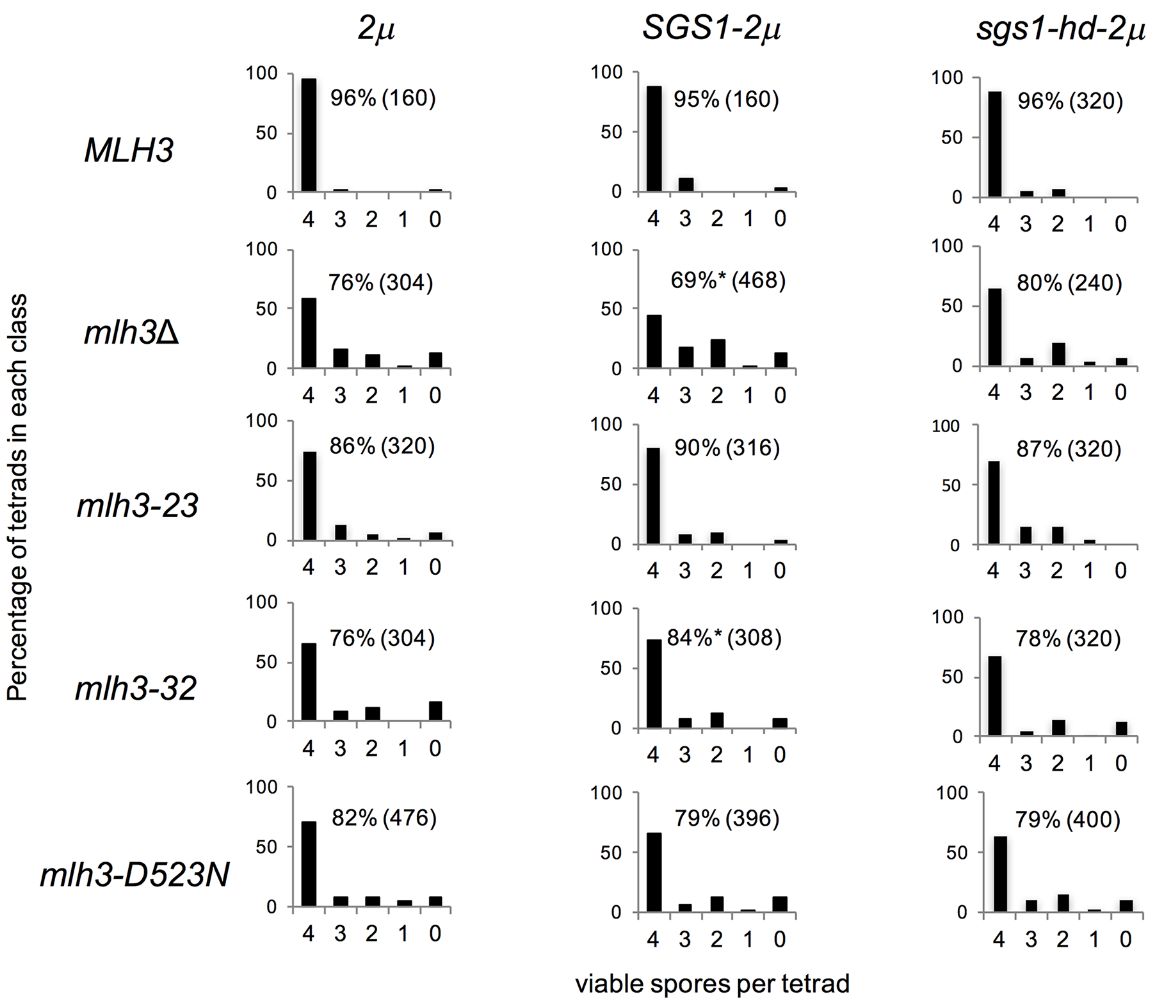

Fig 8. Sgs1 but not sgs1-hd overexpression differentially affects spore viability in *mlh3Δ* vs. *mlh3-32*. Distribution of viable spores in tetrads of *mlh3*, *mlh3Δ*, *mlh3-23*, *mlh3-32*, and *mlh3-D523N* strains containing no insert (*2μ*), p*SGS1-2μ*, or p*sgs1-hd-2μ* (helicase defective mutant). In all plots, the horizontal axis corresponds to the classes of tetrads with 4, 3, 2, 1 and 0 viable spores, and the vertical axis corresponds to the frequency of each class given in percentage. The overall spore viability (SV) and the total number of spores counted (n) are shown (S2 File). *p<0.05 (χ^2 test), comparing spore viability for the indicated strain transformed with a *2μ SGS1* plasmid to that of the same strain transformed with a *2μ* no insert plasmid.

https://doi.org/10.1371/journal.pgen.1006974.g008

annealing (SDSA), or by re-forming the DSB intermediate. In its role as a pro-CO factor STR promotes stabilization of ZMM complexes on recombination intermediates, leading to the resolution of dHJs by an interference-dependent CO pathway (class I) that requires the Mlh1-Mlh3 endonuclease. In *sgs1Δ* mutants COs have been shown to be ZMM independent [73]. Strand invasion intermediates that escape STR disassembly are thought to be resolved as COs or NCOs using an alternative interference-independent CO pathway (class II) that involves the SSNs Mus81-Mms4, Yen1, and Slx1-Slx4.

The increase in NCO events seen in *mlh3* mutants is suggestive of their formation through SSN or STR activities. To test for genetic interactions between *SGS1* and *mlh3*, we expressed *SGS1* via its native promoter on a *2μ* multi-copy vector. Sgs1 overexpression enhanced the *mlh3*Δ spore viability defect (Fig 8: 76% in *mlh3Δ+2μ* vs. 69% in *mlh3Δ+SGS1-2μ*; $p<0.05, \chi^2$ test) and conferred a more apparent MI nondisjunction pattern (an excess of 4, 2, 0 viable

spore tetrads compared with 3 and 1 viable tetrads ([6]; Fig 8). In contrast, Sgs1 overexpression modestly suppressed the *mlh3-32* spore viability defect (Fig 8: 76% in *mlh3-32+2μ* vs. 84% in *mlh3-32+SGS1-2μ*; $p<0.05$, χ^2 test). Suppression or enhancement of phenotypes was not observed upon overexpression of the sgs1-K706A helicase defective mutant protein (sgs1-hd), indicating that these effects were dependent on Sgs1 helicase activity (Fig 8: 80% in *mlh3Δ+sgs1-hd-2μ* and 78% in *mlh3-32+sgs1-hd-2μ*). In addition, no effect was seen upon overexpression of Sgs1 or sgs1-hd in *mlh3* (Fig 8: 96% in *MLH3+2μ* vs. 95% in *MLH3+SGS1-2μ* and 96% in *MLH3+sgs1-hd-2μ*), *mlh3-23* (86% in *mlh3-23+2μ* vs. 90% in *mlh3-23+SGS1-2μ* and 87% in *mlh3-23+sgs1-hd-2μ*) and *mlh3-D523N* (82% in *mlh3-D523N+2μ* vs. 79% in *mlh3-D523N+SGS1-2μ*, and 79% in *mlh3-D523N+sgs1-hd-2μ*) strains. Although *mlh3-32* and *mlh3-D523N* both confer null-like meiotic defects, they did not confer phenotypes similar to *mlh3*Δ in response to Sgs1 overexpression. This observation suggests potential roles for Mlh1-Mlh3 that are independent of its enzymatic activity.

Discussion

We performed a structure-function analysis of Mlh3, a factor that acts in both MMR and meiotic crossing over. This work was pursued because little is known about how Mlh1-Mlh3 acts as a meiotic endonuclease. This is due in part to Mlh1-Mlh3 sharing little in common with the well-characterized structure-selective endonucleases (SSNs; Mus81-Mms4, Slx1-Slx4, and Yen1) in terms of homology and intrinsic behavior *in vitro* (reviewed in [50]). Obtaining new mechanistic insights has been complicated by the fact that Mlh1-Mlh3 can bind to model HJ substrates, but cannot cleave them, and by genetic studies suggesting that Mlh1-Mlh3 acts in concert with other pro-CO factors [16, 17, 50, 74]. Recent work has suggested that multiple Mlh1-Mlh3 heterodimers are required to activate the endonuclease and that the complex is at least partially inhibited by incorporation of a DNA secondary structure that is not part of a continuous homoduplex substrate [74]. These data suggest that other protein factors likely recruit and position Mlh1-Mlh3 complexes during meiotic recombination. The identities of these factors are for the most part known, though it is not understood how they directly contribute to Mlh1-Mlh3's ability to nick DNA in the directed manner required to generate COs.

Our analysis of *mlh3-32* (MMR^+, CO^-) and *mlh3-45* (MMR^-, CO^+), support the above hypothesis that protein-protein interactions are critical for directing Mlh1-Mlh3 endonuclease activity (Table 2; Fig 7). Mlh1-Mlh3 has been shown genetically to act downstream of Msh4-Msh5 [40, 41, 44, 47]; this order of events is analogous to steps in DNA MMR where MLH acts following MSH recognition [14, 75]. As outlined in the introduction, Msh4-Msh5, STR, Exo1 (independent of its enzymatic activity) and Zip3 have been classified as pro-CO factors, and have all been shown to interact with one another and/or with Mlh1-Mlh3 (reviewed in [50]). Our biochemical studies are consistent with Mlh1-mlh3-45 having interaction defects that prevent its endonuclease activity from being stimulated by Msh2-Msh3 in MMR. We hypothesize that MLH complexes interact with MSH complexes via a common mechanism, and that defective interactions with Msh2-Msh3 are also indicative of defective interactions with Msh4-Msh5, but during meiotic CO resolution, additional factors act in concert to strengthen a possibly weakened Msh4-Msh5-Mlh1-mlh3-45 interaction. This model also helps explain why we identified several MMR^- CO^+ *mlh3* mutants (*mlh3-42* and *-54*) in which the mutant mlh3 protein fails to interact with Mlh1. For the Mlh1-mlh3-32 complex, the MSH interaction and enhancement is retained, but interaction with other critical meiotic factors is likely lost, possibly resulting in an unstable complex that cannot resolve dHJs.

Mlh1-Mlh3 in DNA mismatch repair

In MMR the asymmetric loading of PCNA by the RFC complex is thought to direct the endonuclease activity of MutLα (Mlh1-Pms1 in *S. cerevisiae*, MLH1-PMS2 in humans) to act in strand-specific repair [76]. Additional studies suggest that specific protein-protein interactions influence and activate MLH endonuclease activity, and direct nicking to a specific location. For example, *in vitro* studies performed with yeast proteins showed that RFC-loaded PCNA can activate Mlh1-Pms1 but not Mlh1-Mlh3 endonuclease on circular plasmids (Mlh3 lacks a PCNA binding motif present in Pms1; [15–17; 34]. Our finding that endonuclease active site residues are highly conserved between Mlh1-Mlh3 and Mlh1-Pms1, which has no role in meiotic crossing over, suggests that the different functions of the two complexes are a result of the different protein-protein interactions.

Data from the Crouse lab suggest that Mlh1-Mlh3 acts in conjunction with Mlh1-Pms1 in Msh2-Msh3 dependent MMR [4]. This observation helps address how Mlh1-Mlh3 is involved in MMR in the absence of a PCNA interaction. In one scenario consistent with the above, Mlh1-Mlh3 is recruited and activated by Msh2-Msh3, and also forms a complex with Mlh1-Pms1, which can be directed by PCNA to promote efficient repair. If dimerization between Mlh3 and Mlh1 is weakened, the ability to be recruited by Msh2-Msh3 and interact with Mlh1-Pms1 is likely inhibited, creating a defect in MMR that is minor because Mlh1-Pms1 is still active. For meiotic crossing over, a relatively slow process compared to DNA MMR at the replication fork, we suggest that a weakened dimer can be compensated for by interactions with other meiotic factors (e.g. Msh4-Msh5, Exo1 and STR). Thus, our work provides further motivation to examine Mlh1-Mlh3 activity on recombination substrates in the presence of pro-CO factors.

Mlh1-Mlh3 in meiotic recombination

Current meiotic DSB repair models postulate an enzymatic role for Mlh1-Mlh3 in the class I CO pathway after DSB intermediates have been captured and stabilized by the ZMM proteins [8, 30, 49]. In these models DSB intermediates that escape capture by ZMM proteins are resolved into class II COs or NCOs by SSNs, though NCOs mostly arise from the action of the STR complex through synthesis dependent strand annealing (SDSA; [30, 49]; Fig 1).

We observed a genome-wide increase in NCOs compared to wild-type in *mlh3-23*, *mlh3-32*, *mlh3-D523N*, and *mlh3Δ* mutants. This suggested to us that in mutants defective in Mlh1-Mlh3 functions, dHJs formed in the ZMM pathway become susceptible to the actions of SSNs to form COs and NCOs. They could also be acted upon by STR through unwinding and convergent migration of the two HJs until a single pair of crossing strands in a hemicatenane can be removed by topoisomerase activity, giving rise to NCOs [30,31]. In this model Mlh1-Mlh3, in concert with the ZMM proteins, acts to protect recombination intermediates from STR. In support of this idea, we identified genetic interactions between Mlh1-Mlh3 and Sgs1 that suggest an active interplay (Mlh1 and Mlh3 both interact with Sgs1 [42,77]) between these factors: Sgs1 overexpression decreased the spore viability of *mlh3Δ* strains but not *mlh3*, *mlh3-23*, or *mlh3-D523N* strains, where a structurally intact Mlh1-Mlh3 complex is thought to be present, and modestly increased the spore viability of *mlh3-32* mutants (Fig 8).

We also observed an increase in tract length for gene conversion events associated with COs in *mlh3Δ* compared to wild-type (S3 Fig; S8 and S9 Tables). The latter observation is interesting because recent work from Duroc et al. [78] provided evidence that another MLH complex, Mlh1-Mlh2, which lacks endonuclease activity, acts to limit the extent of meiotic gene conversion. They found that gene conversion tract lengths associated or not associated with COs increased from ~1 kb in wild-type to ~2 kb in *mlh2Δ*. Thus, regulating gene

conversion tracts may be a common property of the MLH complexes that act in meiotic recombination, and is consistent with the fact that Mlh1 has been shown to physically interact with helicases that can modulate heteroduplex extension/migration.

An alternative explanation for the increase in NCO events in *mlh3* mutants is that delays in meiotic progression result in the accumulation of NCO events as the result of increased DSB formation [72]. However, we did not observe such delays in any of the *mlh3* mutant backgrounds or any dramatic change in DSB formation based on an analysis of total interhomolog events in *mlh3* mutants, though a direct measure of DSB levels is critical to resolve this issue (Fig 6A; S4 Fig; S9 Table; [69]).

Mlh3's linker arm is critical for its meiotic function

MLH proteins act as dimers and contain long unstructured linkers that connect the N- and C-terminal domains of each subunit. These linkers vary in length and are resistant to amino acid substitutions [54]. Previous work showed that the Mlh1-Pms1 heterodimer undergoes large global conformational changes in an ATP binding and hydrolysis cycle [79]. In this cycle the linkers act as arms that can switch between extended and condensed states. These conformational changes are hypothesized to be important to expose different domains of the heterodimer for new protein-protein or protein-DNA interactions in addition to mediating the timing of these interactions [79], and have also been implicated in *B. subtilis* MutL for "licensing" its latent endonuclease activity [60]. In addition, a series of truncation mutants in Mlh1-Pms1 indicate that the Pms1 linker arm appears more important than the Mlh1 linker arm for DNA binding [80]. Extending these ideas to Mlh1-Mlh3, it is interesting to note that the MMR^{+},CO^{-} *mlh3-32* allele maps to the unstructured linker, suggesting that this domain is particularly important in crossing over (S1 Fig; Table 1), possibly facilitating interactions with CO promoting factors that in turn direct and position Mlh3's endonuclease activity on recombination substrates. It is important to note that Claeys Bouuaert and Keeney [81] identified mutations in the Mlh3 linker domain based on a biochemical analysis of Mlh1-Mlh3 that overlap with residues mutated in the *mlh3-32* allele. Interestingly, the mutations that they identified also conferred a greater defect in crossing over than in MMR, consistent with our analysis of *mlh3-32*. In addition, they found that mutations within and near the *mlh3-32* allele compromised DNA binding activity of Mlh1-Mlh3, suggesting that DNA binding within the linker region may be important for meiotic functions, though we did not detect any apparent defect in the endonuclease activity of Mlh1-mlh3-32.

Alanine-scan mutagenesis of Mlh1 [54] and Mlh3 have provided us with additional information regarding the unstructured linkers in Mlh proteins. Previously we used protein structure prediction and molecular analyses to map the Mlh1 unstructured linker to amino acids 336 to 480 [80]; a similar analysis mapped the Mlh3 unstructured linker to amino acids 373 to 490 [16]. As in the analysis of the Mlh3 random coil, few mutations were identified in the Mlh1 unstructured linker that conferred defects in MMR and crossing over. For example, in Mlh1, no mutations were identified between amino acids 427 and 490 that conferred mutator phenotypes. However, similar to results seen for Mlh3 (S1 Fig; Table 1), mutations were identified just before the unstructured linker in Mlh1 (253–312) that conferred strong mutator phenotypes [54]. Curiously, the corresponding region in MutL contains residues that have been linked through crystallographic analysis to DNA binding [82], suggesting that the organization of the DNA binding and unstructured linker domains in the MLH proteins is conserved. Finally, in both Mlh1 and Mlh3, a localized set of mutations within the center of the unstructured linker (390–403 in Mlh1, 414–416 in Mlh3) affect function, suggesting that this specific region is likely to play an important function beyond serving as a random coil.

Closing thoughts

Mlh1-Mlh3 appears to be acting in CO resolution through a novel mechanism distinct from known structure-selective endonucleases (75). Mlh1-Mlh3 does not share conservation with the known endonuclease superfamilies (XPF, URI-YIG, Rad2/XPG), and does not appear capable of resolving model HJ substrates [50, 74]. As mentioned previously, dHJ resolution by Mlh1-Mlh3 results in only CO products whereas the interference-independent CO pathway, which is dependent on Mus81-Mms4, resolves dHJs into a mixture of CO and NCO products [8]. Thus, Mlh1-Mlh3's distinct activity suggests that its nicking is positioned by pro-CO factors such as Msh4-Msh5, Zip3, the STR complex, and Exo1. Such factors are likely to orient Mlh1-Mlh3 to promote asymmetric cleavage of dHJs in a highly regulated and coordinated manner. Thus our work provides further motivation to examine Mlh1-Mlh3 activity on recombination substrates in the presence of pro-CO factors.

Polymorphisms in human *mlh3* genes have been associated with male and female infertility [83–85], and errors in meiotic chromosome segregation are considered a leading cause of spontaneous miscarriages and birth defects [13]. It is interesting to note that the *mlh3-23* mutation, which only weakly affected crossing over, conferred an alteration in meiotic recombination outcomes that was similar to that seen in *mlh3* mutants that conferred more severe defects (Fig 5). This observation suggests that some polymorphisms in meiotic recombination genes could have more severe defects in human fertility than expected.

Methods

Media

S. cerevisiae SK1, S288c, and YJM789 strains were grown on either yeast extract-peptone-dextrose (YPD) or minimal complete media at 30˚C [86]. For selection purposes, minimal dropout media lacking uracil was used when needed. Geneticin (Invitrogen, San Diego), Nourseothricin (Werner BioAgents, Germany) and Hygromycin (HiMedia) were added to media when required at recommended concentrations [87, 88]. Cells were sporulated as described by Argueso et al. [7].

Site-directed mutagenesis of *mlh3*

60 *mlh3* alleles were constructed, resulting in the mutagenesis of 139 amino acids in the 715 amino acid Mlh3 polypeptide (S1 Table). The single-step integration vector (pEAI254), containing the SK1 *mlh3* gene with a *KANMX4* selectable marker inserted 40 bp downstream of the stop codon [5], was used as a template to create plasmids (S3 Table) bearing the *mlh3* mutant alleles via QuickChange site directed mutagenesis (Stratagene, La Jolla, CA). *mlh3-60*, in which the last 11 residues of Pms1 (DWSSFSKDYEI) were inserted before the *mlh3* stop codon, was also made by QuickChange. Mutations were confirmed by sequencing the entire open reading frame (Sanger method), as well as 70 bp upstream and 150 bp downstream. Primer sequences used to make and sequence these variants are available upon request.

Mlh3 homology model

The amino acid sequence of *S. cerevisiae* Mlh3 (YJM789) was used to construct a homology model from HHpred (http://toolkit.tuebingen.mpg.de/hhpred) and Modeller software. PyMOL was used for imaging.

Construction of strains to measure meiotic crossing over and MMR

The SK1 strain EAY3255 (S1 Table) was constructed to allow for the simultaneous analysis of *mlh3* MMR and meiotic crossing over phenotypes. It carries a spore autonomous fluorescent protein marker (RFP) on chromosome VIII to monitor chromosome behavior (crossing over and nondisjunction; [56]) as well as the *lys2::InsE-A*$_{14}$ cassette to measure reversion to Lys$^+$ [55]. pEAI254 and mutant derivatives described above and in S3 Table were digested with *Bam*HI and *Sal*I and introduced into EAY3255 by gene replacement using the lithium acetate transformation method as described in Gietz et al. [89]. At least two independent transformants for each genotype (verified by sequencing) were made resulting in a total of 120 haploid strains bearing the *mlh3* variants described in this study (S1 Table). These haploid strains were used to measure the effect of *mlh3* mutations on reversion rate and were mated to EAY3486, an *mlh3Δ* strain containing the CFP marker, resulting in diploid strains suitable for analysis of crossing over (S2 Table). Diploids were selected on media lacking the appropriate nutrients and maintained as stable strains. Meiosis was induced upon growing the diploid strains on sporulation media as described in Argueso et al. [7]. Wild-type strains carrying the fluorescent protein markers used to make the above test strains were a gift from the Keeney lab.

Lys$^+$ reversion assays

The haploid strains described above were analyzed for reversion to Lys$^+$ as described in Tran et al. [55]. At least 10 independent cultures were analyzed for each mutant allele alongside wild-type or *mlh3Δ* controls. Analyses were performed for two independent transformants per allele. Reversion rates were measured as described [90, 91], and each median rate was normalized to the wild-type median rate (1X) to calculate fold increase. Alleles were classified into a wild-type, intermediate, or null phenotype based on the 95% confidence intervals which were determined as described [92].

Spore autonomous fluorescent protein expression to measure percent tetratype

Diploids in the EAY3255/EAY3486 background described above (S2 Table) were sporulated on media described in Argueso et al. [7]. Spores were treated with 0.5% NP40 and briefly sonicated before analysis using the Zeiss AxioImager.M2 [56]. At least 250 tetrads for each *mlh3* allele were counted to determine the % tetratype. Two independent transformants were measured per allele. A statistically significant difference ($p<0.01$) from wild-type and *mlh3Δ* controls based on χ^2 analysis was used to classify each allele as exhibiting a wild-type, intermediate, or null phenotype.

Meiotic time courses

Meiotic time course were performed as described in Sonntag Brown et al. [93] for the diploid strains EAY3252/EAY3486 (*mlh3*), EAY3255/EAY3486 (*mlh3Δ*), EAY3534-35/EAY3486 (*mlh3-23*), EAY3552-53/EAY3486 (*mlh3-32*), and EAY3819-20/EAY3486 (*mlh3-D523N*; S2 Table). Strains in single time courses were grown in the same batch of media under identical conditions. Aliquots of cells at specific time points were stained with DAPI to determine the percentage of cell that completed the first meiotic division (cells in which 2, 3, or 4 nuclei were observed by DAPI staining, presented as MI+MII). Cells were visualized using a Zeiss Axio Imager M2 microscope equipped with a DAPI filter. At least 150 cells were counted for each time point. Two independent transformants were analyzed per allele.

Yeast two-hybrid analysis

The L40 strain [94] was co-transformed with bait and target vectors. Residues 481–715 of the Mlh3 C-terminus were PCR amplified from pEAI254 (SK1 *mlh3* described above) and mutant derivatives, and then sub-cloned into the target vector pEAM98 (S288C *mlh3*) to make pEAM234 (SK1 *mlh3*) and mutant derivatives (S3 Table). pEAM98 contains a fusion between the GAL4 activation domain in pGAD10 and residues 481–715 of the Mlh3 C-terminus [5, 36]. The resulting SK1 derived target vectors were confirmed by sequencing (Sanger method). The bait vector used was pEAM105 (pBTM-MLH1-SK1). Expression of the *LACZ* reporter gene was determined by the ortho-nitrophenyl-β-D-galactopyranoside (ONPG) assay as described in [95].

Purification of Mlh1-Mlh3 and mutant complexes from baculovirus-infected Sf9 cells

Mlh1-Mlh3 and Mlh1-mlh3 mutant derivatives were purified from Sf9 cells infected with Bac-to-Bac baculovirus expression system using pFastBacDual constructs [16]. Mutant Mlh1-mlh3 complexes were purified using the same protocol developed to purify wild-type Mlh1-Mlh3. This involved the use of successive nickel-nitroloacetic acid-agarose (Qiagen) and heparin sepharose (GE Healthcare) column purifications. Mlh1-Mlh3 and mutant derivative yields were ~150 μg per 5 x 10^8 cells; aliquots from the final heparin purification were frozen in liquid N_2 and stored at -80˚C. Protein concentrations were determined by Bradford assay [96] using BSA standard. The *mlh3-6*, *mlh3-32* and *mlh3-45* mutations were introduced into pEAE358 (pPH-His_{10}-MLH3-HA pFastBacDual construct; Rogacheva et al. [16]) by Quick Change (Stratagene). *His_{10}-mlh3-HA* fragments were individually subcloned by restriction digestion into pEAE348 to form pFastBacDual constructs pEAE382 (Mlh1-mlh3-6), pEAE383 (Mlh1-mlh3-32) and pEAE384 (Mlh1-mlh3-45), in which the *MLH1-FLAG* gene is downstream of the p10 promoter and the *His_{10} -mlh3-HA* gene is downstream of the pPH promoter. The sequence of the restriction fragments inserted into pEAE348 were confirmed by DNA sequencing (Cornell Biotechnology Resource Center). Msh2-Msh3 was purified as described previously [97].

Endonuclease assay on supercoiled plasmid DNA and ATPase assay

Mlh1-Mlh3 nicking activity was assayed on supercoiled pBR322 or pUC18 (Thermo Scientific). DNA (2.2 nM) was incubated in 20 μl reactions containing indicated amounts of Mlh1-Mlh3 and Msh2-Msh3 [97] in 20 mM HEPES-KOH pH 7.5, 20 mM KCl, 0.2 mg/ml BSA, 1% glycerol, and 1 mM $MgCl_2$ for 1 h at 37˚C. Reactions were quenched by incubation for 20 min at 37˚C with 0.1% SDS, 14 mM EDTA, and 0.1 mg/ml proteinase K (New England Biolabs) (final concentrations). Samples were resolved by 1% agarose gel with 0.1 μg/ml ethidium bromide run in 1X TAE buffer for 50 min at 95 V. All quantifications were performed using GelEval (FrogDance Software, v1.37). The amount of nicked product was quantified as a fraction of the total starting substrate in independent experiments. *bkg* indicates that amount of nicked product was not above background levels established by negative controls. ATPase assays were performed as described [16].

Genetic map distance analysis

Diploids from the SK1 congenic strain background EAY1112/1108 [5–7] were used for genetic map distance analyses. EAY1112/2413 (*MLH3/mlh3Δ::NATMX*) and EAY1848/2413 (*mlh3Δ::KANMX/mlh3Δ::NATMX*) were used as wild-type and null controls respectively (S2 Table).

mlh3 alleles of interest were integrated into EAY3712 (same as EAY1112 but *mlh3Δ*::*URA3*) using standard techniques [89]. The resulting haploid strains (EAY3713-EAY3724) were mated to EAY2413 (*mlh3Δ*::*NATMX*) giving rise to diploids carrying markers suitable for genetic distance measurements (S2 Table). Two independent transformants were analyzed per allele. Diploids were selected on media lacking the appropriate nutrients and maintained as stable strains. Diploids were sporulated as described [7]. Tetrads were dissected on synthetic complete media and germinated at 30˚C after an incubation of 2–3 days. Spore clones were then replica-plated on various selective media to be scored after 1 day of incubation at 30˚C. Chromosome behavior was analyzed using the recombination analysis software RANA to measure genetic map distances and spore viabilities [7]. Genetic map distances ± SE were calculated using the formula of Perkins [98] through the Stahl Laboratory Online Tools portal (http://molbio.uoregon.edu/~fstahl/).

Construction of strains for whole genome sequencing

Whole genome sequencing was performed using S288c/YJM789 hybrid diploids. The Mlh1 protein sequence has four amino acid differences between SK1 and YJM789 strains and five amino acid differences between SK1 and S288c strains. The SK1 Mlh3 protein has 11 amino acid differences with respect to S288c Mlh3 and seven with respect to YJM789 Mlh3. To avoid genetic incompatibilities between Mlh1 and Mlh3, we analyzed the SK1 *mlh3* mutations, including the wild-type and *mlh3Δ* controls in the presence of SK1 *MLH1* in the S288c/YJM789 hybrid. Strain genotypes are shown in S1 and S2 Tables. SK1-*MLH1 and MLH3* alleles were introduced into wild-type YJM789 and S288c *mlh3Δ*::*natMX4*, respectively, using plasmids pEAA214 and pEAI254. The SK1 *MLH1* specific SNPs were confirmed by Sanger sequencing. The *mlh3-23*::*kanMX4*, *mlh3-32*::*kanMX4*, *and mlh3-D523N*::*kanMX4* mutations were introduced using plasmids pEAI347, pEAI356, and pEAI252, respectively into the S288c *mlh3Δ*::*natMX4* background. The S288c *mlh1Δ*::*hphMX4* and YJM789 *mlh3Δ*::*kanMX4* strains were made using an *mlh1Δ*::*hphMX4* construct amplified by PCR and the *mlh3Δ*::*kanMX4* deletion construct pEAI168, respectively.

Genome wide mapping of meiotic recombination events in the S288c/YJM789 hybrid

Genomic DNA was extracted from spore colonies of four viable spore tetrads of the *mlh3* point mutants, isogenic wild type, and isogenic *mlh3Δ* mutants as described previously [64]. Whole genome sequencing on the Illumina Hi-Seq 2500 platform was performed at Fasteris, Switzerland. Raw sequence reads were processed and SNPs genotyped as described in Krishnaprasad et al. [64] and Chakraborty et al. [70], with 4:0 marker segregations shared between tetrads removed from the analysis. Analysis of genome wide recombination events was performed as described in Oke et al. [34] (S5 to S9 Tables). Custom R scripts were used to generate the segregation file (input file for the CrossOver program (v6.3) of the ReCombine [99] program suite (v2.1)). The output of the CrossOver program was used as input for groupEvents (v15). Median crossover associated tract lengths were calculated from E2 events with non-zero tracts [34]. For all count data, the t-test was used to examine the difference in the mean between samples. For tract lengths, the Wilcoxon Rank-Sum test was used to test the difference in the median between samples.

The segregation files, CrossOver program output files, groupEvents output files and the custom R scripts are available online at the Dryad digital repository (http://dx.doi.org/10.5061/dryad.bb702). Sequence data are available from the National Centre for Biotechnology Information Sequence Read Archive (Accession numbers SRP096621 and SRP110341).

Overexpression of Sgs1 and sgs1-hd

SGS1 (native promoter, ORF, and termination sequence) was PCR amplified from SK1 genomic DNA obtained from NKY730 (*MATa/alpha*, *ura3Δ*::*hisG/ura3Δ*::*hisG*, *leu2*::*hisG/leu2*::*hisG*, *lys2/lys2*) and cloned into the high copy vector pRS426 (*2μ*, amp^R, *URA3*) to make pEAM266 (S3 Table). The correct DNA sequence was confirmed by Sanger sequencing the entire insert. *sgs1-K706A* the helicase defective variant of *SGS1* (*sgs1-hd*) was made by site-directed mutagenesis of pEAM266 (New England BioLabs Q5 kit). The correct *sgs1-hd*, *2μ*, and *URA3* sequences were confirmed by Sanger sequencing. Primer sequences used to make these constructs are available upon request. The high copy vector (with or without the *SGS1* or *sgs1-hd* insert) was transformed into stable diploids of EAY3534-35/EAY3468 (*mlh3-23/mlh3*Δ), EAY3552-53/EAY3486 (*mlh3-32*/*mlh3*Δ), EAY3819-20/EAY3486 (*mlh3-D523N/mlh3*Δ), EAY3252/EAY3486 (*MLH3/mlh3*Δ), and EAY3255/EAY3486 (*mlh3Δ/mlh3Δ*) (S2 Table). Meiosis was induced as described in Argueso et al. [7] and vector selection was maintained by growing the diploid strains on minimal media lacking uracil prior to sporulation. In addition, sporulation media lacked uracil. For spore viability measurements, tetrads were dissected on synthetic complete media and germinated at 30˚C after an incubation of 2–3 days. At least two independent transformants were analyzed per high copy vector. Differences in spore viability were assessed for significance using the χ^2 test.

Supporting information

S1 File. Segregation of SNPs in all 44 tetrads as labeled in S6 Table. S288c and YJM789 SNPs are shown in red and blue, respectively.
(PDF)

S2 File. Data underlying tables and figures. This file is cited in the corresponding table and figure legends.
(XLSX)

S1 Fig. Structure-function map of *Saccharomyces cerevisiae* Mlh3. Mismatch repair (top) and crossing over (bottom) phenotype of *mlh3* (blue) vs *mlh3Δ* (red) (Fig 3B). Mismatch repair was measured using the *lys2-A*$_{14}$ reversion assay [55] and crossing over was measured using the assay [56] depicted in Fig 3A. Bars represent the median reversion rates and percent tetratype normalized to *mlh3* (1X). The vertical bars indicate the approximate position of the *mlh3* mutations analyzed in this study with the height of each bar corresponding to the phenotype relative to *mlh3* (1X). Red indicates a null phenotype, blue indicates wild-type (WT), and green indicates intermediate. For mismatch repair (top), bars represent reversion rates of at least 10 independently tested cultures from two independently constructed strains presented here normalized to *mlh3* median rate 1X = 1.43×10^{-6} (n = 140). For crossing over (bottom), bars represent percent tetratype of at least 250 tetrads from two independently constructed strains presented here normalized to *mlh3* percent tetratype 1X = 36.7% (n = 226). Blue and red dotted lines represent *mlh3* and *mlh3Δ* respectively. Black star indicates separation of function mutants (Table 1 and S2 File).
(TIFF)

S2 Fig. Crossover and noncrossover distribution on chromosomes for wild-type, *mlh3-23*, *mlh3-32*, *mlh3-D523N* and *mlh3Δ*. A. and C. Scatter plot of average crossover (CO, E2+E3) and noncrossover (NCO, E1) counts per chromosome against chromosome size [34]. The equations for the regression lines are:

wild-type (CO = 5.85×10^{-6} x chr. size + 0.65; NCO = 2.99×10^{-6} x chr. size + 0.28);

mlh3-23 (CO = 5.6 x 10^{-6} x chr. size + 0.84; NCO = 4.46 x 10^{-6} x chr. size − 0.15);

mlh3-32 (CO = 4.79 x 10^{-6} x chr. size + 0.45; NCO = 4.51 x 10^{-6} x chr. size + 0.08);

mlh3-D523N (CO = 3.8 x 10^{-6} x chr. size + 0.77; NCO = 3.75 x 10^{-6} x chr. size + 0.31);

mlh3Δ (CO = 3.65 x 10^{-6} x chr. size + 0.69; NCO = 3.94 x 10^{-6} x chr. size + 0.1).

B. and D. Bar plot of average crossover and noncrossover counts per chromosome. The asterisk symbol (*) marks chromosomes that have significant difference (two tailed t-test for difference in mean; $P<0.05$) in crossover / noncrossover counts compared to wild-type. Chromosomes are arranged by size from left to right. Error bars are mean ± standard error (S2 File).
(TIFF)

S3 Fig. Distribution of gene conversion tract lengths associated with crossovers and noncrossovers (NCOs) for wild-type, *mlh3-23*, *mlh3-32*, *mlh3-D523N*, and *mlh3Δ*. A. Distribution of gene conversion tract lengths associated with simple NCO (E1) and CO (E2) events (S2 File). B. Distribution of gene conversion tract lengths associated with simple NCO (E1) and discontinuous NCO (E4) events. * $p \leq 0.05$ comparing *mlh3Δ to mlh3-32*. In A and B the minimum, first quantile, median, third quantile, and maximum count are indicated in the box plot. Outlier points are not shown (S2 File).
(TIFF)

S4 Fig. *mlh3* mutants display normal meiotic prophase progression as measured by the completion of the first meiotic division. Representative time courses showing the completion of the MI division (MI+MII) in wild-type, *mlh3-23*, *mlh3-32*, *mlh3-D523N* and *mlh3Δ* strains. Cells with two, three, or four nuclei were counted as having completed MI (MI+MII). All strains for a single time course were grown in the same batch of media under identical conditions. Two independent transformants were measured per allele (S2 File).
(TIFF)

S5 Fig. Mlh1-mlh3-6 exhibits wild-type endonuclease and ATPase activity. A. SDS-PAGE analysis of purified Mlh1-Mlh3 and Mlh1-mlh3-6. Coomassie Blue R250-stained 8% Tris-glycine gel. 0.5 μg of each protein is shown. MW = Molecular Weight Standards from top to bottom-116, 97, 66, 45, 31 kD). B, C. Mlh1-Mlh3 and Mlh1-mlh3-6 (18, 37, 70 nM) were incubated with 2.2 nM supercoiled pBR322 DNA, analyzed in agarose gel electrophoresis, and endonuclease activity was quantified (average of 6 independent experiments presented +/-SD) as described in the Methods (S2 File). C. ATPase assays were performed as described in Rogacheva et al. [16], but contained the indicated amounts of Mlh1-Mlh3 and Mlh1-mlh3-6 incubated with 100 μM ^{32}P-γ-ATP. Reactions were performed in duplicate for two separate purifications of each, and the average values, +/-SD, are presented (S2 File).
(TIFF)

S1 Table. Yeast strains used in this study. EAY3252, EAY3255 and derivatives, and EAY3486 are SK1 strains that contain spore autonomous fluorescent markers described in Thacker et al. [56]. EAY1112 and EAY2413 contain chromosome XV markers described in Argueso et al. [7]. KTY strains were used for whole genome recombination mapping as described in Krishnaprasad et al. [64] and Oke et al. [34].
(PDF)

S2 Table. Diploid strains used to measure % tetratype, spore viability, meiotic progression, genetic map distances and for whole genome recombination mapping. The indicated

haploid strains were mated to form the diploids with the relevant genotype shown.
(PDF)

S3 Table. Plasmids used in this study. A. All *mlh3* mutagenesis plasmids are derived from pEAI254, a 7.8 kb $mlh3_{SK1}$::*KANMX* integrating vector. pEAI254 was mutagenized by Quick-Change to create the alleles listed. The DNA sequence of the entire ORF, and 70 bp upstream and 150 bp downstream, were confirmed by DNA sequencing using primers EAO318, EAO319, EAO1778 and EAO321. B. For the two-hybrid analysis, pEAM105 contains the entire *MLH1* gene derived from the SK1 strain inserted immediately after the lexA binding domain in pBTM116. All *GAL4* activating domain-*mlh3* plasmids are derived from pEAM234, which contains DNA sequence encoding SK1 MLH3 amino acids 481 to 715 inserted immediately after the GAL4 activating domain in pGAD424. C. *SGS1* was cloned into pRS426 to make pEAM266 as described in the Methods. Site directed mutagenesis of pEAM266 was performed to make pEAM270 carrying the helicase defective *sgs1* allele as described in the Methods.
(PDF)

S4 Table. Genetic map distances for *mlh3* separation of function mutants on chromosome XV from single spores and tetrads. All *mlh3* mutants are isogenic derivatives of EAY1112/EAY2413 (S2 Table; Methods). For single spores, recombination frequencies (recombinant spores/total spores) were multiplied by 100 to yield genetic map distances (cM). For tetrads, genetic distance in centimorgans (cM) was calculated using the RANA software without considering aberrant segregants (Argueso et al. [7]). The Stahl Laboratory Online Tools website (http://molbio.uoregon.edu/~fstahl/) was used to calculate standard error (SE) around the genetic distance for tetrads. n; number of single spores, N; four spore viable tetrads analyzed; Par, parental single spores; Rec, recombinant single spores.
(PDF)

S5 Table. Sequencing statistics for spores derived from wild type, *mlh3-23*, *mlh3-32*, *mlh3-D523N* and *mlh3Δ* in S288c/YJM789 hybrid bearing SK1-*MLH1/MLH3* alleles.
(PDF)

S6 Table. Crossovers (CO) and noncrossovers (NCO) in tetrads of wild-type, *mlh3-23*, *mlh3-32*, *mlh3-D523N* and *mlh3Δ*. Crossovers are E2+E3 events and noncrossovers are E1 events [34] (S2 File).
(PDF)

S7 Table. Average crossovers (CO) and noncrossovers (NCO) per chromosome for WT (wild type), *mlh3-23*, *mlh3-32*, *mlh3-D523N* and *mlh3Δ* mutants. Crossovers are E2+E3 events and noncrossovers are E1 events [34] (S2 File).
(PDF)

S8 Table. Gene conversion tract lengths in *mlh3Δ*, wild type, *mlh3-23*, *mlh3-32* and *mlh3-D523N* mutants. E1, E2, E4 are classified by groupEvents [34]. P-values show statistical significance of difference in median CO and NCO gene conversion tract lengths of wild-type and *mlh3* alleles compared to *mlh3Δ* using the Wilcoxon rank sum test (Methods; S2 File).
(PDF)

S9 Table. Summary of all event categories, as defined in Oke et al. [34], in wild-type, *mlh3-23*, *mlh3-32*, *mlh3-D523N* and *mlh3Δ*.
(PDF)

Acknowledgments

We are grateful to Michael Lichten, Nancy Kleckner, Alba Guarne, Duyen Bui, Ujani Chakraborty, and Christopher Furman for fruitful discussions, Scott Keeney for reagents, Nathan Kruer-Zerhusen for help constructing the *S. cerevisiae* Mlh3 homology model, and Erika Mudrak from the Cornell Statistical Consulting Unit for help with some of the statistical analyses. We also thank Scott Keeney and Corentin Claeys Bouuaert for insightful comments and for sharing unpublished data, anonymous reviewers for extremely helpful comments, and Carol Anderson and Jennifer Fung for providing us with advice on using groupEvents.

Author Contributions

Conceptualization: Najla Al-Sweel, Vandana Raghavan, Abhishek Dutta, V. P. Ajith, Carol M. Manhart, Jennifer A. Surtees, K. T. Nishant, Eric Alani.

Data curation: Najla Al-Sweel, Vandana Raghavan, Abhishek Dutta, V. P. Ajith, Luigi Di Vietro, Nabila Khondakar, K. T. Nishant, Eric Alani.

Formal analysis: Najla Al-Sweel, Vandana Raghavan, Abhishek Dutta, V. P. Ajith, Luigi Di Vietro, Nabila Khondakar, Carol M. Manhart, K. T. Nishant, Eric Alani.

Funding acquisition: Jennifer A. Surtees, K. T. Nishant, Eric Alani.

Investigation: Najla Al-Sweel, Vandana Raghavan, Abhishek Dutta, V. P. Ajith, K. T. Nishant, Eric Alani.

Methodology: Najla Al-Sweel, Vandana Raghavan, Abhishek Dutta, V. P. Ajith, Carol M. Manhart, Jennifer A. Surtees, K. T. Nishant, Eric Alani.

Project administration: K. T. Nishant, Eric Alani.

Resources: Jennifer A. Surtees, K. T. Nishant.

Software: Abhishek Dutta, V. P. Ajith, K. T. Nishant.

Supervision: Najla Al-Sweel, Carol M. Manhart, K. T. Nishant, Eric Alani.

Validation: Najla Al-Sweel, Abhishek Dutta, V. P. Ajith, Carol M. Manhart, K. T. Nishant, Eric Alani.

Visualization: Najla Al-Sweel, Vandana Raghavan.

Writing – original draft: Najla Al-Sweel, Vandana Raghavan, Abhishek Dutta, Carol M. Manhart, K. T. Nishant, Eric Alani.

Writing – review & editing: Najla Al-Sweel, Vandana Raghavan, Abhishek Dutta, V. P. Ajith, Carol M. Manhart, Jennifer A. Surtees, K. T. Nishant, Eric Alani.

References

1. Kunkel TA, Erie DA (2015) Eukaryotic Mismatch Repair in Relation to DNA Replication. Annu Rev Genet 49: 291–313. https://doi.org/10.1146/annurev-genet-112414-054722 PMID: 26436461
2. Flores-Rozas H, Kolodner RD (1998) The *Saccharomyces cerevisiae MLH3* gene functions in *MSH3*-dependent suppression of frameshift mutations. Proc Natl Acad Sci USA 95: 12404–12409. PMID: 9770499
3. Harfe BD, Minesinger BK, Jinks-Robertson S (2000) Discrete in vivo roles for the MutL homologs Mlh2p and Mlh3p in the removal of frameshift intermediates in budding yeast. Curr Biol 10: 145–148. PMID: 10679328

4. Romanova NV, Crouse GF (2013) Different roles of eukaryotic MutS and MutL complexes in repair of small insertion and deletion loops in yeast. PLoS Genet 9: e1003920. https://doi.org/10.1371/journal.pgen.1003920 PMID: 24204320
5. Nishant KT, Plys AJ, Alani E (2008) A mutation in the putative MLH3 endonuclease domain confers a defect in both mismatch repair and meiosis in *Saccharomyces cerevisiae*. Genetics 179: 747–755. https://doi.org/10.1534/genetics.108.086645 PMID: 18505871
6. Sonntag Brown M, Lim E, Chen C, Nishant KT, Alani E (2013) Genetic analysis of *mlh3* mutations reveals interactions between crossover promoting factors during meiosis in baker's yeast. G3 3: 9–22. https://doi.org/10.1534/g3.112.004622 PMID: 23316435
7. Argueso JL, Wanat J, Gemici Z, Alani E (2004) Competing crossover pathways act during meiosis in *Saccharomyces cerevisiae*. Genetics 168: 1805–1816. https://doi.org/10.1534/genetics.104.032912 PMID: 15611158
8. Zakharyevich K, Tang S, Ma Y, Hunter N (2012) Delineation of joint molecule resolution pathways in meiosis identifies a crossover-specific resolvase. Cell 149: 334–347. https://doi.org/10.1016/j.cell.2012.03.023 PMID: 22500800
9. Hunter N (2006) Meiotic Recombination, in: Mol Genet Recomb Top Curr Genet, 17th ed., pp. 381–442.
10. Zickler D (2006) From early homologue recognition to synaptonemal complex formation. Chromosoma 115: 158–174. https://doi.org/10.1007/s00412-006-0048-6 PMID: 16570189
11. Champion MD, Hawley RS (2002) Playing for half the deck: The molecular biology of meiosis. Nat Cell Biol 4 Suppl: s50–56.
12. Maguire MP (1974) Letter: The need for a chiasma binder. J Theor Biol 48: 485–487. PMID: 4459599
13. Hassold T, Hunt P (2001) To err (meiotically) is human: the genesis of human aneuploidy. Nat Rev Genet 2: 280–291. https://doi.org/10.1038/35066065 PMID: 11283700
14. Kadyrov FA, Dzantiev L, Constantin N, Modrich P (2006) Endonucleolytic function of MutLalpha in human mismatch repair. Cell 126: 297–308. https://doi.org/10.1016/j.cell.2006.05.039 PMID: 16873062
15. Kadyrov FA, Holmes SF, Arana ME, Lukianova OA, O'Donnell M, Kunkel TA, et al. (2007) *Saccharomyces cerevisiae* MutLalpha is a mismatch repair endonuclease. J Biol Chem 282: 37181–37190. https://doi.org/10.1074/jbc.M707617200 PMID: 17951253
16. Rogacheva MV, Manhart CM, Chen C, Guarne A, Surtees J, Alani E (2014) Mlh1-Mlh3, A meiotic crossover and DNA mismatch repair factor, is a Msh2-Msh3-stimulated endonuclease. J Biol Chem 289: 5664–5673. https://doi.org/10.1074/jbc.M113.534644 PMID: 24403070
17. Ranjha L, Anand R, Cejka P (2014) The *Saccharomyces cerevisiae* Mlh1-Mlh3 heterodimer is an endonuclease that preferentially binds to Holliday Junctions. J Biol Chem 289: 5674–5686. https://doi.org/10.1074/jbc.M113.533810 PMID: 24443562
18. Szostak JW, Orr-Weaver TL, Rothstein RJ, Stahl FW (1983) The double-strand-break repair model for recombination. Cell 33: 25–35. PMID: 6380756
19. Robine N, Uematsu N, Amiot F, Gidrol X, Barillot E, et al. (2007) Genome-wide redistribution of meiotic double-strand breaks in *Saccharomyces cerevisiae*. Mol Cell Biol 275: 1868–1880.
20. Cao L, Alani E, Kleckner N (1990) A pathway for generation and processing of double-strand breaks during meiotic recombination in *S. cerevisiae*. Cell 61: 1089–1101. PMID: 2190690
21. Gilbertson LA, Stahl FW (1996) A test of the double-strand break repair model for meiotic recombination in *Saccharomyces cerevisiae*. Genetics 144: 27–41. PMID: 8878671
22. Keeney S, Giroux CN, Kleckner N (1997) Meiosis-specific DNA double-strand breaks are catalyzed by Spo11, a member of a widely conserved protein family. Cell 88: 357–384.
23. Chen SY, Tsubouchi T, Rockmill B, Sandler JS, Richards DR, et al. (2008) Global analysis of the meiotic crossover landscape. Dev Cell 15: 401–415. https://doi.org/10.1016/j.devcel.2008.07.006 PMID: 18691940
24. Schwacha A, Kleckner N (1995) Identification of double Holliday junctions as intermediates in meiotic recombination. Cell 83: 783–791. PMID: 8521495
25. Hunter N, Kleckner N (2001) The single-end invasion: an asymmetric intermediate at the double-strand break to double-Holliday Junction transition of meiotic recombination. Cell 106: 59–70. PMID: 11461702
26. Allers T, Lichen M (2001a) Intermediates of yeast meiotic recombination contain heteroduplex DNA. Mol Cell 8: 225–231.
27. Allers T, Lichten M (2001b) Differential timing and control of noncrossover and crossover recombination during meiosis. Cell 106: 47–57.

28. Börner GV, Kleckner N, Hunter N (2004) Crossover/noncrossover differentiation, synaptonemal complex formation, and regulatory surveillance at the leptotene/zygotene transition of meiosis. Cell 117: 29–45. PMID: 15066280

29. Zakharyevich K, Ma Y, Tang S, Hwang PY, Boiteux S, et al. (2010) Temporally and biochemically distinct activities of Exo1 during meiosis: double-strand break resection and resolution of double Holliday Junctions. Mol Cell 40: 1001–1015. https://doi.org/10.1016/j.molcel.2010.11.032 PMID: 21172664

30. Kaur H, De Muyt A, Lichten M (2015) Top3-Rmi1 DNA single-strand decatenase is integral to the formation and resolution of meiotic recombination intermediates. Mol Cell 57: 583–594. https://doi.org/10.1016/j.molcel.2015.01.020 PMID: 25699707

31. Tang S Wu MKY, Zhang R, Hunter N (2015) Pervasive and essential roles of the Top3-Rmi1 decatenase orchestrate recombination and facilitate chromosome segregation in meiosis. Mol Cell 57: 607–621. https://doi.org/10.1016/j.molcel.2015.01.021 PMID: 25699709

32. de Los Santos T, Hunter N, Lee C., Larkin B, Loidl J, et al. (2003) The Mus81/Mms4 endonuclease acts independently of double-Holliday junction resolution to promote distinct subset of crossovers during meiosis in budding yeast. Genetics 164: 81–94. PMID: 12750322

33. Matos J, Blanco MG, Maslen S, Skehel JM, West SC (2011) Regulatory control of the resolution of DNA recombination intermediates during meiosis and mitosis. Cell 147: 158–172. https://doi.org/10.1016/j.cell.2011.08.032 PMID: 21962513

34. Oke A, Anderson CM, Yam P, Fung JC (2014) Controlling meiotic recombinational repair—specifying the roles of ZMMs, Sgs1 and Mus81/Mms4 in crossover formation., PLoS Genet 10: e1004690. https://doi.org/10.1371/journal.pgen.1004690 PMID: 25329811

35. Hunter N, Borts RH (1997) Mlh1 is unique among mismatch repair proteins in its ability to promote crossing-over during meiosis. Genes Dev 11: 1573–1582. PMID: 9203583

36. Wang TF, Kleckner N, Hunter N (1999) Functional specificity of MutL homologs in yeast: evidence for three Mlh1-based heterocomplexes ith distinct roles during meiosis in recombination and mismatch correction. Proc Natl Acad Sci USA 96: 13914–13919. PMID: 10570173

37. Lipkin SM, Moens PB, Wang V, Lenzi M, Shanmugarajah D, Gilgeous A, et al. (2002) Meiotic arrest and aneuploidy in MLH3-deficient mice., Nat Genet 31: 385–390. https://doi.org/10.1038/ng931 PMID: 12091911

38. Woods LM, Hodges CA, Baart E, Baker SM, Liskay M, Hunt PA (1999). Chromosomal influence on meiotic spindle assembly: abnormal meiosis I in female Mlh1 mutant mice. J Cell Biol 145: 1395–1406. PMID: 10385520

39. Svetlanov A, Baudat F, Cohen PE, de Massy B (2008) Distinct functions of MLH3 at recombination hot spots in the mouse. Genetics 178: 1937–1945. https://doi.org/10.1534/genetics.107.084798 PMID: 18430927

40. Santucci-Darmanin S, Walpita D, Lespinasse F, Desnuelle C, Ashley T, et al. (2000) MSH4 acts in conjunction with MLH1 during mammalian meiosis. FASEB J 14: 1539–1547. PMID: 10928988

41. Santucci-Darmanin S, Neyton S, Lespinasse F, Saunieres A, Gaudray P, et al. (2002) The DNA mismatch-repair MLH3 protein interacts with Msh4 in meiotic cells, supporting a role for this MutL homolog in mammalian meiotic recombination. Hum Mol Genet 11: 1697–1706. PMID: 12095912

42. Wang T-F, Kung W-M (2002) Supercomplex formation between Mlh1-Mlh3 and Sgs1-Top3 heterocomplexes in meiotic yeast cells. Biochem Biophys Res Commun 296: 949–953. PMID: 12200140

43. Snowden T, Acharya S, Butz C, Berardini M, Fishel R (2004) hMSH4-hMSH5 recognizes Holliday Junctions and forms a meiosis-specific sliding clamp that embraces homologous chromosomes. Mol Cell 15: 437–451. https://doi.org/10.1016/j.molcel.2004.06.040 PMID: 15304223

44. Kneitz B, Cohen PE, Avdievich E, Zhu L, Kane MF, et al. (2000) MutS homolog 4 localization to meiotic chromosomes is required for chromosome pairing during meiosis in male and female mice. Genes Dev 14: 1085–1097. PMID: 10809667

45. Hoffmann ER, Borts RH (2004) Meiotic recombination intermediates and mismatch repair proteins. Cytogenet Genome Res 107: 232–248. https://doi.org/10.1159/000080601 PMID: 15467368

46. Fasching CL, Cejka P, Kowalczykowski SC, Heyer W-D (2015) Top3-Rmi1 Dissolve Rad51-Mediated D Loops by a Topoisomerase-Based Mechanism, Mol Cell 57: 595–606. https://doi.org/10.1016/j.molcel.2015.01.022 PMID: 25699708

47. Kolas NK, Cohen PE (2004) Novel and diverse functions of the DNA mismatch repair family in mammalian meiosis and recombination. Cytogenet Genome Res 107: 216–231. https://doi.org/10.1159/000080600 PMID: 15467367

48. Kolas NK, Svetlanov A, Lenzi ML, Macaluso FP, Lipkin SM, Liskay RM, et al. (2005) Localization of MMR proteins on meiotic chromosomes in mice indicates distinct functions during prophase I. J Cell Biol 171: 447–458. https://doi.org/10.1083/jcb.200506170 PMID: 16260499

49. de Muyt A, Jessop L, Kolar E, Sourirajan A, Chen J et al. (2012) BLM helicase ortholog Sgs1 is a central regulator of meiotic recombination intermediate metabolism. Mol. Cell 46: 42–53.

50. Manhart CM, Alani E (2016) Roles for mismatch repair family proteins in promoting meiotic crossing over. DNA Repair (Amst) 38: 84–93. https://doi.org/10.1016/j.dnarep.2015.11.024 PMID: 26686657

51. Gueneau E, Dherin C, Legrand P, Tellier-Lebegue C, Gilquin B, Bonnesoeur P, et al. (2013) Structure of the MutLα C-terminal domain reveals how Mlh1 contributes to Pms1 endonuclease site., Nat Struct Mol Biol 20: 461–468. https://doi.org/10.1038/nsmb.2511 PMID: 23435383

52. Bass SH, Mulkerrin MG, Wells JA (1991) A systematic mutational analysis of hormone-binding determinants in the human growth hormone receptor. Proc Natl Acad Sci USA 88: 4498–4502. PMID: 2034689

53. Bennett WF, Paoni NF, Keyt BA, Botstein D, Jones AJ, et al. (1991) High resolution analysis of functional determinants on human tissue-type plasminogen activator. J Biol Chem 266: 5191–5201. PMID: 1900516

54. Argueso JL, Kijas AW, Sarin A, Heck J, Waase M, et al. (2003) Systematic mutagenesis of the *Saccharomyces cerevisiae* MLH1 gene reveals distinct roles for Mlh1p in meiotic crossing over and in vegetative and meiotic mismatch repair. Mol Cell Biol 23: 873–886. https://doi.org/10.1128/MCB.23.3.873-886.2003 PMID: 12529393

55. Tran HT, Keen JD, Kricker M, Resnick MA, Gordenin DA (1997) Hypermutability of homonucleotide runs in mismatch repair and DNA polymerase proofreading yeast mutants. Mol Cell Biol 17: 2859–2865. PMID: 9111358

56. Thacker D, Lam I, Knop M, Keeney S. (2011) Exploiting spore-autonomous fluorescent protein expression to quantify meiotic chromosome behaviors in *Saccharomyces cerevisiae*. Genetics 189: 423–439. https://doi.org/10.1534/genetics.111.131326 PMID: 21840861

57. Groothuizen FS, Winkler I, Cristóvão M, Fish A, Winterwerp HH, Reumer A, Marx AD, Hermans N, Nicholls RA, Murshudov GN, Lebbink JH, Friedhoff P, Sixma TK (2015) MutS/MutL crystal structure reveals that the MutS sliding clamp loads MutL onto DNA. Elife 4: e06744. https://doi.org/10.7554/eLife.06744 PMID: 26163658

58. Hargreaves VV, Putnam CD, Kolodner RD (2012) Engineered disulfide-forming amino acid substitutions interfere with a conformational change in the mismatch recognition complex Msh2-Msh6 required for mismatch repair. J Biol Chem 287: 41232–41244. https://doi.org/10.1074/jbc.M112.402495 PMID: 23045530

59. Kosinski J, Plotz G, Guarné A, Bujnicki JM, Friedhoff P (2008) The PMS2 subunit of human MutLalpha contains a metal ion binding domain of the iron-dependent repressor protein family. J Mol Biol 382: 610–627. https://doi.org/10.1016/j.jmb.2008.06.056 PMID: 18619468

60. Pillon MC, Lorenowicz JJ, Uckelmann M, Klocko AD, Mitchell RR, Chung YS, et al. (2010) Structure of the endonuclease domain of MutL: unlicensed to cut. Mol Cell 39: 145–151. https://doi.org/10.1016/j.molcel.2010.06.027 PMID: 20603082

61. Argueso JL, Smith D, Yi J, Waase M, Sarin S, Alani E (2002) Analysis of conditional mutations in the *Saccharomyces cerevisiae MLH1* gene in mismatch repair and in meiotic crossing over., Genetics 160: 909–921. PMID: 11901110

62. Agarwal S, Roeder GS (2000) Zip3 provides a link between recombination enzymes and synaptonemal complex proteins., Cell 102: 245–255. PMID: 10943844

63. Nishant KT, Chen C, Shinohara M, Shinohara A, Alani E (2010) Genetic analysis of baker's yeast Msh-Msh5 reveals a threshold crossover level for meiotic viability. PLoS Genet 6: e1001083. https://doi.org/10.1371/journal.pgen.1001083 PMID: 20865162

64. Krishnaprasad GN, Anand MT, Lin G, Tekkedil MM, Steinmetz LM, Nishant KT (2015). Variation in crossover frequencies perturb crossover assurance without affecting meiotic chromosome segregation in *Saccharomyces cerevisiae*. Genetics 199: 399–412. https://doi.org/10.1534/genetics.114.172320 PMID: 25467183

65. Martini E, Diaz RL, Hunter N, Keeney S (2006) Crossover homeostasis in yeast meiosis. Cell 126: 285–295. https://doi.org/10.1016/j.cell.2006.05.044 PMID: 16873061

66. Mancera E, Bourgon R, Brozzi A, Huber W, Steinmetz LM (2008) High-resolution mapping of meiotic crossovers and non-crossovers in yeast. Nature 454: 479–485. https://doi.org/10.1038/nature07135 PMID: 18615017

67. Cotton VE, Hoffmann ER, Borts RH (2010) Distinct Regulation of Mlh1p Heterodimers in Meiosis and Mitosis in Saccharomyces cerevisiae. Genetics 185: 459–467. https://doi.org/10.1534/genetics.110.116806 PMID: 20382827

68. Greig D, Travisano M, Louis EJ, Borts RH (2003) A role for the mismatch repair system during incipient speciation in Saccharomyces. J Evol Biol 16: 429–437. PMID: 14635842

69. Pan J, Sasaki M, Kniewel R, Murakami H, Blitzblau HG, Tischfield SE, Zhu X, Neale MJ, Jasin M, Socci ND, Hochwagen A, Keeney S (2011) A hierarchical combination of factors shapes the genome-wide topography of yeast meiotic recombination initiation. Cell 144: 719–731. https://doi.org/10.1016/j.cell.2011.02.009 PMID: 21376234

70. Chakraborty P., Ajith V.P., Lin G, Dutta A., Krishnaprasad G.N., Tekkedil M.M., Shinohara A., Steinmetz L.M. and Nishant K.T (2017) Modulating crossover frequency and interference for obligate crossovers in *Saccharomyces cerevisiae* meiosis. G3 7: 1511–1524. https://doi.org/10.1534/g3.117.040071 PMID: 28315832

71. Thacker D, Mohibullah N, Zhu X, Keeney S (2014) Homologue engagement controls meiotic DNA break number and distribution. Nature 510: 241–246. https://doi.org/10.1038/nature13120 PMID: 24717437

72. Gray S, Allison RM, Garcia V, Goldman AS, Neale MJ (2013) Positive regulation of meiotic DNA double-strand break formation by activation of the DNA damage checkpoint kinase Mec1(ATR). Open Biol 3: 130019. https://doi.org/10.1098/rsob.130019 PMID: 23902647

73. Jessop L, Rockmill B, Roeder GS, Lichten M (2006) Meiotic chromosome synapsis-promoting proteins antagonize the anti-crossover activity of Sgs1. PLoS Genet 2: e155. https://doi.org/10.1371/journal.pgen.0020155 PMID: 17002499

74. Manhart CM, Ni X, White MA, Ortega J, Surtees JA, Alani E (2017) The mismatch repair and meiotic recombination endonuclease Mlh1-Mlh3 is activated by polymer formation and can cleave DNA substrates in trans. PLoS Biol 15: e2001164. https://doi.org/10.1371/journal.pbio.2001164 PMID: 28453523

75. Hombauer H, Campbell CS, Smith CE, Desai A, Kolodner RD (2011) Visualization of eukaryotic DNA mismatch repair reveals distinct recognition and repair intermediates. Cell 147: 1040–1053. https://doi.org/10.1016/j.cell.2011.10.025 PMID: 22118461

76. Pluciennik A, Dzantiev L, Iyer RR, Constantin N, Kadyrov FA, Modrich P (2010) PCNA function in the activation and direction of MutLa endonuclease in mismatch repair. Proc Natl Acad Sci USA 107: 16066–16071. https://doi.org/10.1073/pnas.1010662107 PMID: 20713735

77. Pedrazzi G, Perrera C, Blaser H, Kuster P, Marra G, Davies SL, Ryu GH, Freire R, Hickson ID, Jiricny J, Stagljar I (2001) Direct association of Bloom's syndrome gene product with the human mismatch repair protein MLH1. Nucleic Acids Res 29: 4378–4386. PMID: 11691925

78. Duroc Y, Kumar R, Ranjha L Adam C, Guérois R, Md Muntaz K, et al. (2017) Concerted action of the MutLβ heterodimer and Mer3 helicase regulates the global extent of meiotic gene conversion. Elife 6: e21900. https://doi.org/10.7554/eLife.21900 PMID: 28051769

79. Sacho EJ, Kadyrov FA, Modrich P, Kunkel TA, Erie DA (2008) Direct visualization of asymmetric adenine-nucleotide-induced conformational changes in MutL alpha. Mol Cell 29: 112–121. https://doi.org/10.1016/j.molcel.2007.10.030 PMID: 18206974

80. Plys AJ, Rogacheva MV, Greene EC, Alani E (2012) The unstructured linker arms of Mlh1-Pms1 are important for interactions with DNA during mismatch repair. J Mol Biol 422: 192–203. https://doi.org/10.1016/j.jmb.2012.05.030 PMID: 22659005

81. Claeys Bouuaert C, Keeney S (2017) Distinct DNA-binding surfaces in the ATPase and linker domains of MutLγ determine its substrate specificities and exert separable functions in meiotic recombination and mismatch repair. PLoS Genet 13: e1006722. https://doi.org/10.1371/journal.pgen.1006722 PMID: 28505149

82. Ban C Junop M, Yang W (1999) Transformation of MutL by ATP binding and hydrolysis: a switch in DNA mismatch repair. Cell 97: 85–97. PMID: 10199405

83. Pashaiefar H, Sheikhha MH, Kalantar SM, Jahaninejad T, Zaimy MA, Ghasemi N (2013) Analysis of *MLH3* C2531T polymorphism in Iranian women with unexplained infertility, Iranian Journal of Reproductive Medicine, 11: 19–24. PMID: 24639688

84. Markandona O, Dafopoulos K, Anifandis G, Messini CI, Dimitraki M, Tsezou A, Georgoulias P, Messinis IE (2015) Single-nucleotide polymorphism rs 175080 in the *MLH3* gene and its relation to male infertility. J Assist Reprod Genet 32: 1795–1799. https://doi.org/10.1007/s10815-015-0594-z PMID: 26520453

85. Zhang X, Ding M, Ding X, Li T, Chen H (2015) Six polymorphisms in genes involved in DNA double-strand break repair and chromosome synapsis: association with male infertility. Syst Biol Reprod Med 61: 187–193. https://doi.org/10.3109/19396368.2015.1027014 PMID: 26086992

86. Rose MD, Winston F, Hieter P (1990) Methods in yeast genetics: A Laboratory Course Manual. Cold Spring Harbor Laboratory Press, Cold Spring Harbor, NY.

87. Wach A. Brachat A, Pohlmann R, Philippsen P (1994) New heterologous modules for classical or PCR-based gene disruptions in *Saccharomyces cerevisiae.* Yeast 10: 1793–1808. PMID: 7747518

88. Goldstein AL, McCusker JH (1999) Three new dominant drug resistance cassettes for gene disruption in *Saccharomyces cerevisiae*. Yeast 15: 1541–1553. https://doi.org/10.1002/(SICI)1097-0061(199910)15:14<1541::AID-YEA476>3.0.CO;2-K PMID: 10514571

89. Gietz RD, Schiestl RH, Willems AR, Woods RA (1995) Studies on the transformation of intact yeast cells by the LiAc/SS-DNA/PEG procedure. Yeast 11: 355–360. https://doi.org/10.1002/yea.320110408 PMID: 7785336

90. Heck JA, Argueso JL, Gemici Z, Reeves RG, Bernard A, et al. (2006) Negative epistasis between natural variants of the *Saccharomyces cerevisiae* MLH1 and PMS1 genes results in a defect in mismatch repair. Proc Natl Acad Sci USA 103: 3256–3261. https://doi.org/10.1073/pnas.0510998103 PMID: 16492773

91. Drake JW (1991) A constant rate of spontaneous mutation in DNA-based microbes. Proc Natl Acad Sci USA 88: 7160–7164. PMID: 1831267

92. Gibbons JD, Chakraborti S (2014) Nonparametric Statistical Inference, Fourth Edition. Marcel Dekker, Inc., New York.

93. Sonntag Brown M, Zanders S, Alani E (2011) Sustained and rapid chromosome movements are critical for chromosome pairing and meiotic progression in budding yeast. Genetics 188: 21–32. https://doi.org/10.1534/genetics.110.125575 PMID: 21339478

94. Pang Q, Prolla TA, Liskay RM (1997) Functional domains of the *Saccharomyces cerevisiae* Mlh1p and Pms1p DNA mismatch repair proteins and their relevance to human hereditary nonpolyposis colorectal cancer-associated mutations. Mol Cell Biol 17: 4465–4473. PMID: 9234704

95. Gietz RD, Woods RA (2002) Screening for protein-protein interactions in the yeast two-hybrid system. Methods Mol Biol 185: 471–486. PMID: 11769011

96. Bradford MM (1976) A rapid and sensitive method for the quantitation of microgram quantities of protein utilizing the principle of protein-dye binding. Anal Biochem 72: 248–254. PMID: 942051

97. Surtees JA, Alani E (2006) Mismatch repair factor MSH2-MSH3 binds and alters the conformation of branched DNA structures predicted to form during genetic recombination. J Mol Biol 360: 523–536. https://doi.org/10.1016/j.jmb.2006.05.032 PMID: 16781730

98. Perkins DD (1949) Biochemical mutants in the smut fungus *Ustilago maydis*. Genetics 34: 607–626. PMID: 17247336

99. Anderson CM, Chen SY, Dimon MT, Oke A, DeRisi JL, Fung JC (2011) ReCombine: a suite of programs for detection and analysis of meiotic recombination in whole-genome datasets. PLoS One 6: e25509. https://doi.org/10.1371/journal.pone.0025509 PMID: 22046241
